# Supplementary figures and images for: Prevalence, genotype distribution and mutations of hepatitis B virus and the associated risk factors among pregnant women residing in the northern shores of Persian Gulf, Iran
Source: PLoS One. 2022 Mar 10;17(3):e0265063. doi: 10.1371/journal.pone.0265063 (PMC8912131; doi:10.1371/journal.pone.0265063)

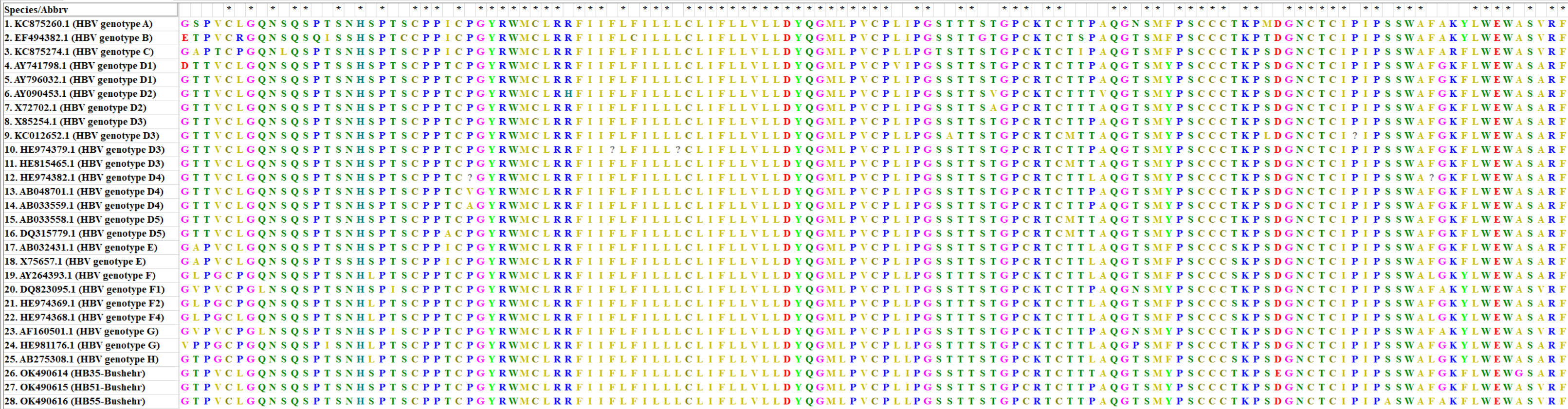

Supplement: S1 Fig — (TIF) [file pone.0265063.s001.tif]

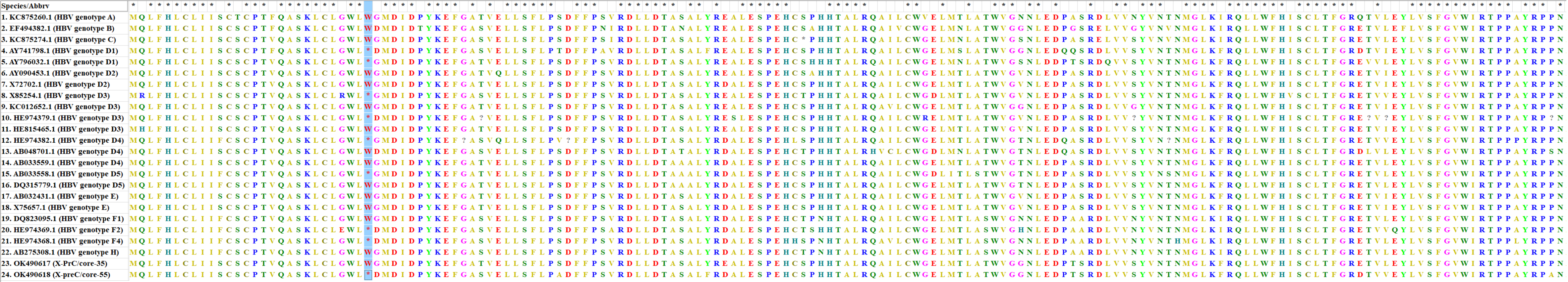

Supplement: S2 Fig — (TIF) [file pone.0265063.s002.tif]

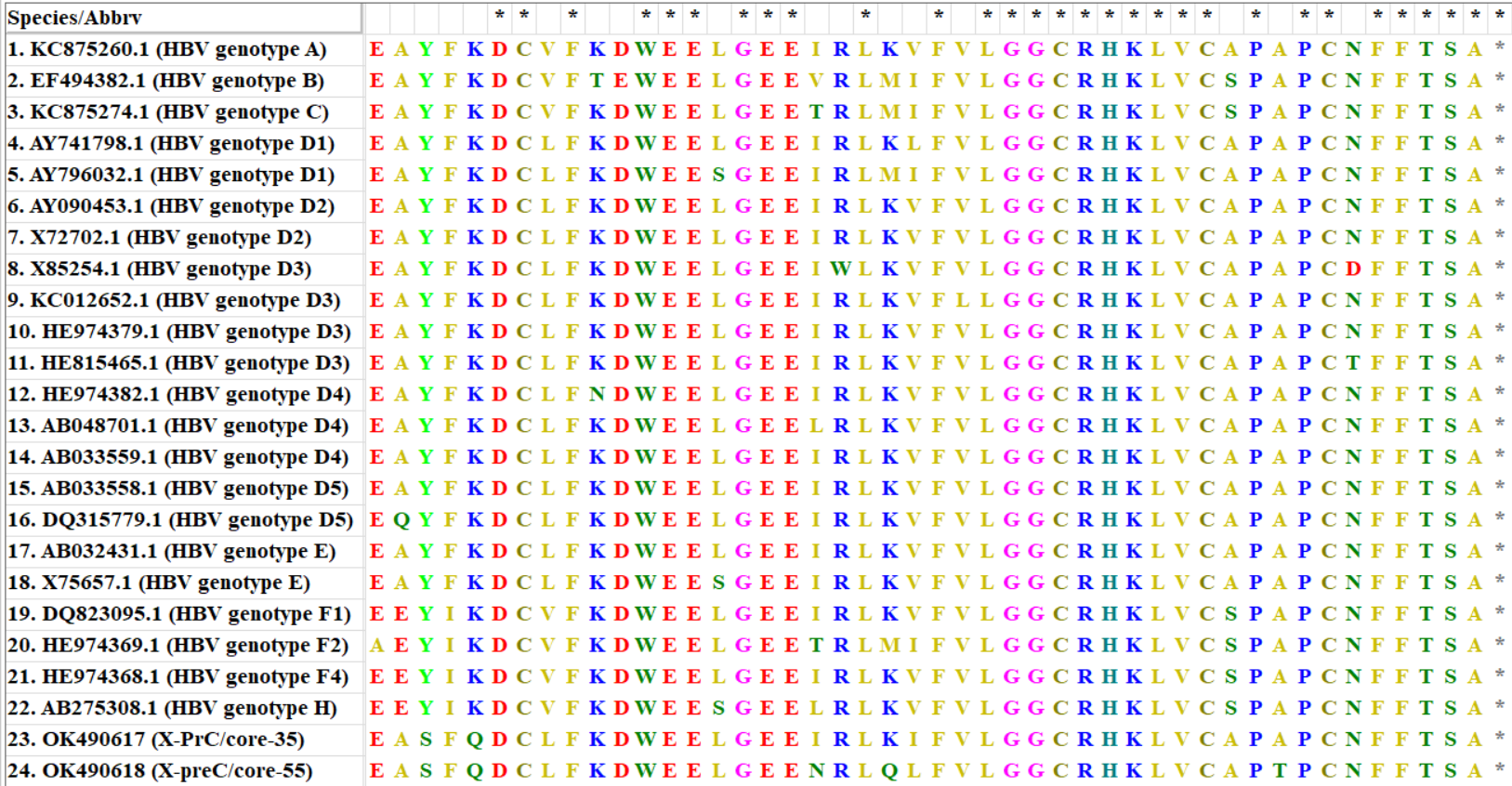

Supplement: S3 Fig — (TIF) [file pone.0265063.s003.tif]
