# Supplementary material for: Prevalence, genotype distribution and mutations of hepatitis B virus and the associated risk factors among pregnant women residing in the northern shores of Persian Gulf, Iran
Source: PLoS One. 2022 Mar 10;17(3):e0265063. doi: 10.1371/journal.pone.0265063 (PMC8912131; doi:10.1371/journal.pone.0265063)
Supplement: S1 Table — (DOC) [file pone.0265063.s004.doc]

S1 Table. Prevalence of HBsAg according to socio-demographic and qualitative variables among pregnant women in South of Iran

|  | **No. of all participants (%):1425 (100%)** | **No. of HBsAg negative subjects (%):1410 (98.9%)** | **No. of HBsAg positive subjects (%):15 (1.05%)** | **P-Value** |
| --- | --- | --- | --- | --- |
| **Age groups (years)** |  |  |  | **0.425** |
| <20 | 108 (7.6%) | 108 (100.0%) | 0 (0.0%) |  |
| 20-29 | 786 (55.2%) | 775 (98.6%) | 11 (1.4%) |  |
| 30-39 | 485 (34.0%) | 481 (99.2%) | 4 (0.8%) |  |
| >39 | 46 (3.2%) | 46 (100.0%) | 0 (0.0%) |  |
| **Place of residence (city)** |  |  |  | **0.292** |
| Bushehr | 616 (43.2%) | 612 (99.4%) | 4 (0.6%) |  |
| Borazjan | 440 (30.9%) | 432 (98.2%) | 8 (1.8%) |  |
| Ahram | 207 (14.5%) | 206 (99.5%) | 1 (0.5%) |  |
| Jam | 122 (8.6%) | 121 (99.2%) | 1 (0.8%) |  |
| Khormuj | 40 (2.8%) | 39 (97.5%) | 1 (2.5%) |  |
| **Ethnicity** |  |  |  | **0.788** |
| Fars | 1283 (90.0%) | 1269 (98.9%) | 14 (1.1%) |  |
| Arab | 68 (4.8%) | 68 (100.0%) | 0 (0.0%) |  |
| Afghan | 62 (4.4%) | 61 (98.4%) | 1 (1.6%) |  |
| Turk | 12 (0.8%) | 12 (100.0%) | 0 (0.0%) |  |
| **Stage of gestation** |  |  |  | **0.308** |
| First trimester | 256 (18.0%) | 255 (99.6%) | 1 (0.4%) |  |
| Second trimester | 194 (13.6%) | 193 (99.5%) | 1 (0.5%) |  |
| Third trimester | 975 (68.4%) | 962 (98.7%) | 13 (1.3%) |  |
| **Number of Pregnancies** |  |  |  | **0.711** |
| One pregnancy | 440 (30.9%) | 435 (98.9%) | 5 (1.1%) |  |
| Two pregnancies | 785 (55.1%) | 776 (98.9%) | 9 (1.1%) |  |
| Three and more than three pregnancies | 200 (14.0%) | 199 (99.5%) | 1 (0.5%) |  |
| **History of Abortion** |  |  |  | **0.948** |
| No | 934 (65.5%) | 924 (98.9%) | 10 (1.1%) |  |
| Yes | 231 (16.2%) | 229 (99.1%) | 2 (0.9%) |  |
| Unknown | 260 (18.2%) | 257 (98.8%) | 3 (1.2%) |  |
| **Education** |  |  |  | **0.535** |
| Upper diploma | 366 (25.7%) | 364 (99.5%) | 2 (0.5%) |  |
| Diploma | 677 (47.5%) | 670 (99.0%) | 7 (1.0%) |  |
| Under diploma | 340 (23.9%) | 335 (98.5%) | 5 (1.5%) |  |
| Illiterate | 42 (2.9%) | 41 (97.6%) | 1 (2.4%) |  |
| **Year** |  |  |  | **1.0** |
| 2018 | 797 (55.9%) | 789 (99.0%) | 8 (1.0%) |  |
| 2019 | 628 (44.1%) | 621 (98.9%) | 7 (1.1%) |  |
| **Month** |  |  |  | **0.505** |
| Oct | 113 (7.9%) | 112 (99.1%) | 1 (0.9%) |  |
| Nov | 102 (7.2%) | 100 (98.0%) | 2 (2.0%) |  |
| Dec | 112 (7.9%) | 111 (99.1%) | 1 (0.9%) |  |
| Jan | 108 (7.6%) | 106 (98.1%) | 2 (1.9%) |  |
| Feb | 291 (20.4%) | 288 (99.0%) | 3 (1.0%) |  |
| Mar | 248 (17.4%) | 247 (99.6%) | 1 (0.4%) |  |
| Apr | 131 (9.2%) | 130 (99.2%) | 1 (0.8%) |  |
| May | 168 (11.8%) | 168 (100.0%) | 0 (0.0%) |  |
| June | 123 (8.6%) | 120 (97.6%) | 3 (2.4%) |  |
| July | 29 (2.0%) | 28 (96.6%) | 1 (3.4%) |  |
| **Smoking** |  |  |  | **0.572** |
| No | 836 (58.7%) | 828 (99.0%) | 8 (1.0%) |  |
| Yes | 62 (4.4%) | 62 (100.0%) | 0 (0.0%) |  |
| Unknown | 527 (37.0%) | 520 (98.7%) | 7 (1.3%) |  |
| **History of blood transfusion** |  |  |  | **0.784** |
| No | 892 (62.6%) | 883 (99.0%) | 9 (1.0%) |  |
| Yes | 18 (1.3%) | 18 (100.0%) | 0 (0.0%) |  |
| Unknown | 515 (36.1%) | 509 (98.8%) | 6 (1.2%) |  |
| **History of surgery** |  |  |  | **0.946** |
| No | 692 (48.6%) | 685 (99.0%) | 7 (1.0%) |  |
| Yes | 218 (15.3%) | 216 (99.1%) | 2 (0.9%) |  |
| Unknown | 515 (36.1%) | 509 (98.8%) | 6 (1.2%) |  |
| **History of tattoo** |  |  |  | **0.728** |
| No | 786 (55.2%) | 779 (99.1%) | 7 (0.9%) |  |
| Yes | 124 (8.7%) | 122 (98.4%) | 2 (1.6%) |  |
| Unknown | 515 (36.1%) | 509 (98.8%) | 6 (1.2%) |  |
| **History of dentistry** |  |  |  | **0.137** |
| No | 504 (35.4%) | 496 (98.4%) | 8 (1.6%) |  |
| Yes | 406 (28.5%) | 405 (99.8%) | 1 (0.2%) |  |
| Unknown | 515 (36.1%) | 509 (98.8 %) | 6 (1.2%) |  |
| **History of HBV vaccination** |  |  |  | **0.433** |
| No | 314 (22.0%) | 312 (98.7%) | 4 (1.3%) |  |
| Yes | 314 (22.0%) | 309 (98.4%) | 5 (1.6%) |  |
| Unknown | 797 (55.9%) | 789 (99.2%) | 6 (0.8%) |  |
