# Supplementary material for: Prevalence, genotype distribution and mutations of hepatitis B virus and the associated risk factors among pregnant women residing in the northern shores of Persian Gulf, Iran
Source: PLoS One. 2022 Mar 10;17(3):e0265063. doi: 10.1371/journal.pone.0265063 (PMC8912131; doi:10.1371/journal.pone.0265063)
Supplement: S2 Table — (DOC) [file pone.0265063.s005.doc]

S2 Table. Prevalence of HBcAb according to socio-demographic and qualitative variables among pregnant women in South of Iran

|  | **No. of all participants (%):1425 (100%)** | **No. of HBcAb negative subjects (%):1384 (97.12%)** | **No. of HBcAb positive subjects (%):41 (2.88%)** | **P-Value** |
| --- | --- | --- | --- | --- |
| **Age groups (years)** |  |  |  | **0.835** |
| <20 | 108 (7.6%) | 106 (98.1%) | 2 (1.9%) |  |
| 20-29 | 786 (55.2%) | 764 (97.2%) | 22 (2.8%) |  |
| 30-39 | 485 (34.0%) | 470 (96.9%) | 15 (3.1%) |  |
| >39 | 46 (3.2%) | 44 (95.7%) | 2 (4.3%) |  |
| **Place of residence (city)** |  |  |  | **0.497** |
| Bushehr | 616 (43.2%) | 601 (97.6%) | 15 (2.4%) |  |
| Borazjan | 440 (30.9%) | 423 (96.1%) | 17 (3.9%) |  |
| Ahram | 207 (14.5%) | 202 (97.6%) | 5 (2.4%) |  |
| Jam | 122 (8.6%) | 118 (96.7%) | 4 (3.3%) |  |
| Khormuj | 40 (2.8%) | 40 (100.0%) | 0 (0.0%) |  |
| **Ethnicity** |  |  |  | **0.195** |
| Fars | 1283 (90.0%) | 1248 (97.3%) | 35 (2.7%) |  |
| Arab | 68 (4.8%) | 67 (98.5%) | 1 (1.5%) |  |
| Afghan | 62 (4.4%) | 58 (93.5%) | 4 (6.5%) |  |
| Turk | 12 (0.8%) | 11 (91.7%) | 1 (8.3%) |  |
| **Stage of gestation** |  |  |  | **0.748** |
| First trimester | 256 (18.0%) | 248 (96.9%) | 8 (3.1%) |  |
| Second trimester | 194 (13.6%) | 187 (96.4%) | 7 (3.6%) |  |
| Third trimester | 975 (68.4%) | 949 (97.3%) | 26 (2.7%) |  |
| **Number of Pregnancies** |  |  |  | **0.091** |
| One pregnancy | 440 (30.9%) | 426 (96.8%) | 14 (3.2%) |  |
| Two pregnancies | 785 (55.1%) | 768 (97.8%) | 17 (2.2%) |  |
| Three and more than three pregnancies | 200 (14.0%) | 190 (95.0%) | 10 (5.0%) |  |
| **History of Abortion** |  |  |  | **0.974** |
| No | 934 (65.5%) | 907 (97.1%) | 27 (2.9%) |  |
| Yes | 231 (16.2%) | 224 (97.0%) | 7 (3.0%) |  |
| Unknown | 260 (18.2%) | 253 (97.3%) | 7 (2.7%) |  |
| **Education** |  |  |  | **0.226** |
| Upper diploma | 366 (25.7%) | 359 (98.1%) | 7 (1.9%) |  |
| Diploma | 677 (47.5%) | 655 (96.8%) | 22 (3.2%) |  |
| Under diploma | 340 (23.9%) | 331 (97.4%) | 9 (2.6%) |  |
| Illiterate | 42 (2.9%) | 39 (92.9%) | 3 (7.1%) |  |
| **Year** |  |  |  | **0.113** |
| 2018 | 797 (55.9%) | 769 (96.5%) | 28 (3.5%) |  |
| 2019 | 628 (44.1%) | 615 (97.9%) | 13 (2.1%) |  |
| **Month** |  |  |  | **0.001** |
| Oct | 113 (7.9%) | 100 (88.5%) | 13 (11.5%) |  |
| Nov | 102 (7.2%) | 100 (98.0%) | 2 (2.0%) |  |
| Dec | 112 (7.9%) | 107 (95.5%) | 5 (4.5%) |  |
| Jan | 108 (7.6%) | 107 (99.1%) | 1 (0.9%) |  |
| Feb | 291 (20.4%) | 288 (99.0%) | 3 (1.0%) |  |
| Mar | 248 (17.4%) | 244 (98.4%) | 4 (1.6%) |  |
| Apr | 131 (9.2%) | 128 (97.7%) | 3 (2.3%) |  |
| May | 168 (11.8%) | 163 (97.0%) | 5 (3.0%) |  |
| June | 123 (8.6%) | 120 (97.6%) | 3 (2.4%) |  |
| July | 29 (2.0%) | 27 (93.1%) | 2 (6.9%) |  |
| **Smoking** |  |  |  | **0.195** |
| No | 836 (58.7%) | 807 (96.5%) | 29 (3.5%) |  |
| Yes | 62 (4.4%) | 62 (100.0%) | 0 (0.0%) |  |
| Unknown | 527 (37.0%) | 515 (97.7%) | 12 (2.3%) |  |
| **History of blood transfusion** |  |  |  | **0.195** |
| No | 892 (62.6%) | 871 (97.6%) | 21 (2.4%) |  |
| Yes | 18 (1.3%) | 18 (100.0%) | 0 (0.0%) |  |
| Unknown | 515 (36.1%) | 495 (96.1%) | 20 (3.9%) |  |
| **History of surgery** |  |  |  | **0.21** |
| No | 692 (48.6%) | 677 (97.8%) | 15 (2.2%) |  |
| Yes | 218 (15.3%) | 212 (97.2%) | 6 (2.8%) |  |
| Unknown | 515 (36.1%) | 495 (96.1%) | 20 (3.9%) |  |
| **History of tattoo** |  |  |  | **0.018** |
| No | 786 (55.2%) | 772 (98.2%) | 14 (1.8%) |  |
| Yes | 124 (8.7%) | 117 (94.4%) | 7 (5.6%) |  |
| Unknown | 515 (36.1%) | 495 (96.1%) | 20 (3.9%) |  |
| **History of dentistry** |  |  |  | **0.148** |
| No | 504 (35.4%) | 490 (97.2%) | 14 (2.8%) |  |
| Yes | 406 (28.5%) | 399 (98.3%) | 7 (1.7%) |  |
| Unknown | 515 (36.1%) | 495 (96.1%) | 20 (3.9%) |  |
| **History of HBV vaccination** |  |  |  | **0.370** |
| No | 314 (22.0%) | 310 (97.5%) | 8 (2.5%) |  |
| Yes | 314 (22.0%) | 308 (98.1%) | 6 (1.9%) |  |
| Unknown | 797 (55.9%) | 766 (96.6%) | 27 (3.4%) |  |
