# Supplementary material for: Prevalence, genotype distribution and mutations of hepatitis B virus and the associated risk factors among pregnant women residing in the northern shores of Persian Gulf, Iran
Source: PLoS One. 2022 Mar 10;17(3):e0265063. doi: 10.1371/journal.pone.0265063 (PMC8912131; doi:10.1371/journal.pone.0265063)
Supplement: S1 Questionnaire — (DOC) [file pone.0265063.s006.doc]

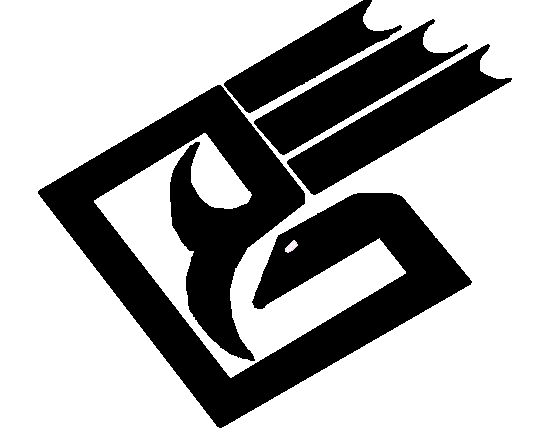
 **Research questionnaire**

**Title:** Prevalence of hepatitis B virus infection among pregnant women

**Research Health Center:** …………………..

**Name:** …………………… **Sample number:** …….. **Date:** …………

|  | Place of residence | |
| --- | --- | --- |
|  | Age | |
|  | Ethnicity ر | |
|  | Education level | |
|  | Stage of gestation (trimester) | |
|  | Number of pregnancies | |
|  | History of abortion | |
|  | History of HBV vaccination | |
|  | History of blood injection | |
|  | History of operation | |
|  | History of dentistry | |
|  | History of tattoo | |
|  | Smoking | |
|  | Injecting drug use | |
|  | AST level (U/L) | |
|  | ALT level (U/L) | |
| The purpose of the study was explained to me orally. I understand the provided information and have had the opportunity to ask questions. I voluntarily agree to take part in this study.  Participant's signature ______________________________ Date __________ | |  |
