# Supplementary material for: Prevalence, genotype distribution and mutations of hepatitis B virus and the associated risk factors among pregnant women residing in the northern shores of Persian Gulf, Iran
Source: PLoS One. 2022 Mar 10;17(3):e0265063. doi: 10.1371/journal.pone.0265063 (PMC8912131; doi:10.1371/journal.pone.0265063)
Supplement: S1 Raw images — (PDF) [file pone.0265063.s007.pdf]

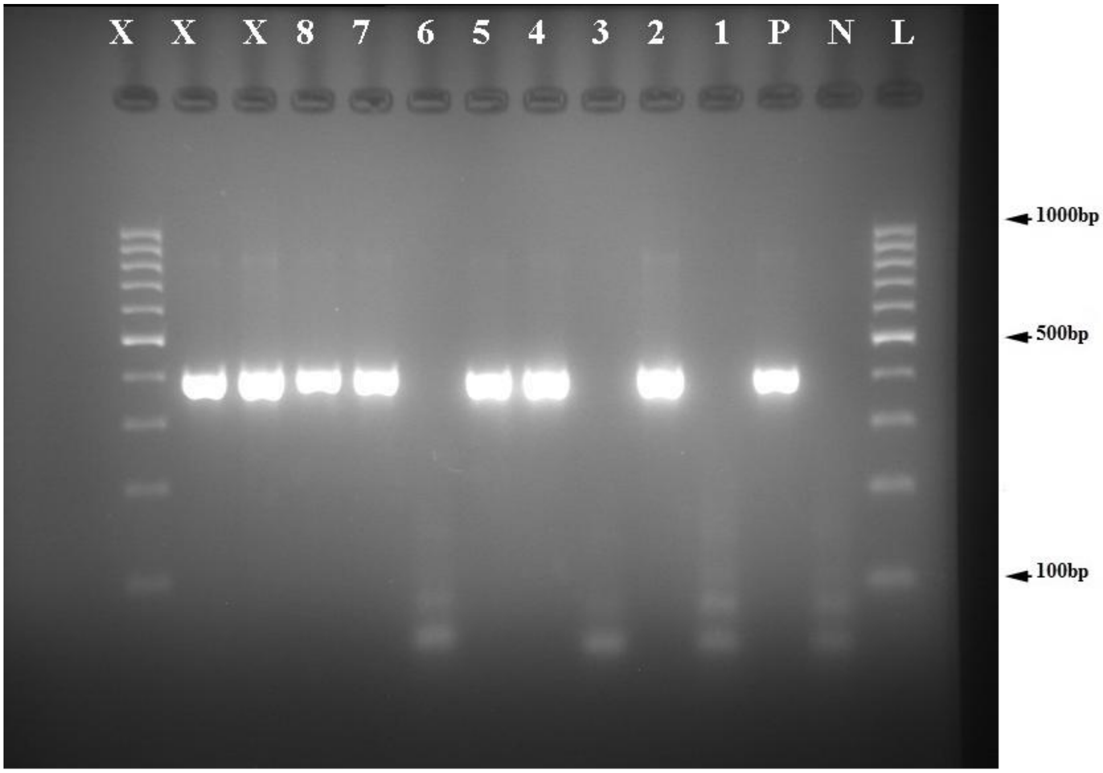

Fig 2. Electrophoresis of PCR products of S region of HBV genome extracted from serum samples of pregnant women on 2% agarose gel. L, 100-bp DNA ladder; N, negative control; P, positive control; 1, 2, 3, 4, 6, 7 and 9, amplified products (≈417 bp).

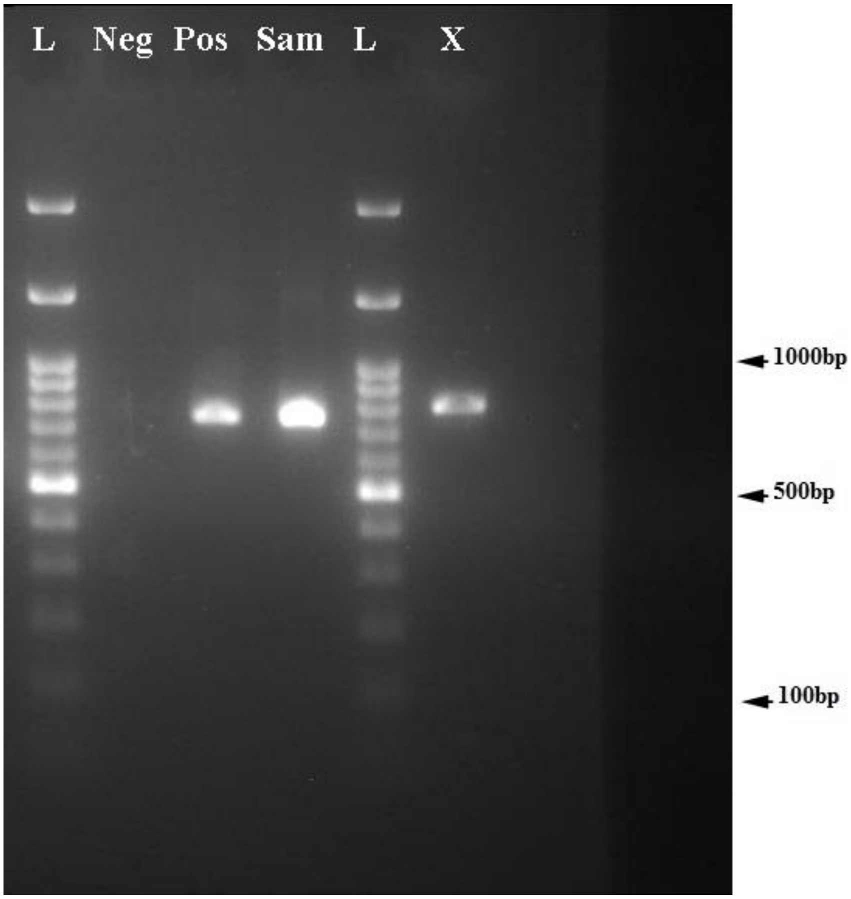

Fig 3. Electrophoresis of PCR products of X and pre-core regions of HBV genome extracted from serum samples of pregnant women on 2% agarose gel. L, 100-bp DNA ladder; Neg, negative control; Pos, positive control; Sam, amplified product (≈740 bp).
