# Supplementary material for: Prevalence, genotype distribution and mutations of hepatitis B virus and the associated risk factors among pregnant women residing in the northern shores of Persian Gulf, Iran
Source: PLoS One. 2022 Mar 10;17(3):e0265063. doi: 10.1371/journal.pone.0265063 (PMC8912131; doi:10.1371/journal.pone.0265063)
Supplement: Attachment — Submitted filename: PONE-D-21-32739_R1_reviewer.pdf [file pone.0265063.s010.pdf]

PLOS ONE

Prevalence, genotype distribution and mutations of hepatitis B virus infection and associated risk factors among pregnant women residing in the northern shores of Persian Gulf, Iran

--Manuscript Draft--

**Manuscript Number:**	PONE-D-21-32739R1
**Article Type:**	Research Article
**Full Title:**	Prevalence, genotype distribution and mutations of hepatitis B virus infection and associated risk factors among pregnant women residing in the northern shores of Persian Gulf, Iran
**Short Title:**	Hepatitis B virus infection among pregnant women in Iran
**Corresponding Author:**	Fatemeh Farshadpour, Ph.D<br>Bushehr University of Medical Sciences<br>Bushehr, IRAN, ISLAMIC REPUBLIC OF
**Keywords:**	Hepatitis B virus; Pregnancy; Risk factor; Prevalence; Genotype; Mutation; Iran
**Abstract:**	Background<br><br>Considering perinatal transmission and the high rate of chronic hepatitis B virus (HBV) infection in infants, diagnosis of HBV infection during pregnancy and timely interventions are of great importance. Therefore, this study was performed to investigate the prevalence and genotype distribution of HBV infection and associated risk factors among pregnant women in the northern shores of the Persian Gulf, South of Iran.<br><br>Methods<br><br>Serum samples of 1425 pregnant women were tested for the presence of HBsAg and HBcAb by ELISA assay (HBsAg one – Version ULTRA and HBc Ab ELISA kits, DIA.PRO, Milan, Italy). The seropositive samples were tested for the presence of HBV DNA by nested PCR, targeting S, X, pre-core (pre-C) and basal core promoter (BCP) regions of the HBV genome. The amplified fragments were sequenced by Sanger dideoxy sequencing technology to evaluate the genotype distribution and ~~mutations~~ of HBV ~~infection by~~ using the MEGA 7 software. Results: Of 1425 participants, 15 pregnant women (1.05%, 95% CI: 0.64%-1.73%) were positive for HBsAg, 41 women (2.88%, 95% CI: 2.10%-3.88%) were positive for HBcAb,and 5 women (0.35%, 95% CI: 0.15% –0.82%) had HBV viremia with genotype D, sub-genotype D3 and subtype ayw2. One of the viremic samples was positive for HBcAb but negative for HBsAg, which is indicative of an occult HBV infection. HBsAg seroprevalence was higher among pregnant women aged 20 to 29 years, women in the third trimester of pregnancy, residents of Khormuj city, Afghan immigrants, illiterate women, and pregnant women with the history of tattoo and HBV vaccination. The highest rate of HBcAb seroprevalence was observed in residents of Borazjan city, Turk ethnicity, the age group >39 years, and those women with more parities and a history of abortion. Nevertheless, HBV seroprevalence among pregnant women was not statistically associated with these variables. In contrast, HBcAb seropositivity was significantly associated with the history of tattoo (~~–~~P = 0.018). According to mutation~~s~~ analyses, seven amino acid substitutions in the HBsAg, one point mutation in the pre-C region, and five point~~s~~ mutations in the BCP region were detected. Besides, the BCP mutations caused amino acid substitutions in the X protein. Conclusion: These results indicate a relatively low prevalence of HBV infection among pregnant women in the South of Iran, while tattooing is a risk factor for exposure to HBV infection. Moreover, all of the HBV-positive pregnant women were asymptomatic and unaware of their infection. Therefore, routine screening for HBV markers during pregnancy, appropriate treatment of HBV-infected women, and HBV vaccination are recommended to decrease mother-to-child transmission of HBV.
**Order of Authors:**	Reza Taherkhani, Ph.D

Powered by Editorial Manager® and ProduXion Manager® from Aries Systems Corporation

	Fatemeh Farshadpour, Ph.D
**Response to Reviewers:**	ANSWERING REVIEWERS<br>December 15, 2021<br><br>Dear Editor-in-Chief of<br>PLOS ONE<br><br>We would greatly appreciate for your consideration of the publication of our manuscript entitled "Prevalence, genotype distribution and mutations of hepatitis B virus infection and associated risk factors among pregnant women resident in the northern shores of Persian Gulf, Iran" in the PLOS ONE.<br><br>Ms. No. PONE-D-21-32739<br><br>Title: Prevalence, genotype distribution and mutations of hepatitis B virus infection and associated risk factors among pregnant women resident in the northern shores of Persian Gulf, Iran<br><br>Name of Journal: PLOS ONE<br><br>Revision has been made according to the suggestions of the reviewers and the editor.<br><br>We greatly appreciated the reviewers' comments. The point-to-point responses to comments were shown as follows:<br><br><br>Journal Requirements:<br>When submitting your revision, we need you to address these additional requirements.<br>1. Please ensure that your manuscript meets PLOS ONE's style requirements, including those for file naming. The PLOS ONE style templates can be found at<br>https://journals.plos.org/plosone/s/file?id=wjVg/PLOSOne_formatting_sample_main_body.pdf<br>and<br>https://journals.plos.org/plosone/s/file?id=ba62/PLOSOne_formatting_sample_title_authors_affiliations.pdf<br>Response: The PLOS ONE's style requirements were applied.<br><br>2. Thank you for stating the following in the Acknowledgments Section of your manuscript:<br>"The authors would like to acknowledge grant number supported by the Deputy Research and 393 Affairs of the Bushehr University of Medical Sciences, Bushehr, Iran."<br>We note that you have provided additional information within the Acknowledgements Section that is not currently declared in your Funding Statement. Please note that funding information should not appear in the Acknowledgments section or other areas of your manuscript. We will only publish funding information present in the Funding Statement section of the online submission form.<br>Please remove any funding-related text from the manuscript and let us know how you would like to update your Funding Statement. Currently, your Funding Statement reads as follows:<br>"This study was funded by Bushehr University of Medical Sciences with grant number 357.<br>The funders had no role in study design, data collection and analysis, decision to publish, or preparation of the manuscript."<br>Please include your amended statements within your cover letter; we will change the online submission form on your behalf.<br>Response: The funding-related texts were removed from the Methods and the Acknowledgment sections of the manuscript. The funding information present in the Funding Statement section of the online submission form is correct.<br><br>3. PLOS ONE now requires that authors provide the original uncropped and unadjusted images underlying all blot or gel results reported in a submission's figures or Supporting Information files. This policy and the journal's other requirements for blot/gel reporting and figure preparation are described in detail at

https://journals.plos.org/plosone/s/figures#loc-blot-and-gel-reporting-requirements and https://journals.plos.org/plosone/s/figures#loc-preparing-figures-from-image-files. When you submit your revised manuscript, please ensure that your figures adhere fully to these guidelines and provide the original underlying images for all blot or gel data reported in your submission. See the following link for instructions on providing the original image data: https://journals.plos.org/plosone/s/figures#loc-original-images-for-blots-and-gels.
In your cover letter, please note whether your blot/gel image data are in Supporting Information or posted at a public data repository, provide the repository URL if relevant, and provide specific details as to which raw blot/gel images, if any, are not available. Email us at plosone@plos.org if you have any questions.
Response: The original uncropped and unadjusted images underlying gel results were provided. In the original uncropped figure 1, seven positive samples are shown. Since, two positive samples (positive samples number 4 and 5) were loaded again in the wells number 12 and 13. The reason was we did not want to have empty wells, we tried to load all of the wells.

4. Your ethics statement should only appear in the Methods section of your manuscript. If your ethics statement is written in any section besides the Methods, please delete it from any other section.
Response: The ethics statement was removed from end of the manuscript.

5. Please include captions for your Supporting Information files at the end of your manuscript, and update any in-text citations to match accordingly. Please see our Supporting Information guidelines for more information: http://journals.plos.org/plosone/s/supporting-information.
Response: Captions for the Supporting Information files were included at the end of the manuscript.

Additional Editor Comments:
The manuscript in the present form demands a “major revision” before it can be published. Therefore, we invite you to submit a revised version of the manuscript that addresses the points raised during the review process.
Response: We greatly appreciate you taking the time to consider our manuscript while providing us constructive comments. Moreover, thank you very much for giving us the opportunity to answer the reviewers’ comments. We are really grateful for this opportunity.

1. Please ensure that your manuscript meets PLOS ONE's style requirements, including those for headings. The PLOS ONE style templates can be found at https://journals.plos.org/plosone/s/file?id=wjVg/PLOSOne_formatting_sample_main_body.pdf
Response: The PLOS ONE's style requirements were applied.

2. Quality of written English is not suitable for publication unless extensively edited eg. The word “uneducated” should be replaced by “Illiterate”.
Response: The manuscript was edited for proper English language, grammar, punctuation, and spelling by our team at Bushehr University of Medical Sciences.

3. The limitations of the study are not sufficiently discussed.
Response: The limitations of the study were discussed in more details and highlighted.

4. Abstract needs to be re-written to reflect the study more correctly eg. Amplification of X gene is not included.
Response: The abstract was re-written.

Minor comments:
Change laboratory diagnosis sub-heading to laboratory methods.
Response: The change was done.

The author should consider using subheadings in the "laboratory diagnosis” section for easier reading.
Suggestion: HBV serological testing, PCR amplification and sequencing, phylogenetic and mutational analysis or sequence analysis.

Powered by Editorial Manager® and ProduXion Manager® from Aries Systems Corporation

Response: The suggestions were applied.

Include the full name of the ELISA kit used.
Response: The full name of the ELISA kits was included and highlighted with green color.

Table 1- Include degrees (℃) in the annealing temperature column.
Response: The degrees (℃) in the annealing temperature column was included.

The author indicate that the sequences has been submitted to GenBank, but no accession numbers provided.
Response: The accession numbers were provided and highlighted with yellow color in the result section and the legends of Fig3 and S1-S3 Figs.
The sequences have been submitted to GenBank and the accession numbers were provided via email, but they have not been released yet.

The study relied on nucleotide sequence analyses. However, there is no mention of sequencing protocol/approach/platform used in generating the nucleotide sequences in the methods section, only referenced a company that looks like it provides service.
Response: The Sanger dideoxy sequencing technology was included in the method section.

Table 2 and 3 should be moved to supplementary.
Response: The table 2 and 3 were moved to supplementary.

One of the city is stated as Khormoj in Table 2 and throughout it is stated as Khormuj.
Response: The spelling was corrected in the table 2.

Phylogenetic and mutational analysis (Figure 3 and Table 4) focused on 3 samples yet they detected HBV DNA in 5 samples. It is not clear whether the other 2 samples could not be sequenced or why they were left out of the analysis.
Response: Unfortunately, the other 2 samples had low-quality sequencing results, so that it was impossible to include them in the phylogenetic and mutational analysis.

Figure 3: Only indicate bootstrap values >70% which is considered moderate confidence.
Response: The phylogenetic tree was constructed by the neighbor-joining method with the Bootstrap test of phylogeny (substitutional model: maximum composite likelihood) in Molecular Evolutionary Genetics Analysis (MEGA) program version 7. Bootstrap resampling strategy and reconstruction were carried out 1,000 times to confirm the reliability of the phylogenetic tree.

Reviewer's Responses to Questions
Comments to the Author

1. Is the manuscript technically sound, and do the data support the conclusions?

The manuscript must describe a technically sound piece of scientific research with data that supports the conclusions. Experiments must have been conducted rigorously, with appropriate controls, replication, and sample sizes. The conclusions must be drawn appropriately based on the data presented.
Reviewer #1: No
Reviewer #2: Yes
Reviewer #3: Yes
Reviewer #4: Yes

---

2. Has the statistical analysis been performed appropriately and rigorously?
Reviewer #1: No
Reviewer #2: Yes
Reviewer #3: Yes
Reviewer #4: Yes

---

3. Have the authors made all data underlying the findings in their manuscript fully available?

The PLOS Data policy requires authors to make all data underlying the findings described in their manuscript fully available without restriction, with rare exception (please refer to the Data Availability Statement in the manuscript PDF file). The data should be provided as part of the manuscript or its supporting information, or deposited to a public repository. For example, in addition to summary statistics, the data points behind means, medians and variance measures should be available. If there are restrictions on publicly sharing data—e.g. participant privacy or use of data from a third party—those must be specified.
Reviewer #1: Yes
Reviewer #2: Yes
Reviewer #3: Yes
Reviewer #4: Yes

____
4. Is the manuscript presented in an intelligible fashion and written in standard English?

PLOS ONE does not copyedit accepted manuscripts, so the language in submitted articles must be clear, correct, and unambiguous. Any typographical or grammatical errors should be corrected at revision, so please note any specific errors here.
Reviewer #1: No
Reviewer #2: Yes
Reviewer #3: Yes
Reviewer #4: Yes

____
5. Review Comments to the Author
Please use the space provided to explain your answers to the questions above. You may also include additional comments for the author, including concerns about dual publication, research ethics, or publication ethics. (Please upload your review as an attachment if it exceeds 20,000 characters)

Reviewer #1: The author used unacceptable language (e.g. uneducated women) which also feels derogatory. The manuscript used unnecessary statistics, while its main research was HBV infection (HBsAg or HBV DNA) or exposure as determined by HBcAb. Only 15 pregnant women were HBsAg positive but the manuscript made a lot of unnecessary and unacceptable statistical analysis (smoking, Education to mention few).
Response: Thank you very much for your review.

-The manuscript was edited for proper English language, grammar, punctuation, and spelling.

-These variables were included in the study in order to find the possible risk factors of HBV infection among pregnant women.
Regarding smoking: In addition to cigarette, some women in the South of Iran use hookah for smoking and they share the hookah in group. We thought that there might be a chance of HBV transmission through saliva by using hookah.

Regarding education: The education level has an indirect effect on the prevalence of HBV. The educated women have more information about the transmission routes of HBV.

Regarding the month of sampling: We assumed that there might be a seasonal or monthly pattern regarding the seroprevalence of HBV infection among pregnant women in this region.
According to those studies on the seasonality of hepatitis, we wanted to investigate the seasonal pattern in this study too.

Reviewer #2: This was a well-designed study that showed the prevalence and molecular characterization of HBV in pregnant woman. The literature review clearly stated the problem and the aim of the study. The methodology used was standard for

Powered by Editorial Manager® and ProduXion Manager® from Aries Systems Corporation

this type of study.
Response: Thank you very much for your review and the positive comments.

In the results section, there is a comment on the occult status in Line 193, it is not shown what the viral load of this sample is in order to make such a statement. I suggest the author includes viral load of this sample.
Response: Occult HBV infection is described by the presence of HBV DNA in the absence of detectable HBsAg in serum or plasma. This sample, despite being positive for HBV DNA, was negative for HBsAg, which is indicative of an occult HBV infection.

-We did not measure HBV viral loads since the nested PCR assay used in this study is a qualitative method and is not able to determine the viral loads. This is a limitation of nested-PCR over quantitative PCR (qPCR). The limitations of nested PCR assay were included in the manuscript and highlighted with yellow color in the limitations section of the study.

-Those samples with very low virus levels become positive in the second round of nested PCR. This sample with occult HBV infection was identified in the second round of amplification, which is indicative of low HBV DNA levels.

- In one of our previous research projects (unpublished data), the viral load of one of the HBV DNA positive serum samples was measured by real time PCR, and the serum sample was serial diluted. The diluted sample with 25 HBV DNA copies/ml (5 HBV DNA copies/200 µL) became positive in the second round of nested PCR, while it was negative in the first round of PCR.

In the discussion, in Line 255, the author states that the HBV prevalence of 1.05% was lower than 1.18%, these are similar especially if rounded off.
Response: We cannot round off the numbers here. Because regarding the prevalence rate, even a small decimal is important. We used “slightly” in the sentence.

In line 258, Risk of mother to child transmission should be discussed more here, they did not show what the risk of undiagnosed HBV in pregnancy would be, especially in those infected in the third trimester.
Response: The mentioned issue was included and highlighted with yellow color.

Line 268, This recommendation is not clear. Do they mean that they will test each and every women in the South of Iran area? this is not cost effective, rather screen all women who come in for their first visit at the ANC and vaccinate the baby at birth. Or treat the infection if it becomes chronic.
Response: We agree with the dear reviewer. This sentence was revised and highlighted with green color.

Line 319, Cannot make this conclusion as this was not part of the questions asked in the questionnaire as shown in the information on Table 2 and 3. You only have vaccination data for 14 participants out of over 1000. This is being very presumptuous and this conclusion needs to be made with caution.
Response: We agree with the dear reviewer. Therefore, the sentence was revised and highlighted with green color.

-A significant percentage of the pregnant women in this study were not vaccinated according to self-declaration during the interview. Only 22.0% of the participants were vaccinated, 22.0% were not vaccinated and 55.9% of the participants did not respond to the questions regarding history of HBV vaccination during interview (Unknown group). These participants did not know anything about HBV vaccination. Considering the compulsory vaccination of infants since 1993 and teenagers since 2006 in Iran, individuals above 30 years were not the target group of national vaccination program. Since a high number of the “Unknown group” were above 30 years, we think that a significant percentage of the pregnant women in this study were not vaccinated. These data are shown in the tables 2 and 3.

-According to the aim of the study and the available funding, the presence of hepatitis B surface antigen (HBsAg), hepatitis B core antibody (HBcAb) and HBV viremia was investigated in serum samples of the participants. Unfortunately, we did not assess

Powered by Editorial Manager® and ProduXion Manager® from Aries Systems Corporation

antibody to hepatitis B surface antigen (anti-HBsAg) among the study population due to lack of budget. This issue was mentioned as the limitation of the study, and it is suggested to study the effectiveness of the immunization policy in the pregnant population of this region.

Line 326, this statement contradicts what the authors had said in Line 318-320 on the ineffectiveness of the HBV vaccination program.
Response: The previous statement has been revised.

-The previous statement was about the seroprevalence of HBV in the age group 20-29 years old. This statement is about the pregnant women under 20 years old.

Line 331, What was the viral load of this sample? it was also not mentioned in the results section.
Response: We did not measure HBV viral loads since the nested PCR assay used in this study is a qualitative method and is not able to determine the viral loads. This a limitation of nested-PCR over qPCR. The limitations of nested PCR assay were included in the manuscript.
Those samples with very low virus levels become positive in the second round of nested PCR. This sample with occult HBV infection was identified in the second round of amplification, which is indicative of low HBV DNA levels.

-In one of our previous research projects (unpublished data), the viral load of one of the HBV DNA positive serum samples was measured by real time PCR, and the serum sample was serial diluted.
The diluted serum sample with 25 HBV DNA copies/ml (5 HBV DNA copies/200 μL) became positive in the second round of nested PCR, while it was negative in the first round of PCR.

Line 339-340, show the importance of this lack of HBeAg during pregnancy, this has not been discussed fully here.
Response: No, this sentence does not show the importance of the lack of HBeAg during pregnancy. This shows the relationship between the expression of HBeAg and immune responses to HBV infection.
It shows that the absence of HBeAg may result in evasion of immune clearance as HBeAg is a target antigen for immune recognition.
The sample with G1896A mutation was HBeAg negative. The G1896A mutation causes the conversion of TGGTAG (Trp Stop codon) at codon 28 of the pre-C gene and the suppression of HBeAg expression. This HBeAg negative sample is at the risk of liver disease progression.
In fact, it wants to show that the mutation analysis of HBV infection can be useful in predicting the disease progression in HBV-infected patients.

The Conclusion on Line 376 differs from the conclusion on the abstract. The one in the abstract lines up with what the authors set out to do, while the one in Line 376 is a repetition of what the results stated.
Response: The conclusion was revised and highlighted.

Reviewer #3: Title: suggestion, change “resident” to “residing”
Response: Thank you very much for your review and the constructive comments.

- The suggestion was applied and highlighted with yellow color.

Abstract: Page 2, line 32 please mention the name of the ELISA kit used, company and the sentence that follows should read tested for the presence of HBV DNA delete “detection”.
Response: The full name of the ELISA kits was included.

Page 2, line 34 the software or platforms used to analyze the mutations are not mentioned.
Response: The software was included.

Page 2, line 43, the sentence talking about tattooing being a risk factor should come in the conclusion. Or the sentence can either be stated as significantly associated or as risk factor not two of them.
Response: The abstract was revised.

Page 2, line 36, did the HBcAb positive women have HBsAg or were they only positive for HBcAb? This is because in your results you state that 2.7% of HBcAb positive were also positive for HBV DNA.
Response: Of 41 HBcAb seropositive women, 4 women were positive for HBsAg (HBsAg positive and HBcAb positive), and 37 women were negative for HBsAg (HBsAg negative and HBcAb positive).

Overall, 26.7% of HBsAg seropositive pregnant women and 2.7% of HBcAb seropositive women had HBV viremia with genotype D, sub-genotype D3, and subtype ayw2:

2.7% (1 case of 37 HBsAg negative and HBcAb positive cases)
26.7% (4 cases of 15 HBsAg positive cases)

Page ,2 line 46 Please indicate how many had which genotype.
Response: All of the HBV DNA positive samples had HBV viremia with genotype D, sub-genotype D3 and subtype ayw2.

Page 2, line 49, in the methods the authors mention the pre-core regions however in the results they mention pre-core and basal core regions.
Response: The target genes were included.

Page 2, line 48, it is important to note that the mutations were detected in the same sample deemed as an occult, it is not clear in the statement.
Response: The mutations were not specific to one sample. The mutations were detected in the S, X, BCP and pre-C regions of the HBV genome isolated from pregnant women and were not specific to the occult HBV sample.
According to mutations analyses, seven amino acid substitutions in the HBsAg, one point mutation in the pre-C region and five points mutations in the basal core promoter (BCP) region were detected. Besides, the BCP mutations caused amino acid substitutions in the X protein.

Page 2, line 50 to 53, the authors are not concluding on their findings but are making a general remark. First it should be clear if HBV is endemic or not in this region and if perinatal transmission is a problem or not. In their results the authors found a very low prevalence, they should say something about it in their conclusion.
Response: The conclusion was revised.

Page 4, line 95, please add the information about the target genes missing in the abstract.
Response: The target genes were included.

Page 7, line 167, means 1.6% of the vaccinated women had chronic or acute infection.
Response: Yes, it means 1.6% of the vaccinated women were HBsAg seropositive, or the seroprevalence of HBsAg among vaccinated women was 1.6%.

Page 8, line 177, the authors did not mention, any liver enzyme tests performed however they are mentioning them in their results.
Response: Levels of the liver enzymes were obtained from the records of pregnant women at the public health centers. The pregnant women attend these public health centers for periodical checkups.

Page 8, line 177-179, the tables should also be referred to in the text.
Response: The tables were referred to in the text.

Page 11, line 190, a suggestion to delete “evaluation” to “testing”
Response: The suggestion was applied.

Powered by Editorial Manager® and ProduXion Manager® from Aries Systems Corporation

Page 11, line 190 the authors should stick to one decimal after the comma throughout the document for consistency.
Response: We cannot round off the numbers here. Because regarding the prevalence rate, even a small decimal is important.

Page 14, line 258 to 263 the sentence is too long.
Response: The long sentence was rephrased.

Why did the authors not check the women vaccination status? Why did the authors not test for Anti-HBs but the authors are making a statement in the discussion that majority of women are not vaccinated in this region? Do the authors mean to tell us that there is no HBV routine testing in pregnant women in this region?
Response: A significant percentage of the pregnant women in this study were not vaccinated according to self-declaration during the interview. Only 22.0% of the participants were vaccinated, 22.0% were not vaccinated and 55.9% of the participants did not respond to the questions regarding history of HBV vaccination during interview (Unknown group). These participants did not know anything about HBV vaccination. Considering the compulsory vaccination of infants since 1993 and teenagers since 2006 in Iran, individuals above 30 years were not the target group of national vaccination program. Since a high number of the “Unknown group” were above 30 years, we think that a significant percentage of the pregnant women in this study were not vaccinated. These data are shown in the tables 2 and 3.

-According to the aim of the study and the available funding, the presence of hepatitis B surface antigen (HBsAg), hepatitis B core antibody (HBcAb) and HBV viremia was investigated in serum samples of the participants. Unfortunately, we did not assess antibody to hepatitis B surface antigen (anti-HBsAg) among the study population due to lack of budget. However, we agree with the dear reviewer that the lack of awareness of the level of immunity among the study population can influence interpretation of the study findings. This issue was mentioned as the limitation of the study, and it is suggested to study the effectiveness of the immunization policy in the pregnant population of this region.

-Yes, there is no guideline or national policy on HBV screening during pregnancy. In fact, this study was performed to convince the health care providers to include HBV screening in the routine screening of pregnant women in Iran. Currently, pregnant women are only screened for rubella and CMV in Iran.

Page 16, line 319-320 the authors cannot claim that the HBV vaccination programs are not effective while they did not report if these people had received the vaccine or not and check why if they did not received the vaccine?
Response: We agree with the dear reviewer. Therefore, the sentence was revised and highlighted with green color.
- This issue was mentioned as the limitation of the study, and it is suggested to study the effectiveness of the immunization policy in the pregnant population of this region.

Page 17, line 323, how did the authors arrive at a conclusion that the HBV management during pregnancy is facing challenges?
Response: Because although there are HBV vaccine and oral antivirals and screening tools for the prevention, treatment and diagnosis of HBV infection, the status of immunity and prevalence of HBV infection among pregnant population in Iran is unknown. Since, there is no guideline or national policy on HBV screening during pregnancy. We need such kind of studies among pregnant women to convince the health care providers to include HBV screening in the management program of pregnant women in Iran.

Page 17, line 332 please rephrase the sentence.
Response: The sentence was rephrased and highlighted with yellow color.

This sample was found to be positive in the second round of PCR; this statement is irrelevant in the discussion.
Response: The sentence was removed.

Powered by Editorial Manager® and ProduXion Manager® from Aries Systems Corporation

Page 17, line 340, the authors state that one sample was negative for HBeAg however this was not mentioned in the methods that was tested in any sample.
Response: Unfortunately, the presence of HBeAg was not investigated in the serum samples of all participants due to the lack of budget (the available funding could not cover the expenses to test all of the samples). Only the seropositive samples were tested for the presence of HBeAg. Therefore, detection of HBeAg was not mentioned in the methods section.

Page 19, line 374, the statement is not clear.
Response: Due to the cross-sectional design of the study, we could not determine the possible effects of hepatitis B on pregnancy outcomes. Therefore, prospective or longitudinal studies are required to determine the possible effects of hepatitis B on pregnancy outcomes among pregnant women in the South of Iran.
Prospective studies are required to complete our study regarding the possible effects of hepatitis B on pregnancy outcomes.

General comments
The authors should try to keep their sentences short.
Response: The long sentences were rephrased.

The pictures are not clear; they are not of good quality.
Response: The pictures with high quality were provided.

The authors in their conclusion make no mention of the risk factors associated with HBV infection in their study.
Response: The conclusion was revised.

The authors can summarize the results section in the abstract.
Response: The abstract was revised.

Information on vaccination in the study area is not mentioned in the introduction, the vaccination coverage, when did the vaccination starts, which age group is targeted, the HBV testing program for pregnant women in this area is also missing in the introduction. These informs the reader about the situation in the study area and supports why study was conducted.
Response: The information on vaccination is mentioned in the introduction and highlighted with yellow color.
-There is no national policy on HBV screening during pregnancy in Iran. In fact, this study was performed to convince the health care providers to include HBV screening in the routine screening of pregnant women in Iran. Currently, pregnant women are only screened for rubella and CMV in Iran.

There is no made mention of how the sample size was calculated only how the study sites were identified.
Response: This is a cross-sectional study that was performed from January 2018 to June 2019.
During the study period, all pregnant women who agreed to participate and gave written informed consent to use their samples for HBV detection and their data for analysis were included consecutively in this study.

-This has been mentioned in the methods section: “From January 2018 to June 2019, all the pregnant women attending these public health centers for routine visits were included consecutively in this study. The pregnant women gave written informed consent to participate in the study and use their serum samples for HBV detection.”

Reviewer #4: While the findings of this study will fill a critical knowledge gap and contribute to informing interventions aimed at preventing HBV mother-to-child transmission in Iran, there is need for further clarity in the reporting of the methodology followed and results obtained in order to improve the quality of the manuscript. In addition, the limitations of the study are not explicitly addressed in the manuscript.
Response: Thank you very much for your review and the positive comments. The mentioned issues were included and highlighted.

Reviewer’s Comments

Given the increased lifetime risk for potentially fatal chronic liver disease following perinatally acquired hepatitis B virus (HBV) infection, this study sought to characterize the burden of HBV infection among pregnant women living in the South of Iran. While the findings of this study will fill a critical knowledge gap and contribute to informing interventions aimed at preventing HBV mother-to-child transmission in Iran, there is need for further clarity in the reporting of the methodology followed and results obtained in order to improve the quality of the manuscript.
Response: Thank you very much for your review and the positive comments.

Major Revisions:
The introduction section does not address universal hepatitis B vaccination globally and in Iran, and how this has influenced the burden of disease over time. In addition, the authors do not address the availability, accessibility and utilization of maternal HBV screening and management programmes in Iran. These should be addressed to provide further clarity to the context of the study.
Response: The mentioned issue was included and highlighted with yellow color in the introduction section.
-Unfortunately, there is no guideline or national policy on HBV screening during pregnancy. In fact, this study was performed to convince the health care providers to include HBV screening in the routine screening of pregnant women in Iran. Currently, pregnant women are only screened for rubella and CMV in Iran.

In the methods section of the manuscript, the authors adequately address how socio-demographic, and clinical data were collected from study participants. However, there is no information on how blood samples were collected for the study.
Response: The leftover serum samples of pregnant women who attend the public health centers for routine checkups were used for HBV detection.

The authors allude to the use of a questionnaire in collecting relevant participant data. For validation purposes, kindly indicate of this questionnaire was piloted. Was a previously published questionnaire used during this study (in which case it should be appropriately cited and referenced) or if a study specific one was developed, can a template be provided as part of supplementary material.
Response: The socio-demographic characteristics and pregnancy information were obtained by interviewing each pregnant woman using a questionnaire (a study specific one was developed). The questionnaire was translated to English and provided as supplementary.

The authors have not addressed why antibodies to the hepatitis B surface antigen (anti-HBs) was not tested among the study population. It will be interesting to understand from the introduction section how long Iran has had a universal hepatitis B vaccination programme and whether any of the younger participants should have been vaccinated in infancy. A lack of awareness of the level of immunity (either due to vaccination or recovery from a past infection) among the study population may influence interpretation of the study findings and should therefore be adequately addressed.
Response: According to the aim of the study and the available funding, the presence of hepatitis B surface antigen (HBsAg), hepatitis B core antibody (HBcAb) and HBV viremia was investigated in serum samples of the participants. Unfortunately, we did not assess antibody to hepatitis B surface antigen (anti-HBsAg) among the study population due to lack of budget. However, we agree with the dear reviewer that the lack of awareness of the level of immunity among the study population can influence interpretation of the study findings. This issue was mentioned as the limitation of the study, and it is suggested to study the effectiveness of the immunization policy in the pregnant population of this region.

The limitations of the study are not explicitly addressed in the manuscript.
Response: The limitations of the study were discussed in more details and highlighted.

Minor Revisions:
Line 61, page 3; “HBV-related liver diseases are responsible for approximately 8,00,000 deaths…” Consider revising the number format (800 000) for better clarity.
Response: The number format was revised.

Powered by Editorial Manager® and ProduXion Manager® from Aries Systems Corporation

Lines 116 – 117, page 5; "…using commercially available ELISA kits (DIA.PRO, Milan, Italy)." For better clarity and reliability, the authors should briefly provide further information (e.g., type of platform used, automated or manual, etc) about the ELISA used. In addition, did the authors follow the manufacturer's instructions or a published protocol (cite appropriately).
Response: The mentioned issues were included and highlighted with green color.

Line 118, page 5; "…distribution and mutations of HBV infection by two nested PCR assays, targeting S, X and pre-C…" Was this an in-house PCR assay or did the authors follow a previously published protocol – in which case this should be appropriately cited and referenced. Also, important to note is the fact that the authors have not addressed the limitations of the nested PCR assay used (vs qPCR) in the manuscript.
Response: We used a previously published protocol, and the referenced articles were cited in the table 1.
The strengths and limitations of nested PCR assay were included in the manuscript and highlighted.

Line 148, page 6; "…Logistic regression analysis was used to evaluate the risk factors…" Kindly clarify if univariate and multivariate logistic regression analyses were performed.
Response: Univariate and multivariate logistic regression analyses were performed using HBsAg and HBcAb seroprevalence as dependent variables and socio-demographic characteristics and qualitative variables (age, stage of gestation, number of pregnancies, smoking, place of residency, level of education, ethnicity, time of sampling, and history of blood transfusion, tattoo, abortion, dentistry, surgery and HBV vaccination), as independent variables.

Line 167, page 7; "…uneducated (2.4%) and had a history of tattoo (1.6%) and HBV vaccination (1.6%)." Kindly clarify in the manuscript whether participants' vaccination history was self-reported only or confirmed using clinical records. The limitations of relying on self-reported clinical history / patient recall, should be adequately addressed in the manuscript where appropriate.
Response: The participants' vaccination history was obtained according to self-declaration during the interview. This was reported in the method section and highlighted with green color.

-Unfortunately, we did not assess antibody to hepatitis B surface antigen (anti-HBsAg) among the study population due to lack of budget. This issue was mentioned as the limitation of the study and highlighted with green color, and it is suggested to study the effectiveness of the immunization policy in the pregnant population of this region.

Lines 176 – 177, page 8; "All of the HBV seropositive samples had normal levels of liver enzymes and were negative for HCV and HIV." In the methods section, the authors do not make mention of testing for liver enzymes, HIV or HCV co-infection. The authors should address how they came about these findings reported here, i.e., tested in this study, self-reported during questionnaire / interview, confirmed from reliable clinical records of participants.
Response: Levels of the liver enzymes were obtained from the records of pregnant women at the public health centers. The pregnant women attend these public health centers for periodical checkups. This was mentioned in the method section and highlighted with green color.

- The HBV seropositive pregnant women were tested for HIV and HCV coinfections by ELISA. This issue was included in the method section and highlighted with yellow color.

Lines 257 – 258, page 14; "These infected but asymptomatic pregnant women may remain undiagnosed over time." As a matter of translating research to practice, can the authors comment on the direct benefits of the study findings to the participants in this case, i.e., were hepatitis B results reported to clinicians managing participants in order to inform timely interventions?
Response: The results of this study were informed to the public health centers and the Deputy Research and Affairs of the Bushehr University of Medical Sciences. Surely,

Powered by Editorial Manager® and ProduXion Manager® from Aries Systems Corporation

	the results have been communicated to the infected pregnant women by the authorities in the public health centers. However, we are not sure about receiving treatment or care post-pregnancy by HBV positive women. Since, the follow-up of infected pregnant women was outside the scope of the study. This issue has been mentioned as a limitation of this cross-sectional study in the manuscript.<br><br>Lines 260 – 263, page 14; "Since maternal treatment with oral antivirals…HBV vaccination and immunoglobulin administration at birth…consequently, reduce the burden of chronic HBV infection in the community [3, 16]." The authors should expand on what the national policies and guidelines in relation to these key interventions are, with an indication of the current coverage / utilization rates of these services in the country.<br>Response: National policy and intervention services for HBV infected pregnant women are the same as reported in the manuscript:<br>1)Maternal treatment with oral antivirals such as tenofovir, telbivudine and lamivudine<br>2)HBV vaccination and immunoglobulin administration at birth<br><br>Lines 287 – 288, page 15; "…and the sensitivity and specificity of ELISA kits in different studies can also explain these variations." The authors should explicitly address the strengths and limitations of assays (nested PCR vs qPCR, not testing for anti-HBs or HBeAg, sensitivity and specificity of ELISA) used in this study compared to those in the aforementioned studies and how this may influence interpretation of the study findings.<br>Response: The strengths and limitations of nested PCR assay were included in the manuscript and highlighted (limitation section of the study).<br><br>- Sensitivity and specificity of the ELISA kits were included in the method section.<br><br>Lines 301 – 303, page 16; "In addition, HBcAb seropositivity was more prevalent in those samples collected in October..." It is unclear why the authors assessed the frequency of detection of HBV infection in relation to the months in which samples were collected. Were the authors expecting a seasonal pattern in relation to the prevalence of HBV infection? Have such seasonal patterns been demonstrated elsewhere and if so, what would the relevance of this be?<br>Response: We did not find a seasonal pattern regarding the seroprevalence of HBV infection among pregnant women in this region. However, according to those studies on the seasonality of hepatitis, we wanted to investigate the monthly or seasonal pattern in this study too.<br><br>Lines 309 – 310, page 16; "…a significant association was found between circumcision and HBsAg seropositivity." With regards to the study cited, could the authors clarify if this implies male circumcision among spouses / sexual partners of pregnant women found to be HBsAg seropositive?<br>Response: It is female circumcision, which is usually performed at home.<br><br>Lines 330 – 331, page 17; "…undetectable levels of HBsAg and low HBV DNA levels." Did the authors measure HBV viral loads?<br>Response: No, we did not measure HBV viral loads since the nested PCR assay used in this study is a qualitative method and is not able to determine the viral loads. This a limitation of nested-PCR over qPCR. The limitations of nested PCR assay were included in the manuscript.<br><br>-Those samples with very low virus levels become positive in the second round of nested PCR. This sample with occult HBV infection was identified in the second round of amplification, which is indicative of low HBV DNA levels.<br><br>-In one of our previous research projects (unpublished data), the viral load of one of the HBV DNA positive serum samples was measured by real time PCR, and the serum sample was serial diluted. The diluted serum sample with 25 HBV DNA copies/ml (5 HBV DNA copies/200 µL) became positive in the second round of nested PCR, while it was negative in the first round of PCR.<br><br>Line 340, page 17; "Notably, the sample with G1896A mutation was HBeAg negative." In the methods section the authors did not indicate serological testing for additional markers aside for HBsAg and anti-HBc (HBcAB). Could the authors address how they

Powered by Editorial Manager® and ProduXion Manager® from Aries Systems Corporation

	came about this finding in the methods section and why it was not reported as part of the results?<br>Response: Unfortunately, the presence of HBeAg was not investigated in the serum samples of all participants due to the lack of budget (the available funding could not cover the expenses to test all of the samples). Only the seropositive samples were tested for the presence of HBeAg. Therefore, detection of HBeAg was not mentioned in the methods section.<br><br>Lines 349 – 350, page 18; “The dominance of genotype D is the most important characteristic…in our region.” In addition to the stated predominant HBV genotype in the region, the authors should indicate the HBV genotype distribution in Iran as this has not been provided as part of the introduction section.<br>Response: The HBV genotype distribution in Iran was provided and highlighted with green color.<br><br>Sincerely yours,<br>Fatemeh Farshadpour, Ph.D,<br>Bushehr University of Medical Sciences 7514633341<br>Bushehr, Iran.<br>00989171712653<br>E-mail: f.farshadpour@bpums.ac.ir<br>f.farshadpour@yahoo.com
**Additional Information:**	
**Question**	**Response**
**Financial Disclosure**<br><br>Enter a financial disclosure statement that describes the sources of funding for the work included in this submission. Review the submission guidelines for detailed requirements. View published research articles from *PLOS ONE* for specific examples.<br><br>This statement is required for submission and **will appear in the published article** if the submission is accepted. Please make sure it is accurate.	This study was funded by Bushehr University of Medical Sciences with grant number 357.<br>The funders had no role in study design, data collection and analysis, decision to publish, or preparation of the manuscript.

**Unfunded studies**<br>Enter: *The author(s) received no specific funding for this work.*<br><br>**Funded studies**<br>Enter a statement with the following details:<br>• Initials of the authors who received each award<br>• Grant numbers awarded to each author<br>• The full name of each funder<br>• URL of each funder website<br>• Did the sponsors or funders play any role in the study design, data collection and analysis, decision to publish, or preparation of the manuscript?<br>• **NO** - Include this sentence at the end of your statement: *The funders had no role in study design, data collection and analysis, decision to publish, or preparation of the manuscript.*<br>• **YES** - Specify the role(s) played.<br><br>* typeset	
**Competing Interests**<br><br>Use the instructions below to enter a competing interest statement for this submission. On behalf of all authors, disclose any competing interests that could be perceived to bias this work—acknowledging all financial support and any other relevant financial or non-financial competing interests.<br><br>This statement is required for submission and **will appear in the published article** if the submission is accepted. Please make sure it is accurate and that any funding sources listed in your Funding Information later in the submission form are also declared in your Financial Disclosure statement.<br><br>View published research articles from *PLOS ONE* for specific examples.	The authors have declared that no competing interests exist.

Powered by Editorial Manager® and ProduXion Manager® from Aries Systems Corporation

**NO authors have competing interests**<br><br>Enter: *The authors have declared that no competing interests exist.*<br><br>**Authors with competing interests**<br><br>Enter competing interest details beginning with this statement:<br><br>*I have read the journal's policy and the authors of this manuscript have the following competing interests: [insert competing interests here]*<br><br>* typeset	
**Ethics Statement**<br><br>Enter an ethics statement for this submission. This statement is required if the study involved:<br><br>• Human participants<br>• Human specimens or tissue<br>• Vertebrate animals or cephalopods<br>• Vertebrate embryos or tissues<br>• Field research<br><br>Write "N/A" if the submission does not require an ethics statement.<br><br>General guidance is provided below. Consult the submission guidelines for detailed instructions. **Make sure that all information entered here is included in the Methods section of the manuscript.**	This study was approved by the Ethical Committee of Bushehr University of Medical Sciences with reference number IR.BPUMS.REC.1396.111.

Powered by Editorial Manager® and ProduXion Manager® from Aries Systems Corporation

**Format for specific study types**<br><br>**Human Subject Research (involving human participants and/or tissue)**<br>• Give the name of the institutional review board or ethics committee that approved the study<br>• Include the approval number and/or a statement indicating approval of this research<br>• Indicate the form of consent obtained (written/oral) or the reason that consent was not obtained (e.g. the data were analyzed anonymously)<br><br>**Animal Research (involving vertebrate animals, embryos or tissues)**<br>• Provide the name of the Institutional Animal Care and Use Committee (IACUC) or other relevant ethics board that reviewed the study protocol, and indicate whether they approved this research or granted a formal waiver of ethical approval<br>• Include an approval number if one was obtained<br>• If the study involved *non-human primates*, add *additional details* about animal welfare and steps taken to ameliorate suffering<br>• If anesthesia, euthanasia, or any kind of animal sacrifice is part of the study, include briefly which substances and/or methods were applied<br><br>**Field Research**<br><br>Include the following details if this study involves the collection of plant, animal, or other materials from a natural setting:<br>• Field permit number<br>• Name of the institution or relevant body that granted permission	
**Data Availability**<br><br>Authors are required to make all data underlying the findings described fully available, without restriction, and from the time of publication. PLOS allows rare exceptions to address legal and ethical concerns. See the PLOS Data Policy and FAQ for detailed information.	Yes - all data are fully available without restriction

Powered by Editorial Manager® and ProduXion Manager® from Aries Systems Corporation

A Data Availability Statement describing where the data can be found is required at submission. Your answers to this question constitute the Data Availability Statement and **will be published in the article**, if accepted.<br><br>**Important:** Stating 'data available on request from the author' is not sufficient. If your data are only available upon request, select 'No' for the first question and explain your exceptional situation in the text box.<br><br>Do the authors confirm that all data underlying the findings described in their manuscript are fully available without restriction?	
**Describe where the data may be found in full sentences. If you are copying our sample text, replace any instances of XXX with the appropriate details.**<br><br>• If the data are **held or will be held in a public repository**, include URLs, accession numbers or DOIs. If this information will only be available after acceptance, indicate this by ticking the box below. For example: *All XXX files are available from the XXX database (accession number(s) XXX, XXX.)*.<br>• If the data are all contained **within the manuscript and/or Supporting Information files**, enter the following: *All relevant data are within the manuscript and its Supporting Information files.*<br>• If neither of these applies but you are able to provide **details of access elsewhere**, with or without limitations, please do so. For example:<br><br>*Data cannot be shared publicly because of [XXX]. Data are available from the XXX Institutional Data Access / Ethics Committee (contact via XXX) for researchers who meet the criteria for access to confidential data.*<br><br>*The data underlying the results presented in the study are available from (include the name of the third party*	All relevant data are within the paper.

and contact information or URL).<br>• This text is appropriate if the data are owned by a third party and authors do not have permission to share the data.<br><br>* typeset	
Additional data availability information:	

Powered by Editorial Manager® and ProduXion Manager® from Aries Systems Corporation

Manuscript

Click here to access/download;Manuscript;Revised Manuscript.doc

Prevalence, genotype distribution and mutations of hepatitis B virus infection and associated risk factors among pregnant women residing in the northern shores of Persian Gulf, Iran

Reza Taherkhani and Fatemeh Farshadpour*

Department of Virology, School of Medicine, Bushehr University of Medical Sciences, Bushehr, Iran

***Corresponding author**

Email: f.farshadpour@bpums.ac.ir (FF)

Reza Taherkhani and Fatemeh Farshadpour contributed equally to this work.

Abstract

Background: Considering perinatal transmission and the high rate of chronic hepatitis B virus (HBV) infection in infants, diagnosis of HBV infection during pregnancy and timely interventions are of great importance. Therefore, this study was performed to investigate the prevalence and genotype distribution of HBV infection and associated risk factors among pregnant women in the northern shores of the Persian Gulf, South of Iran.

Methods: Serum samples of 1425 pregnant women were tested for the presence of HBsAg and HBcAb by ELISA assay (HBsAg one – Version ULTRA and HBc Ab ELISA kits, DIA.PRO, Milan, Italy). The seropositive samples were tested for the presence of HBV DNA by nested PCR, targeting S, X, pre-core (pre-C) and basal core promoter (BCP) regions of the HBV genome. The amplified fragments were sequenced by Sanger dideoxy sequencing technology to evaluate the genotype distribution and mutations of HBV infection by using the MEGA 7 software.

Results: Of 1425 participants, 15 pregnant women (1.05%, 95% CI: 0.64%-1.73%) were positive for HBsAg, 41 women (2.88%, 95% CI: 2.10%-3.88%) were positive for HBcAb, and 5 women (0.35%, 95% CI: 0.15% –0.82%) had HBV viremia with genotype D, sub-genotype D3 and subtype ayw2. One of the viremic samples was positive for HBcAb but negative for HBsAg, which is indicative of an occult HBV infection. HBsAg seroprevalence was higher among pregnant women aged 20 to 29 years, women in the third trimester of pregnancy, residents of Khormuj city, Afghan immigrants, illiterate women, and pregnant women with the history of tattoo and HBV vaccination. The highest rate of HBcAb seroprevalence was observed in residents of Borazjan city, Turk ethnicity, the age group >39 years, and those women with more parities and a history of abortion. Nevertheless, HBV seroprevalence among pregnant women

was not statistically associated with these variables. In contrast, HBcAb seropositivity was significantly associated with the history of tattoo (*P* = 0.018). According to mutations analyses, seven amino acid substitutions in the HBsAg, one point mutation in the pre-C region, and five points mutations in the BCP region were detected. Besides, the BCP mutations caused amino acid substitutions in the X protein.

Conclusion: These results indicate a relatively low prevalence of HBV infection among pregnant women in the South of Iran, while tattooing is a risk factor for exposure to HBV infection. Moreover, all of the HBV-positive pregnant women were asymptomatic and unaware of their infection. Therefore, routine screening for HBV markers during pregnancy, appropriate treatment of HBV-infected women, and HBV vaccination are recommended to decrease mother-to-child transmission of HBV.

Keywords: Hepatitis B virus; Pregnancy; Risk factor; Prevalence; Genotype; Mutation; Iran

Introduction

Hepatitis B virus (HBV), a member of the family *Hepadnaviridae*, is characterized by asymptomatic, acute, or chronic hepatitis, which might progress to fulminant hepatic failure, cirrhosis, or hepatocellular carcinoma [1, 2]. Currently, about 250 million of the world population are infected with chronic hepatitis B, and 65 million of those infected are women of childbearing age. HBV-related liver diseases are responsible for approximately 800 000 deaths annually [3].

The HBV genome is a circular partially double-stranded DNA with four overlapping open reading frames: S ORF for surface proteins (HBsAg), P ORF for polymerase (reverse transcriptase), preC/*C* ORF for e antigen (HBeAg) and core protein (HBcAg), and X ORF for X

protein [4-6]. Due to the lack of proofreading function of HBV reverse transcriptase, a high rate of mutations naturally occurs during the replication of the viral genome. The most common mutations are pre-core (pre-C) and basal core promoter (BCP) mutations in the HBV core gene [5, 7]. These mutations affect the expression of HBV e antigen (HBeAg) protein and have a significant association with progressive liver diseases such as liver cirrhosis and HCC [2, 8]. In addition, some mutations in the S region have an important role in the occurrence of occult HBV infection with undetectable levels of HBsAg [9].

Horizontal and perinatal transmissions are the main routes of HBV infection. Horizontal transmission might happen through blood transfusion, intravenous drug use, surgery, tattooing, and sexual intercourse [10, 11]. Perinatal or mother-to-child transmission is one of the most common modes of HBV transmission worldwide. Perinatal transmission of HBV most often occurs during birth and delivery [12, 13]. The risk of HBV infection in children born to mothers who are HBsAg-positive and HBeAg-positive is 70% to 90%, and over 90% of these children eventually develop chronic HBV infection, with approximately 25% progressing to cirrhosis and hepatocellular carcinoma [13-15]. This high rate of chronic HBV infection indicates a worse prognosis in infants. However, diagnosis of HBV infection during pregnancy or before delivery and timely interventions can mitigate the risk of HBV transmission to fetus or newborn and, consequently, reduce the disease burden in the community [3, 16].

Iran, with a prevalence rate of 3% in the general population, is considered one of the intermediate endemic countries for HBV infection [17]. The initiation of HBV vaccination of infants since 1993 and teenagers since 2006 in the country has had a significant role in reducing the prevalence of HBV infection in the community [18]. Considering the high rate of chronic HBV carriers among those patients who acquire HBV infection perinatally from infected

mothers, perinatal transmission is probably the dominant route of HBV infection transmission in Iran [19]. Therefore, the prevention of maternal infection and its transmission to the fetuses or newborns is essential, so the diagnosis of infected pregnant women is of particular importance. Nevertheless, no report is available on the prevalence of HBV infection among the pregnant population in the South of Iran, and despite the profound effects of HBV genotypes and mutants on the progression and treatment of HBV infection, little is known about the molecular analysis of HBV infection in Iran. Therefore, this study was performed to investigate the prevalence, risk factors, and genotype distribution of HBV infection and associated mutations in the S, X, pre-C, and BCP regions of the HBV genome among pregnant women resident in the northern shores of the Persian Gulf, South of Iran.

Subjects and methods

Study setting and population

The study was conducted from January 2018 to June 2019 and included 1425 pregnant women from the public health centers in five cities of the northern shores of the Persian Gulf. The public health centers were chosen according to the multi-stage cluster sampling method. In the first stage, Bushehr, Ahram, Borazjan, Jam, and Khormuj cities of this territory were selected randomly. In the second stage, three public health centers from each city were selected randomly, and all the pregnant women attending these centers for routine visits were included consecutively in this study. The leftover serum samples of pregnant women were used for HBV detection. The pregnant women gave written informed consent to participate in the study and use their serum samples for HBV detection. The socio-demographic characteristics, pregnancy information, history of HBV vaccination, the potential risk factors associated with HBV

infection, and levels of liver enzymes were obtained by interviewing each pregnant woman using a questionnaire and from the clinical records of the participants at the public health centers. The Ethics Committee of the Bushehr University of Medical Sciences approved this study with reference number IR.BPUMS.REC.1396.111.

Laboratory methods

HBV serological testing

Serum samples of 1425 pregnant women were tested manually for the presence of hepatitis B surface antigen (HBsAg) and hepatitis B core antibody (HBcAb) using commercially available ELISA kits (HBsAg one – Version ULTRA and HBc Ab ELISA kits, DIA.PRO, Milan, Italy) according to the manufacturer's instructions. The specificity and sensitivity of these kits were 100%. The HBV seropositive pregnant women were tested for HCV and HIV coinfections by ELISA kits (HCV Ab and HIV Ab/Ag ELISA kits, DIA.PRO, Milan, Italy).

PCR amplification and sequencing

The seropositive samples were tested to evaluate the genotype distribution and mutations of HBV ~~infection~~ by two nested PCR assays, targeting S, X, pre-C and BCP regions of the HBV genome [20-24]. Following the extraction of HBV DNA using the High Pure Viral Nucleic Acid kit (Roche, Mannheim, Germany), part of the S region was amplified in the first round PCR using primers 244-HBS-F1 (GAGTCTAGACTCGTGGTGGACTTC) and 691-HBS-R1 (AAATKGCACTAGTAAACTGAGCCA). The second-round PCR was performed using primers 255-HBS-F2 (CGTGGTGGACTTCTCTCAATTTTC) and 671-HBS-R2 (GCCARGAGAAACGGRCTGAGGCCC). The 416 bp length fragments from the S region were

sequenced by Sanger dideoxy sequencing technology to determine HBV genotypes (Macrogen Co., Korea).

Phylogenetic and mutational analysis

The obtained sequences were aligned and compared with the reference sequences representing the standard HBV genotypes available at the nucleotide database of the NCBI by ClustalW program in the Molecular Evolutionary Genetics Analysis (MEGA) software version 7.0 (Biodesign Institute, Tempe, AZ, USA), and the phylogenetic tree was constructed by the neighbor-joining method, as described previously [25].

To determine mutations in BCP and pre-C regions, 789 nucleotides and 740 nucleotides of the X and pre-C region were amplified by nested PCR using outer primers [forward primer (1606-pre-C-F1): GCATGGAGACCACCGTGAAC; reverse primer (2395-pre-C-R1): AGGCGAGGGAGTTCTTCTTC] and inner primers [forward primer (1653-pre-C-F2): CATAAGAGGACTCTTGGACT; reverse primer (2393-pre-C-R2): GCGAGGGAGTTCTTCTTC], respectively. The amplified 740 bp length fragments were sequenced, aligned, and compared with the reference sequences of X and pre-C region of HBV by the ClustalW program in the MEGA 7 software. The S, X, and pre-C sequences isolated from pregnant women were submitted to the GenBank sequence database. The sequences of primers and regions in the HBV genome for detection of HBV genotypes and mutants are summarized in Table 1.

Table 1. Sequences of primers for amplification of S, X, and pre-core regions of HBV genome.

Virus	Primers Name	Sequences of Primers 5′→3′	Gene	Region in Genome	Annealing temperature	Size	References
HBV	244-HBS-F1	GAGTCTAGACTCGTGGTGGACTTC	S	244-267	56 ℃	447 bp	[20, 21]
	691-HBS-R1	AAATKGCACTAGTAAACTGAGCCA		668-691			
	255-HBS-F2	CGTGGTGGACTTCTCTCAATTTTC		255-278	56 ℃	416 bp	
	671-HBS-R2	GCCARGAGAAACGGRCTGAGGCCC		648-671			
HBV	1606-pre-C-F1	GCATGGAGACCACCGTGAAC	X and pre-core region	1606-1625	57 ℃	789 bp	[22-24]
	2395-pre-C-R1	AGGCGAGGGAGTTCTTCTTC		2376-2395			
	1653-pre-C-F2	CATAAGAGGACTCTTGGACT		1653-1672	55 ℃	740 bp	
	2393-pre-C-R2	GCGAGGGAGTTCTTCTTC		2376-2393			

Statistical analysis

The χ^2 test or Fisher's exact test was used to analyze and compare categorical variables between HBV-positive and HBV-negative pregnant women. Quantitative variables were compared by the Student's *t*-test. Univariate and multivariate logistic regression analyses were performed to evaluate the risk factors associated with HBV infection among pregnant women. The data were analyzed by SPSS 17 package program (SPSS Inc., Chicago, IL, USA), and P values < 0.05 were defined statistically significant.

Results

Of 1425 pregnant women, 616 women were from Bushehr city, 207 were from Ahram city, 40 were from Khormuj city, 440 were from Borazjan city and 122 were from Jam city. The mean age ± SD of pregnant women was 28.1±5.99 years with a range of 14-46 years. The majority of pregnant women were in the third trimester of pregnancy (68.4%), the age group 20–29 years (55.2%), educated (97.1%), and Fars (90.0%). Of 1425 participants, 15 pregnant women (1.05%, 95% CI: 0.64%-1.73%) were positive for HBsAg, and 41 women (2.88%, 95% CI: 2.10%-

3.88%) had HBcAb. Moreover, 4 of 41 HBcAb seropositive samples (9.75%) were also positive for HBsAg.

HBsAg seropositive pregnant women had a lower mean age (27.53±5.96) compared to HBsAg seronegative pregnant women (28.11±5.99), ~~while~~ this difference was not statistically significant (*P* = 0.71). Moreover, there was no difference between the mean age of HBcAb seropositive pregnant women (28.88±5.96) and HBcAb seronegative pregnant women (28.08±5.99) (*P* = 0.402). The majority of HBsAg seropositive women were in the age group 20-29 years (1.4%), the third trimester of pregnancy (1.3%), residents in Khormuj city (2.5%), Afghan (1.6%), illiterate (2.4%) and had a history of tattoo (1.6%) and HBV vaccination (1.6%) (S1 Table). The highest rate of HBcAb seroprevalence was observed in residents of Borazjan city (3.9%), Turk and Afghan immigrants (8.3% and 6.5%, respectively), the age group >39 years (4.3%), illiterate pregnant women (7.1%), pregnant women in the second trimester of pregnancy (3.6%), and those women with more parities (5.0%) and a history of abortion (3.0%) (S2 Table). However, none of these variables were statistically associated with HBsAg and HBcAb seroprevalence. In contrast, HBcAb seropositivity was significantly associated with history of tattoo, so that tattooing was a risk factor for exposure to HBV infection (OR: 1.47; 95% CI: 1.06-2.04; *P* = 0.018) (S2 Table). In addition, HBcAb seropositivity was more prevalent in those samples collected in October (11.5%) compared to the other months. However, according to the multivariate logistic regression analysis, the difference in the monthly rate of HBcAb seroprevalence was statistically insignificant. All of the HBV seropositive samples had normal levels of liver enzymes and were negative for HCV and HIV. The prevalence of HBsAg and HBcAb among pregnant women grouped according to socio-demographic characteristics and qualitative variables are presented in S1 and S2 Tables, respectively.

According to the molecular testing, 5 women (0.35%, 95% CI: 0.15% – 0.82%) had HBV viremia. Of viremic samples, 3 samples were positive for HBsAg, 1 sample was positive for both HBsAg and HBcAb, and 1 sample was positive for HBcAb but negative for HBsAg, which is indicative of an occult HBV infection. Notably, 3 samples were positive in the first round of nested PCR, and 2 samples were found to be positive in the second round of nested PCR (Figs 1 and 2). Overall, 26.7% of HBsAg seropositive pregnant women and 2.7% of HBcAb seropositive women had HBV viremia with genotype D, sub-genotype D3, and subtype ayw2 (GenBank accession Nos. OK490614-OK490616) (Fig 3). Seven amino acid substitutions were detected in HBsAg of strains isolated from pregnant women, including the conversion of Ile → Leu at amino acid 110 (I110L), Thr → Pro at amino acid 127 (T127P), Asp → Glu at amino acid 144 (D144E), Ser → Ala at amino acid 154 (S154A), Gly → Ala at amino acid 159 (G159A), Ala → Gly at amino acid 166 (A166G) and Ala → Val at amino acid 168 (A168V) of HBsAg (S1 Fig and Table 2). Moreover, a G to A substitution at nucleotide position 1896 (G1896A) was detected in the pre-C region (S2 Fig). T1753A, A1761C, G1764T/A, C1766G/T, and C1773T mutations were detected in the BCP region. Of these, T1753A, A1761C and G1764A mutations cause the conversion of Ile → Asn at amino acid 127 (I127N), Lys → Gln at amino acid 130 (K130Q), and Val → Leu at amino acid 131 (V131L) of the X protein, respectively (S3 Fig).

Table 2. Frequency of point replacement mutations in HBsAg of strains isolated from pregnant women in the South of Iran.

Position S mutation	Frequency of point mutation No. (%)	Patient No.
I110L	1 (33%)	HB55
T127P	2 (66%)	HB51, HB55
D144E	1 (33%)	HB35
S154A	1 (33%)	HB55
G159A	1 (33%)	HB55
A166G	1 (33%)	HB35
A168V	2 (66%)	HB51, HB55

Fig 1. Electrophoresis of PCR products of S region of HBV genome extracted from serum samples of pregnant women on 2% agarose gel. L, 100-bp DNA ladder; N, negative control; P, positive control; 1, 2, 3, 4, 6, 7 and 9, amplified products (≈417 bp).

Fig 2. Electrophoresis of PCR products of X and pre-core regions of HBV genome extracted from serum samples of pregnant women on 2% agarose gel. L, 100-bp DNA ladder; Neg, negative control; Pos, positive control; Sam, amplified product (≈740 bp).

Fig 3. Neighbor-joining phylogenetic tree based on ~380 bp nucleotide sequence (~270-670 bp of the complete reference genome) of S region of HBV isolates (green mark) from the serum samples of pregnant women in South of Iran (GenBank accession Nos. OK490614-OK490616). Bootstrap resampling strategy and reconstruction were carried out 1,000 times to confirm the reliability of the phylogenetic tree.

Discussion

HBV causes chronic infections in 2% to 3% of adults and 95% of infants and is an important factor in the development of advanced chronic liver disease and hepatocellular carcinoma in these patients [3]. Notably, perinatal or mother-to-infant transmission is responsible for the occurrence of over 50% of chronic HBV carriers [13]. Considering the high rates of perinatal transmission and chronic HBV infection in infants, diagnosis of HBV infection among pregnant women is of great importance. Despite this importance, there is no report on the prevalence of HBV infection among pregnant women in the northern shores of the Persian Gulf, South of Iran. Therefore, we evaluated the prevalence and molecular epidemiology of HBV infection among pregnant women in this region and found HBV prevalence of 1.05% for HBsAg, 2.88% for HBcAb and 0.35% for HBV viremia with genotype D, sub-genotype D3 and subtype ayw2.

The HBsAg seroprevalence of 1.05% observed in pregnant women is considerably higher than the HBsAg prevalence of 0.15% reported in the blood donors of this region [18]. Besides, the HBV prevalence noted in this study is slightly lower than the overall prevalence of HBV among Iranian pregnant women (1.18%) [15]. In this study, all of the HBV seropositive pregnant

women were asymptomatic and unaware of their infection. These infected but asymptomatic pregnant women may remain undiagnosed over time. The risk of HBV infection in children born to infected mothers is 70% to 90%, and over 90% of these children eventually develop chronic HBV infection, with approximately 25% progressing to cirrhosis and hepatocellular carcinoma [13-15]. This high rate of chronic HBV infection indicates a worse prognosis in infants. Routine screening for HBV infection during pregnancy is therefore recommended for the identification of HBV-infected pregnant women and neonates born at risk. Maternal treatment with oral antivirals such as tenofovir, telbivudine, and lamivudine and immunoglobulin administration at birth can prevent mother-to-child transmission of HBV. Consequently, this reduces the burden of chronic HBV infection in the community [3, 16]. According to the results of this study, 0.35% of pregnant women had HBV viremia. Prenatal transmission, which is leading to postpartum immunoprophylaxis incompetence, is possible in women with HBV viremia [14]. Since, a significant percentage of pregnant women were not vaccinated, HBV vaccination is recommended to reduce the incidence of HBV infection during pregnancy.

The prevalence of 1.05% for HBsAg observed in the present study is higher than those observed among pregnant women in other parts of Iran, 0.18% in Babol (North of Iran) [26], 0.3% in Mashhad (North-East of Iran) [27], 0.59% in Dehloran (West of Iran) [28], 0.7% in Lorestan (West of Iran) [29], 0.77% in Kurdistan (West of Iran) [30] and 0.87% in East Azerbaijan (North-West of Iran) [30] but lower than 1.3% in Rafsanjan (South-East of Iran) [31], 1.93% in Golestan (North of Iran) [30], 2.26% in Jiroft (South-East of Iran) [30] and 2.5% in Malekan (North-West of Iran) [32]. The HBsAg prevalence noted in this study is also higher than those observed among pregnant women of some other countries, 0.9% in Brazil [33] but lower than those reported among pregnant women in Ghana (3.3%) [35], Central Nigeria (5.5%) [36],

Shenyang, China (5.5%) [37], Ethiopia (7.9%) [38], Gambia (9.2%) [39], North Cameroon (10.2%) [40] and Yemen (10.8%) [41]. Moreover, the prevalence of 2.88% for HBcAb reported in this study is higher than that observed among pregnant women in Malekan (1.25%) [32] but lower than that in Lorestan (3.4%) [29]. This prevalence is lower than those of other countries such as Brazil (7.4%) [33] and Shenyang, China (29.65%) [37]. These variations in the seroprevalence of HBV are probably because of differences in rates of high-risk behaviors, level of awareness, immunization status, the routes of transmission, epidemiology of HBV, and burden of the infection in different regions. However, differences in sociodemographic characteristics of the participants and the sensitivity and specificity of the diagnosis assays in different studies can also explain these variations.

In this study, HBV seroprevalence among the pregnant women was not statistically associated with age, stage of gestation, number of pregnancies, place of residency, level of education, ethnicity, and history of blood transfusion, abortion, dentistry, surgery and HBV vaccination, although the majority of HBsAg seropositive pregnant women were in the age group 20-29 years, the third trimester of pregnancy, residents in Khormuj city, illiterate, Afghan and had a history of tattoo and HBV vaccination. Furthermore, the highest rate of HBcAb seroprevalence was observed in residents of Borazjan city, Turk and Afghan immigrants, the age group >39 years, illiterate pregnant women, pregnant women in the second trimester of pregnancy, and those women with more parities and a history of abortion. The higher seroprevalence of HBV among pregnant women resident in Khormuj and Borazjan cities might be due to the higher prevalence of HBV infection in the general population of these cities. In contrast, HBcAb seropositivity was significantly associated with the time of sampling and history of tattoo, so that tattooing was a risk factor for exposure to HBV infection. In addition, HBcAb seropositivity was

more prevalent in those samples collected in October compared to the other months. The reason for this monthly pattern of HBcAb seroprevalence is not clear.

A recent meta-analysis study revealed that HBsAg seroprevalence among pregnant women in Iran is associated with the history of blood transfusion, abortion, illiteracy, and intravenous drug use by spouses [15]. In a study from the South-East of Iran, HBsAg seroprevalence among pregnant women was associated with illicit drug use and tattooing [31]. In another study from the West of Iran, more parities and abortion were risk factors for exposure to HBV [28]. In a recent study from Yemen, a significant association was found between female circumcision and HBsAg seropositivity [41]. Another study from North Cameroon reported a significant association between HBV infection and a history of blood transfusion [40]. Previous studies from Ethiopia demonstrated that HBsAg seroprevalence among pregnant women is associated with multiple sexual partners, history of abortion, dentistry, and surgery [38, 42]. This discrepancy between reports might be due to differences in the predominant routes of transmission and risk behavior patterns in different geographical regions.

In the present study, the highest rate of HBsAg seroprevalence was observed in pregnant women aged 20 to 29 years (1.4%). Considering the compulsory vaccination of infants since 1993 and teenagers since 2006 [18], this is probably due to the partial ineffectiveness or incompleteness of the national HBV vaccination program in this age group. However, further studies are required to determine the efficacy of hepatitis B vaccination among pregnant women in this region. Moreover, there was no difference between HBV-vaccinated and HBV-unvaccinated pregnant women regarding the prevalence of HBsAg. Therefore, although there are effective tools and strategies for the prevention and treatment of HBV infection, management of HBV infection during pregnancy faces challenges that need further investigation. On the other hand, all

pregnant women under 20 years old were negative for HBsAg, which is probably due to the initiation of neonatal vaccination in the country in 1993, awareness of HBV infection among youths, and less exposure to HBV or less engagement in risky behaviors in young ages. In contrast, a recent study in Central Nigeria demonstrated a higher prevalence of HBsAg among pregnant women under 20 years old [36].

In this study, occult HBV infection was detected in one sample with undetectable levels of HBsAg and low HBV DNA levels. Nevertheless, P120T and G145R mutations associated with failure of HBsAg detection were not found in this sample. P120T and G145R mutations in the S gene are associated with low responses to HBV vaccination and immunoglobulin therapy as well as failures of diagnostic tests [43, 44]. In this study, one point mutation in the pre-C region and five points mutations in the BCP region were detected. Of these, the pre-C mutation (G1896A) eliminates HBeAg expression at the translational level, whereas BCP mutations (G1764A) decrease HBeAg expression at the transcriptional level [5]. HBeAg is an immune target, and lack of its expression may result in loss of immune responses and subsequently liver disease progression [7, 45]. Notably, the sample with G1896A mutation was HBeAg negative. The G1896A mutation causes the conversion of TGG→TAG (Trp → Stop codon) at codon 28 of the pre-C gene and the suppression of HBeAg expression. Whereas, the BCP mutations enhance the replication rate of the HBV genome [5]. The BCP and pre-C mutations are associated with the progression of chronic HBV infection to advanced liver disease and are frequently found in patients with chronic hepatitis, fibrosis, liver cirrhosis, and HCC [2, 7]. Therefore, early detection of these mutations is pivotal in predicting the clinical progression in patients with chronic HBV infection and deciding the antiviral medications. No nucleotide insertions, deletions, or frameshifting were observed in our samples.

The dominance of genotype D is the most important characteristic of HBV infection among our pregnant women, which reflects the genotypic pattern of HBV infection in our region. Genotype D is also the predominant genotype in Iran [46]. To date, 10 HBV genotypes (A to J) with 9 serological subtypes and over 35 sub-genotypes related to genotypes A–D, F, H, and I have been identified to be not only predictive of response to antiviral treatment and disease progression but also associated with modes of transmission and geographical regions [6, 10]. Of them, genotype D is the main genotype in Africa, Europe, India, Indonesia and the Mediterranean countries, and is characterized by chronicity, worse clinical outcomes (cirrhosis and HCC), low response to IFN-based therapy, and a high frequency of BCP and pre-C mutations [10, 11]. The pre-C G1896A mutation is frequently found in HBV genotypes B, D, and E [45]. Moreover, the BCP A1762T and G1764A mutations are reported in association with HBV genotypes C and D [47]. On the other hand, sub-genotype D3 is prevalent among injecting drug users [48]. Like HCV infection [49], injecting drug use is most probably the main route of HBV transmission in this region. However, we were unable to confirm this claim, since the majority of women were not willing to answer the questions regarding the intravenous drug use by themselves or more importantly by their spouses.

This is the first report on the prevalence of HBV infection among pregnant women resident in the northern shores of the Persian Gulf, and the first to investigate the seroprevalence of HBsAg and HBcAb among pregnant Afghan migrant women in Iran. Moreover, the results of this study can be generalized to the pregnant population of this territory due to the consecutive recruitment of participants and the high number of assessed pregnant women. However, due to the cross-sectional design of the study, we were not able to determine the eventual effects of hepatitis B on pregnancy outcomes and the infants born to infected mothers. Therefore, this report should be

completed by prospective studies. Besides, due to the lack of awareness of the level of immunity against hepatitis B among the study population, this study was not able to determine the efficacy of hepatitis B vaccination among pregnant women in this region. Nevertheless, antibody to hepatitis B surface antigen (anti-HBsAg) was not assessed among the study population. Therefore, there is a need to study the effectiveness of the immunization policy in the pregnant population of this region.

In this study, the nested-PCR assay was used to detect HBV infection among pregnant women. This two-step PCR method has a very high sensitivity for the diagnosis of cases with low virus levels. However, in comparison with quantitative PCR (qPCR), the nested-PCR assay is not able to determine the viral loads. Moreover, the main limitation of nested-PCR is the probability of contamination that can cause false-positive results. In this study, strict quality control was applied, and positive serum samples were re-tested to ensure the absence of contamination and the accuracy of the results.

Conclusion

The results of this study indicate a relatively low prevalence of HBV infection among pregnant women in the South of Iran, while tattooing is a risk factor for exposure to HBV infection. Moreover, all of the HBV-positive pregnant women were asymptomatic and unaware of their infection. Therefore, routine screening for HBV markers during pregnancy, appropriate treatment of HBV-infected women, and HBV vaccination are recommended to decrease mother-to-child transmission of HBV and, consequently, reduce the disease burden in the community. Moreover, we observed occult HBV infection in one sample with an undetectable level of HBsAg. Therefore, screening of pregnant women based on HBsAg detection is not reliable for

the identification of occult HBV infection. The use of more sensitive molecular techniques such as nested-PCR assay is recommended to detect HBV DNA and increase the chance of diagnosing occult hepatitis B with low virus titers. Besides, several point replacement mutations were detected in the S, X, pre-C, and BCP regions of HBV isolated from pregnant women. Mutation analysis of HBV can be useful in predicting the disease progression in HBV-infected patients.

Acknowledgment

The authors would like to thank the Deputy Research and Affairs of the Bushehr University of Medical Sciences, Bushehr, Iran.

References

1. Ghabeshi S, Baktashi R, Hosseini SM, Sharifi Z. Molecular evaluation of HBV core gene mutations in asymptomatic HBV infected blood donors in Iran. 2014.
2. Chachá SGF, Gomes-Gouvêa MS, Malta FdM, Ferreira SdC, Villanova MG, Souza FF, et al. Basal core promoter and precore mutations among hepatitis B virus circulating in Brazil and its association with severe forms of hepatic diseases. Memórias do Instituto Oswaldo Cruz. 2017;112(9):626-31.
3. Joshi SS, Coffin CS. Hepatitis B and Pregnancy: Virologic and Immunologic Characteristics. Hepatol Commun. 2020;4(2):157-71. Epub 2020/02/07. doi: 10.1002/hep4.1460. PubMed PMID: 32025602; PubMed Central PMCID: PMCPMC6996345.
4. Rajput MK. Mutations and methods of analysis of mutations in Hepatitis B virus. AIMS microbiology. 2020;6(4):401.
5. Tacke F, Gehrke C, Luedde T, Heim A, Manns MP, Trautwein C. Basal core promoter and precore mutations in the hepatitis B virus genome enhance replication efficacy of Lamivudine-resistant mutants. J Virol. 2004;78(16):8524-35. Epub 2004/07/29. doi: 10.1128/JVI.78.16.8524-8535.2004. PubMed PMID: 15280461; PubMed Central PMCID: PMCPMC479060.
6. Kramvis A. Genotypes and genetic variability of hepatitis B virus. Intervirology. 2014;57(3-4):141-50. Epub 2014/07/19. doi: 10.1159/000360947. PubMed PMID: 25034481.
7. Yan L, Zhang H, Ma H, Liu D, Li W, Kang Y, et al. Deep sequencing of hepatitis B virus basal core promoter and precore mutants in HBeAg-positive chronic hepatitis B patients. Sci Rep. 2015;5:17950. Epub 2015/12/10. doi: 10.1038/srep17950. PubMed PMID: 26647737; PubMed Central PMCID: PMCPMC4673698.

8. Ledesma MM, Galdame O, Bouzas B, Tadey L, Livellara B, Giuliano S, et al. Characterization of the basal core promoter and precore regions in anti-HBe-positive inactive carriers of hepatitis B virus. Int J Infect Dis. 2011;15(5):e314-20. Epub 2011/03/04. doi: 10.1016/j.ijid.2010.12.009. PubMed PMID: 21367634.
9. El Chaar M, Candotti D, Crowther RA, Allain JP. Impact of hepatitis B virus surface protein mutations on the diagnosis of occult hepatitis B virus infection. Hepatology. 2010;52(5):1600-10. Epub 2010/09/04. doi: 10.1002/hep.23886. PubMed PMID: 20815025.
10. Lin CL, Kao JH. Hepatitis B virus genotypes and variants. Cold Spring Harb Perspect Med. 2015;5(5):a021436. Epub 2015/05/03. doi: 10.1101/cshperspect.a021436. PubMed PMID: 25934462; PubMed Central PMCID: PMCPMC4448583.
11. Sunbul M. Hepatitis B virus genotypes: global distribution and clinical importance. World J Gastroenterol. 2014;20(18):5427-34. Epub 2014/05/17. doi: 10.3748/wjg.v20.i18.5427. PubMed PMID: 24833873; PubMed Central PMCID: PMCPMC4017058.
12. Chilaka VN, Konje JC. Viral Hepatitis in pregnancy. Eur J Obstet Gynecol Reprod Biol. 2021;256:287-96. Epub 2020/12/02. doi: 10.1016/j.ejogrb.2020.11.052. PubMed PMID: 33259998.
13. Piratvisuth T. Optimal management of HBV infection during pregnancy. Liver International. 2013;33:188-94.
14. Navabakhsh B, Mehrabi N, Estakhri A, Mohamadnejad M, Poustchi H. Hepatitis B virus infection during pregnancy: transmission and prevention. Middle East journal of digestive diseases. 2011;3(2):92.
15. Badfar G, Shohani M, Nasirkandy MP, Mansouri A, Abangah G, Rahmati S, et al. Epidemiology of hepatitis B in pregnant Iranian women: a systematic review and meta-analysis. Arch Virol. 2018;163(2):319-30. Epub 2017/10/25. doi: 10.1007/s00705-017-3551-6. PubMed PMID: 29063378.
16. Brown RS, Jr., McMahon BJ, Lok AS, Wong JB, Ahmed AT, Mouchli MA, et al. Antiviral therapy in chronic hepatitis B viral infection during pregnancy: A systematic review and meta-analysis. Hepatology. 2016;63(1):319-33. Epub 2015/11/14. doi: 10.1002/hep.28302. PubMed PMID: 26565396.
17. Mohammadi Z, Keshtkar A, Eghtesad S, Jeddian A, Pourfatholah AA, Maghsudlu M, et al. Epidemiological profile of hepatitis B virus infection in Iran in the past 25 years; a systematic review and meta-analysis of general population studies. Middle East journal of digestive diseases. 2016;8(1):5.
18. Farshadpour F, Taherkhani R, Tajbakhsh S, Gholizadeh Tangestani M, Hajiani G, Sharifi N, et al. Prevalence and trends of transfusion-transmissible viral infections among blood donors in south of Iran: an eleven-year retrospective study. PloS one. 2016;11(6):e0157615.
19. Moghaddasifar I, B Lankarani K, Moosazadeh M, Afshari M, Malary M. Prevalence of hepatitis B virus infection among pregnant women in Iran: a systematic review and Meta-analysis. Iranian Journal of Cancer Prevention. 2016;9(6).
20. Sitnik R, Pinho JR, Bertolini DA, Bernardini AP, Da Silva LC, Carrilho FJ. Hepatitis B virus genotypes and precore and core mutants in Brazilian patients. J Clin Microbiol. 2004;42(6):2455-60. Epub 2004/06/09. doi: 10.1128/JCM.42.6.2455-2460.2004. PubMed PMID: 15184419; PubMed Central PMCID: PMCPMC427827.
21. Chen J, Yin J, Tan X, Zhang H, Zhang H, Chen B, et al. Improved multiplex-PCR to identify hepatitis B virus genotypes A-F and subgenotypes B1, B2, C1 and C2. J Clin Virol.

2007;38(3):238-43. Epub 2007/01/17. doi: 10.1016/j.jcv.2006.12.007. PubMed PMID: 17224304.

22. Takahashi K, Aoyama K, Ohno N, Iwata K, Akahane Y, Baba K, et al. The precore/core promoter mutant (T1762A1764) of hepatitis B virus: clinical significance and an easy method for detection. J Gen Virol. 1995;76 (Pt 12):3159-64. Epub 1995/12/01. doi: 10.1099/0022-1317-76-12-3159. PubMed PMID: 8847524.

23. Boot HJ, Cremer J, Koedijk FD, van Ballegooijen WM, Op de Coul EL. Improved tracing of hepatitis B virus transmission chains by phylogenetic analysis based on C region sequences. J Med Virol. 2008;80(2):233-41. Epub 2007/12/22. doi: 10.1002/jmv.21080. PubMed PMID: 18098131.

24. Italiano CM, Speers DJ, Chidlow GR, Dowse GK, Robertson AG, Flexman JP. Hepatitis B outbreak following a mass-casualty incident, Australia. J Infect Dis. 2011;204(3):400-7. Epub 2011/07/12. doi: 10.1093/infdis/jir269. PubMed PMID: 21742838.

25. Taherkhani R, Farshadpour F, Makvandi M, Hamidifard M, Esmailizadeh M, Ahmadi B, et al. Determination of cytomegalovirus prevalence and glycoprotein B genotypes among ulcerative colitis patients in Ahvaz, Iran. Jundishapur Journal of Microbiology. 2015;8(2).

26. Bayani M, Biazar T, HASANJANI RM, Bayani F, Siadati S. The effect of hepatitis B vaccination at birth on reducing the prevalence of hepatitis B surface antigen among rural pregnant women in Babol, Iran. 2016.

27. Pourafzali SJ, Khaledi A, Miri R, Shamsian SAA, Rezaee SA, Piroozmand A, et al. Prevalence of Hepatitis B Surface Antigen and Associated Risk Factors with Hepatitis B Infection among Pregnant Women in Northeast of Iran. International Journal of Medical Laboratory. 2018;5(3):222-8.

28. Kheiri L, Makvandi S. The prevalence of Hepatitis B surface antigen (HBsAg) and its influencing factors in pregnant women referring to Healthcare Centers of Dehloran, Iran in 2011-2012. Journal of Midwifery and Reproductive Health. 2015;3(3):424-9.

29. Mohebbi SR, Sanati A, Cheraghipour K, Nejad MR, Shalmani HM, Zali MR. Hepatitis C and hepatitis B virus infection: epidemiology and risk factors in a large cohort of pregnant women in Lorestan, West of Iran. Hepatitis monthly. 2011;11(9):736.

30. Shoghli A, Nabavi SM, Alavian SM, Kolifarhood G, Goya MM, Namazi R, et al. Hepatitis B surface antigen prevalence in pregnant women: A cross-sectional survey in Iran. International journal of preventive medicine. 2014;5(Suppl 3):S213.

31. Aminzadeh Z, Shabani Shahrbabaki Z, Ghachkar L, Sayyadi Anari AR. Hepatitis B Surface Antigen Prevalence among Pregnant Women in Rafsanjan City, Iran, 2003. Iranian Journal of Virology. 2008;2(3):19-20.

32. Sahaf F, Tanomand A, Montazam H, Sany A. Seroprevalence of hepatitis C, hepatitis B and HIV and co-infection among pregnant women: A retrospective study in 2006 at Malekan city, Iran. Res J Med Sci. 2007;1(2):138-41.

33. Souza MT, Pinho TLRd, Santos MDC, Santos Ad, Monteiro VL, Fonsêca LMB, et al. Prevalence of hepatitis B among pregnant women assisted at the public maternity hospitals of São Luís, Maranhão, Brazil. Brazilian Journal of Infectious Diseases. 2012;16(6):517-20.

34. Rajendiran S, Gopalan U, Jayakumar K. Seroprevalence of hepatitis B infection among pregnant women in South India. International Journal of Reproduction, Contraception, Obstetrics and Gynecology. 2017;6(1):249-52.

35. Kwadzokpui PK, Akorsu EE, Abaka-Yawson A, Quarshie SS, Amankwah SA, Tawiah PA. Prevalence and knowledge of hepatitis B virus infection among pregnant women in the Ningo-Prampram district, Ghana. International journal of hepatology. 2020;2020.
36. Oti V, Mohammed I, Ibrahim Y, Ibrahim C, Orok I. Epidemiologic Survey of HBV, HCV and HIV Infections in a Pregnant Women Population in Central Nigeria: A Cross-Sectional Study. J Infect Dis Epidemiol. 2021;7:194.
37. Ding Y, Sheng Q, Ma L, Dou X. Chronic HBV infection among pregnant women and their infants in Shenyang, China. Virology journal. 2013;10(1):1-5.
38. Tanga AT, Teshome MA, Hiko D, Fikru C, Jilo GK. Sero-prevalence of hepatitis B virus and associated factors among pregnant women in Gambella hospital, South Western Ethiopia: facility based cross-sectional study. BMC infectious diseases. 2019;19(1):1-7.
39. Bittaye M, Idoko P, Ekele BA, Obed SA, Nyan O. Hepatitis B virus sero-prevalence amongst pregnant women in the Gambia. BMC Infect Dis. 2019;19(1):259. Epub 2019/03/17. doi: 10.1186/s12879-019-3883-9. PubMed PMID: 30876397; PubMed Central PMCID: PMCPMC6419830.
40. Noubiap JJN, Nansseu JRN, Ndoula ST, Bigna JJR, Jingi AM, Fokom-Domgue J. Prevalence, infectivity and correlates of hepatitis B virus infection among pregnant women in a rural district of the Far North Region of Cameroon. BMC public health. 2015;15(1):1-7.
41. Murad EA, Babiker SM, Gasim GI, Rayis DA, Adam I. Epidemiology of hepatitis B and hepatitis C virus infections in pregnant women in Sana'a, Yemen. BMC pregnancy and childbirth. 2013;13(1):1-5.
42. Bancha B, Kinfe AA, Chanko KP, Workie SB, Tadese T. Prevalence of hepatitis B viruses and associated factors among pregnant women attending antenatal clinics in public hospitals of Wolaita Zone, South Ethiopia. PloS one. 2020;15(5):e0232653.
43. Roznovsky L, Harrison TJ, Fang ZL, Ling R, Lochman I, Orsagova I, et al. Unusual hepatitis B surface antigen variation in a child immunised against hepatitis B. J Med Virol. 2000;61(1):11-4. Epub 2000/04/04. doi: 10.1002/(sici)1096-9071(200005)61:1<11::aid-jmv2>3.0.co;2-9. PubMed PMID: 10745226.
44. Avellon A, Echevarria JM. Frequency of hepatitis B virus 'a'determinant variants in unselected Spanish chronic carriers. Journal of medical virology. 2006;78(1):24-36.
45. Juniastuti, Utsumi T, Aksono EB, Yano Y, Soetjipto, Hayashi Y, et al. Predominance of precore mutations and clinical significance of basal core promoter mutations in chronic hepatitis B virus infection in Indonesia. Biomed Rep. 2013;1(4):522-8. Epub 2014/03/22. doi: 10.3892/br.2013.106. PubMed PMID: 24648979; PubMed Central PMCID: PMCPMC3917060.
46. Haghshenas MR, Arabi M, Mousavi T. Hepatitis B genotypes in Iran. Mater Sociomed. 2014;26(2):129-33. Epub 2014/06/20. doi: 10.5455/msm.2014.26.129-133. PubMed PMID: 24944540; PubMed Central PMCID: PMCPMC4035145.
47. Lin CL, Kao JH. Natural history of acute and chronic hepatitis B: The role of HBV genotypes and mutants. Best Pract Res Clin Gastroenterol. 2017;31(3):249-55. Epub 2017/08/05. doi: 10.1016/j.bpg.2017.04.010. PubMed PMID: 28774406.
48. Ozaras R, Inanc Balkan I, Yemisen M, Tabak F. Epidemiology of HBV subgenotypes D. Clin Res Hepatol Gastroenterol. 2015;39(1):28-37. Epub 2014/07/20. doi: 10.1016/j.clinre.2014.06.005. PubMed PMID: 25037178.
49. Taherkhani R, Farshadpour F. Lurking epidemic of hepatitis C virus infection in Iran: A call to action. World J Hepatol. 2017;9(24):1040-2. Epub 2017/09/22. doi:

10.4254/wjh.v9.i24.1040. PubMed PMID: 28932350; PubMed Central PMCID: PMCPMC5583536.

Supporting information

S1 Fig. Alignment of the amino acid sequences of HBsAg (44-170 aa) of strains isolated from pregnant women (GenBank accession Nos. OK490614-OK490616) and the reference sequences available at the GenBank.

S2 Fig. Alignment of the amino acid sequences of pre-core region isolated from pregnant women (GenBank accession Nos. OK490617 and OK490618) and the reference sequences available at the GenBank.

S3 Fig. Alignment of the amino acid sequences (109-154 aa) of X protein (1698-1838 bp nucleotide sequence) isolated from pregnant women (GenBank accession Nos. OK490617 and OK490618) and the reference sequences available at the GenBank.

S1 Table. Prevalence of HBsAg according to socio-demographic and qualitative variables among pregnant women in the South of Iran.

S2 Table. Prevalence of HBcAb according to socio-demographic and qualitative variables among pregnant women in the South of Iran.

Figure 1

Click here to access/download;Figure;Fig 1.tif

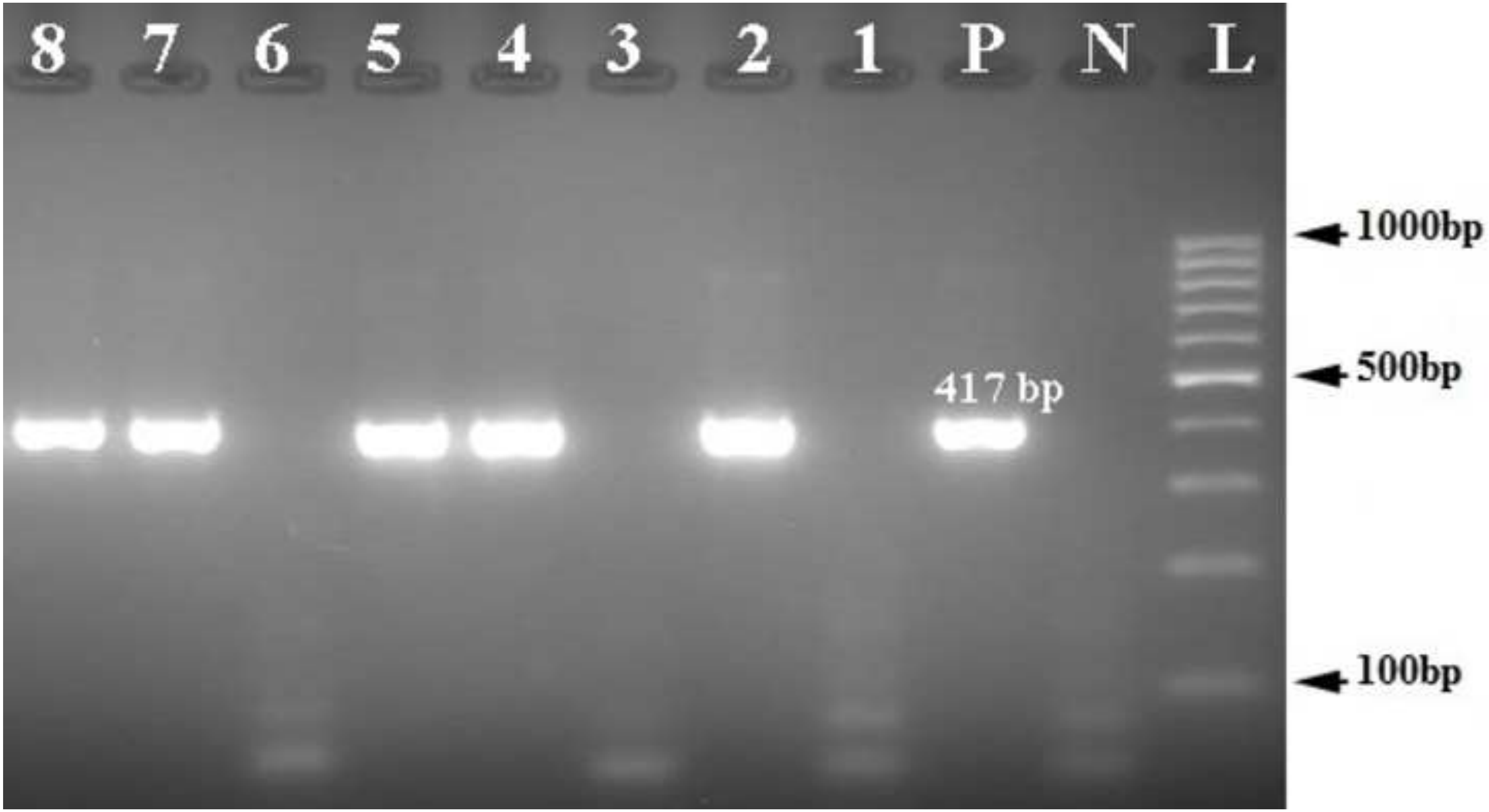

Figure 2

Click here to access/download;Figure;Fig 2.tif

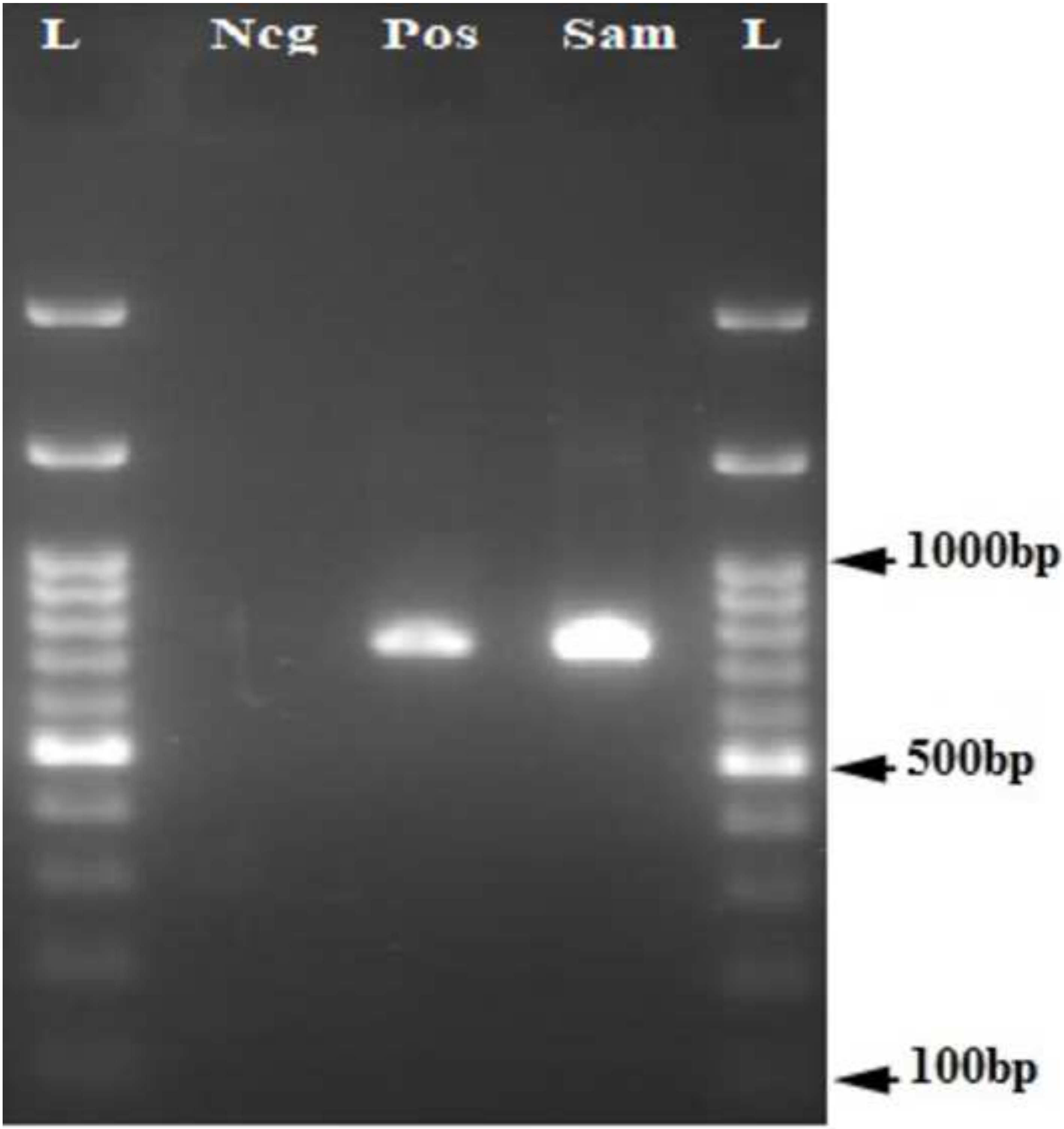

Figure 3

Click here to access/download;Figure;Fig 3.tif

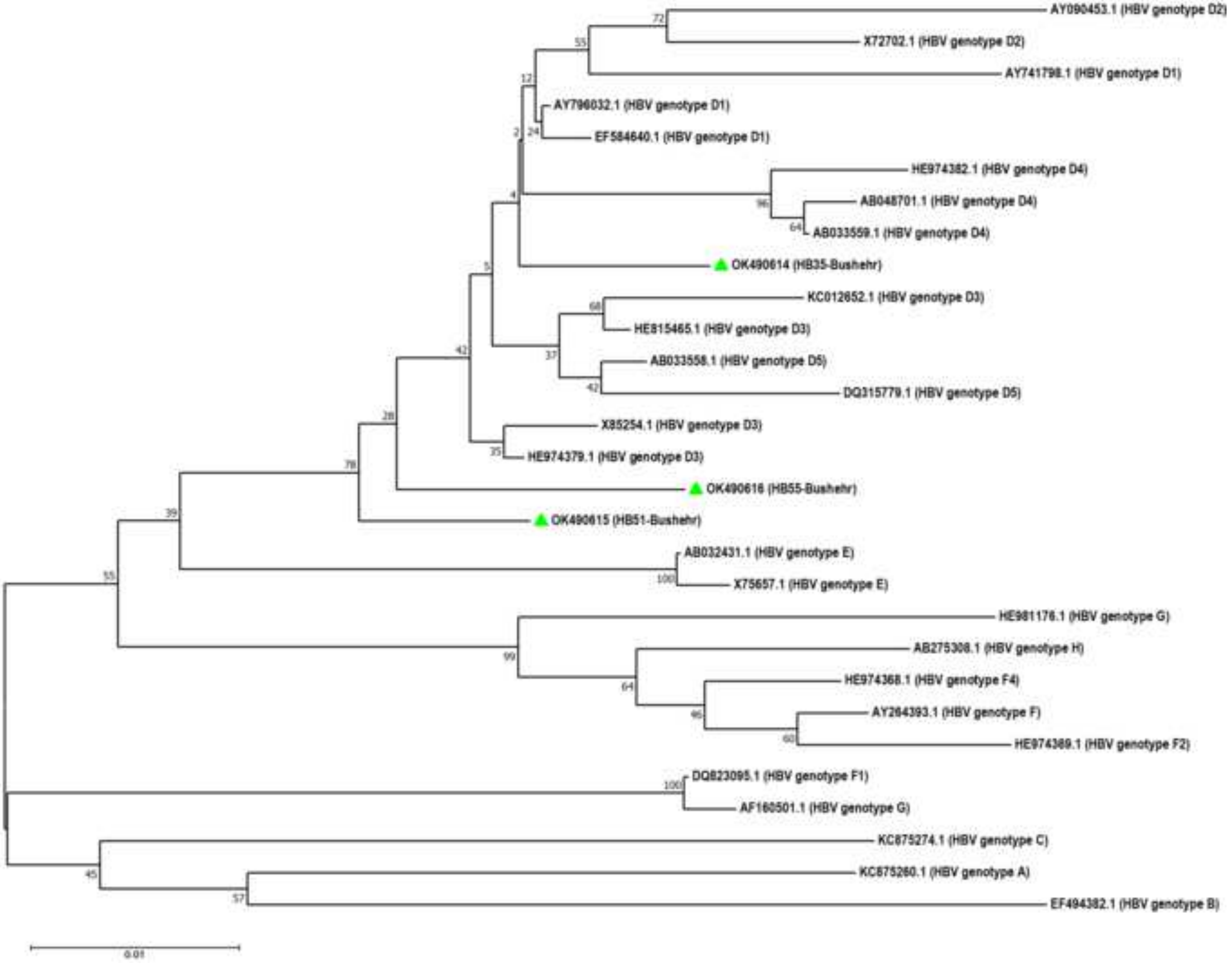

Click here to access/download
Supporting Information
S1_Fig.tif

Click here to access/download
Supporting Information
S2_Fig.tif

Click here to access/download
Supporting Information
S3_Fig.tif

Click here to access/download
Supporting Information
S1_Table.doc

Click here to access/download
Supporting Information
S2_Table.doc

Click here to access/download
Supporting Information
Questionnaire.doc

Click here to access/download
Supporting Information
Fig 1 original.JPG

Click here to access/download
Supporting Information
Fig 2 original.JPG

Prevalence, genotype distribution and mutations of hepatitis B virus infection and associated risk factors among pregnant women ~~resident~~ residing in the northern shores of Persian Gulf, Iran

Formatted: Highlight

Reza Taherkhani and Fatemeh Farshadpour*

Department of Virology, School of Medicine, Bushehr University of Medical Sciences, Bushehr, Iran

~~**Running title:** Hepatitis B virus infection among pregnant women in Iran~~

~~**ORCID Number:**~~

~~Fatemeh Farshadpour (0000-0002-8317-9573); Reza Taherkhani (0000-0001-6499-0531)~~

***Corresponding author**~~**ence to:**~~

~~Fatemeh Farshadpour, Ph.D., Department of Virology, School of Medicine, Bushehr University of Medical Sciences 751463334, Moallem Street, Bushehr, Iran~~

~~Tel: +98 9171712653~~

Email: f.farshadpour@bpums.ac.ir (FF)

Reza Taherkhani and Fatemeh Farshadpour contributed equally to this work.

Abstract

Background: Considering perinatal transmission and the high rate of chronic hepatitis B virus (HBV) infection in infants, diagnosis of HBV infection during pregnancy and timely interventions are of great importance. Therefore, this study was performed to investigate the prevalence and genotype distribution of HBV infection and associated risk factors among pregnant women in the northern shores of the Persian Gulf, South of Iran.

Methods: Serum samples of 1425 pregnant women were tested for the presence of HBsAg and HBcAb by ELISA assay (HBsAg one – Version ULTRA and HBc Ab ELISA kits, DIA.PRO, Milan, Italy). The seropositive samples were tested for the presence~~detection~~ of HBV DNA by nested PCR, targeting S, X,~~ and~~ pre-core (pre-C) and basal core promoter (BCP) regions of the HBV genome. The amplified fragments were sequenced by Sanger dideoxy sequencing technology to evaluate the genotype distribution and mutations of HBV infection by using the MEGA 7 software.~~Positive HBV DNA samples were sequenced to evaluate the genotype distribution and mutations of HBV infection.~~

Results: Of 1425 participants, 15 pregnant women (1.05%, 95% CI: 0.64%-1.73%) were positive for HBsAg,~~ and~~ 41 women (2.88%, 95% CI: 2.10%-3.88%) were positive for~~had~~ HBcAb,~~.~~and 5 women (0.35%, 95% CI: 0.15% –0.82%) had HBV viremia with genotype D, sub-genotype D3 and subtype ayw2. One of the viremic samples was positive for HBcAb but negative for HBsAg, which is indicative of an occult HBV infection. ~~-~~HBsAg seroprevalence was higher among pregnant women aged 20 to 29 years, women in the third trimester of pregnancy, residents of Khormuj city, Afghan immigrants, illiterate~~uneducated~~ women, and pregnant women with the history of tattoo and HBV vaccination. The highest rate of HBcAb seroprevalence was observed in residents of Borazjan city, Turk ethnicity, the age group >39 years, and those women with more parities and a history of abortion. Nevertheless, HBV seroprevalence among pregnant women was not statistically associated with these variables. In contrast, HBcAb seropositivity was significantly associated with the history of tattoo~~, so that tattooing was a risk factor for exposure to HBV infection~~ (P = 0.018). ~~According to the molecular evaluation, 26.7% of HBsAg seropositive pregnant women and 2.7% of HBcAb seropositive women had HBV viremia with genotype D, sub-genotype D3 and subtype ayw2. One of the viremic samples was positive for HBcAb but negative for HBsAg, which is indicative~~

Formatted: Font: Times New Roman, 18 pt

Formatted: Heading 1, Left, Line spacing: single

Formatted: Font: Not Bold

of an occult HBV infection. According to mutations analyses, seven amino acid substitutions in the HBsAg, one point mutation in the pre-C region, and five points mutations in the BCP region were detected. Besides, the BCP mutations caused amino acid substitutions in the X protein.Moreover, several amino acid substitutions and points mutations were detected in the S, pre-core and basal core promoter regions of the HBV genome.

Conclusion: These results indicate a relatively low prevalence of HBV infection among pregnant women in the South of Iran, while tattooing is a risk factor for exposure to HBV infection. Moreover, all of the HBV-positive pregnant women were asymptomatic and unaware of their infection. Therefore, routine screening for HBV markers during pregnancyConsidering the high rate of chronic HBV carriers among those patients who acquire HBV infection perinatally from infected mothers, screening of women of childbearing age, appropriate treatment of HBV-infected women, and HBV vaccination are recommended to decrease mother-to-child transmission of HBV.

Keywords: Hepatitis B virus; Pregnancy; Risk factor; Prevalence; Genotype; Mutation; Iran

Introduction

Hepatitis B virus (HBV), a member of the family *Hepadnaviridae*, is characterized by asymptomatic, acute, or chronic hepatitis, which might progress to fulminant hepatic failure, cirrhosis, or hepatocellular carcinoma [1, 2]. Currently, about 250 million of the world population are infected with chronic hepatitis B, and 65 million of those infected are women of childbearing age. HBV-related liver diseases are responsible for approximately 8,00 ,000 deaths annually [3].

The HBV genome is a circular partially double-stranded DNA with four overlapping open reading frames: S ORF for surface proteins (HBsAg), P ORF for polymerase (reverse transcriptase), preC/*C* ORF for e antigen (HBeAg) and core protein (HBcAg), and X ORF for X protein [4-6]. Due to the lack of proofreading function of HBV reverse transcriptase, a high rate of mutations naturally occurs during the replication of the viral genome. The most common

Formatted

Formatted: Font: Times New Roman, 18 pt

Formatted: Heading 1, Line spacing: single

mutations are pre-core (pre-C) and basal core promoter (BCP) mutations in the HBV core gene [5, 7]. These mutations affect the expression of HBV e antigen (HBeAg) protein and have a significant association with progressive liver diseases such as liver cirrhosis and HCC [2, 8]. In addition, some mutations in the S region have an important role in the occurrence of occult HBV infection with undetectable levels of HBsAg [9].

Horizontal and perinatal transmissions are the main routes of HBV infection. Horizontal transmission might happen through blood transfusion, intravenous drug use, surgery, tattooing, and sexual intercourse [10, 11]. Perinatal or mother-to-child transmission is one of the most common modes of HBV transmission worldwide. Perinatal transmission of HBV most often occurs during birth and delivery [12, 13]. The risk of HBV infection in children born to mothers who are HBsAg-positive and HBeAg-positive is 70% to 90%, and over 90% of these children eventually develop chronic HBV infection, with approximately 25% progressing to cirrhosis and hepatocellular carcinoma [13-15]. This high rate of chronic HBV infection indicates a worse prognosis in infants. However, diagnosis of HBV infection during pregnancy or before delivery and timely interventions can mitigate the risk of HBV transmission to fetus or newborn and, consequently, reduce the disease burden in the community [3, 16].

Iran, with a prevalence rate of 3% in the general population, is considered one of the intermediate endemic countries for HBV infection [17]. The initiation of HBV vaccination of infants since 1993 and teenagers since 2006 in the country has had a significant role in reducing the prevalence of HBV infection in the community [18]. C~~More importantly, c~~onsidering the high rate of chronic HBV carriers among those patients who acquire HBV infection perinatally from infected mothers, perinatal transmission is probably the dominant route of HBV infection transmission in Iran [19]. Therefore, the prevention of maternal infection and its transmission to

Formatted: Highlight

the fetuses or newborns is essential, so the diagnosis of infected pregnant women is of particular importance. Nevertheless, no report is available on the prevalence of HBV infection among the pregnant population in the South of Iran, and despite the profound effects of HBV genotypes and mutants on the progression and treatment of HBV infection, little is known about the molecular analysis of HBV infection in Iran. Therefore, this study was performed to investigate the prevalence, risk factors, and genotype distribution of HBV infection and associated mutations in the S, X, pre-C, and BCP regions of the HBV genome among pregnant women resident in the northern shores of the Persian Gulf, South of Iran.

Subjects and ~~Methods~~ methods

Study setting and population

The study was conducted from January 2018 to June 2019 and included 1425 pregnant women from the public health centers in five cities of the northern shores of the Persian Gulf. The public health centers were chosen according to the multi-stage cluster sampling method. In the first stage, Bushehr, Ahram, Borazjan, Jam, and Khormuj cities of this territory were selected randomly. In the second stage, three public health centers from each city were selected randomly, and all the pregnant women attending these centers for routine visits were included consecutively in this study. The leftover serum samples of pregnant women were used for HBV detection. The pregnant women gave written informed consent to participate in the study and use their serum samples for HBV detection. The socio-demographic characteristics, pregnancy information, history of HBV vaccination, ~~and~~ the potential risk factors associated with HBV infection, and levels of liver enzymes were obtained by interviewing each pregnant woman using a questionnaire and from the clinical records of the participants at the public health centers. The

Formatted: Font: Times New Roman, 18 pt

Formatted: Heading 1, Left, Line spacing: single

Formatted: Font: Times New Roman, 16 pt, Not Italic

Formatted: Highlight

Formatted: Highlight

Formatted: Highlight

Ethics Committee of the Bushehr University of Medical Sciences approved this study with reference number IR.BPUMS.REC.1396.111, and the Deputy Research and Affairs of the University funded this study with grant number 357.

Laboratory methods Laboratory diagnosis

HBV serological testing

Serum samples of 1425 pregnant women were tested manually for the presence of hepatitis B surface antigen (HBsAg) and hepatitis B core antibody (HBcAb) using commercially available ELISA kits (HBsAg one – Version ULTRA and HBc Ab ELISA kits, DIA.PRO, Milan, Italy) according to the manufacturer's instructions. The specificity and sensitivity of these kits were 100%. The HBV seropositive pregnant women were tested for HCV and HIV coinfections by ELISA kits (HCV Ab and HIV Ab/Ag ELISA kits, DIA.PRO, Milan, Italy).

PCR amplification and sequencing

The seropositive samples were tested to evaluate the genotype distribution and mutations of HBV infection by two nested PCR assays, targeting S, X, and pre-C and BCP regions of the HBV genome [20-24]. Following the extraction of HBV DNA using the High Pure Viral Nucleic Acid kit (Roche, Mannheim, Germany), part of the S region was amplified in the first round PCR using primers 244-HBS-F1 (GAGTCTAGACTCGTGGTGGACTTC) and 691-HBS-R1 (AAATKGCACTAGTAAACTGAGCCA). The second-round PCR was performed using primers 255-HBS-F2 (CGTGGTGGACTTCTCTCAATTTTC) and 671-HBS-R2 (GCCARGAGAAACGGRCTGAGGCCC). The 416 bp length fragments from the S region were sequenced by Sanger dideoxy sequencing technology to determine HBV genotypes (Macrogen Co., Korea).

Formatted: Heading 2 Char, Font: 16 pt, Not Bold

Formatted: Font: Times New Roman, 14 pt

Formatted: Heading 3, Left, Line spacing: single

Formatted: Highlight

Formatted: Highlight

Formatted: Not Highlight

Formatted: Not Highlight

Formatted: Not Highlight

Formatted: Highlight

Formatted: Highlight

Formatted: Highlight

Formatted: Highlight

Formatted: Highlight

Formatted: Highlight

Formatted: Highlight

Formatted: Font: Times New Roman, 14 pt

Formatted: Heading 3, Left, Line spacing: single

Formatted: Highlight

Formatted: Highlight

Phylogenetic and mutational analysis

The obtained sequences were aligned, and compared with the reference sequences representing the standard HBV genotypes available at the nucleotide database of the NCBI by ClustalW program in the Molecular Evolutionary Genetics Analysis (MEGA)MEGA software version 7.0 (Biodesign Institute, Tempe, AZ, USA), and the phylogenetic tree was constructed by the neighbor-joining method, as described previously [25].

To determine mutations in BCP and pre-C regions, 789 nucleotides and 740 nucleotides of the X and pre-C region were amplified by nested PCR using outer primers [forward primer (1606-pre-C-F1): GCATGGAGACCACCGTGAAC; reverse primer (2395-pre-C-R1): AGGCGAGGGAGTTCTTCTTC] and inner primers [forward primer (1653-pre-C-F2): CATAAGAGGACTCTTGGACT; reverse primer (2393-pre-C-R2): GCGAGGGAGTTCTTCTTC], respectively. The amplified 740 bp length fragments were sequenced, aligned, and compared with the reference sequences of X and pre-C region of HBV by the ClustalW program in the MEGA 7 software. The S, X, and pre-C sequences isolated from pregnant women were submitted to the GenBank sequence database. The sequences of primers and regions in the HBV genome for detection of HBV genotypes and mutants are summarized in table Table 1.

Table 1. Sequences of primers for amplification of S, X, and pre-core regions of HBV genome.

Virus	Primers Name	Sequences of Primers 5′→3′	Gene	Region in Genome	Annealing temperature	Size	References
HBV	244-HBS-F1	GAGTCTAGACTCGTGGTGGACTTC	S	244-267	56 °CC	447 bp	[20, 21]
	691-HBS-R1	AAATKGCACTAGTAAACTGAGCCA		668-691			

Formatted: Font: Times New Roman, 14 pt

Formatted: Heading 3, Left, Line spacing: single

Formatted: Font: Bold

Formatted: Font: Bold

	255-HBS-F2	CGTGGTGGACTTCTCTCAATTTTC		255-278	56 °C	416 bp	
	671-HBS-R2	GCCARGAGAAACGGRCTGAGGCCC		648-671			
HBV	1606-pre-C-F1	GCATGGAGACCACCGTGAAC	X and pre-core region	1606-1625	57 °C	789 bp	[22-24]
	2395-pre-C-R1	AGGCGAGGGAGTTCTTCTTC		2376-2395			
	1653-pre-C-F2	CATAAGAGGACTCTTGGACT		1653-1672	55 °C	740 bp	
	2393-pre-C-R2	GCGAGGGAGTTCTTCTTC		2376-2393			

Statistical analysis

The χ^2 test or Fisher's exact test was used to analyze and compare categorical variables between HBV-positive and HBV-negative pregnant women. Quantitative variables were compared by the Student's *t*-test. Univariate and multivariate ~~Logistic~~ logistic regression analys~~i~~es ~~was used~~were performed to evaluate the risk factors associated with HBV infection among pregnant women. The data were analyzed by SPSS 17 package program (SPSS Inc., Chicago, IL, USA), and *P* values < 0.05 were defined statistically significant.

Results

Of 1425 pregnant women, 616 women were from Bushehr city, 207 were from Ahram city, 40 were from Khormuj city, 440 were from Borazjan city and 122 were from Jam city. The mean age ± SD of pregnant women was 28.1±5.99 years with a range of 14-46 years. The majority of pregnant women were in the third trimester of pregnancy (68.4%), the age group 20–29 years (55.2%), educated (97.1%), and Fars (90.0%). Of 1425 participants, 15 pregnant women (1.05%, 95% CI: 0.64%-1.73%) were positive for HBsAg, and 41 women (2.88%, 95% CI: 2.10%-3.88%) had HBcAb. Moreover, 4 of 41 HBcAb seropositive samples (9.75%) were also positive for HBsAg.

HBsAg seropositive pregnant women had a lower mean age (27.53±5.96) compared to HBsAg seronegative pregnant women (28.11±5.99), while this difference was not statistically significant

Formatted: Font: Times New Roman, 16 pt, Not Italic

Formatted: Heading 2, Left, Line spacing: single

Formatted: Font: Times New Roman, 18 pt

Formatted: Heading 1, Line spacing: single

(*P* = 0.71). Moreover, there was no difference between the mean age of HBcAb seropositive pregnant women (28.88±5.96) and HBcAb seronegative pregnant women (28.08±5.99) (*P* = 0.402). The majority of HBsAg seropositive women were in the age group 20-29 years (1.4%), the third trimester of pregnancy (1.3%), residents in Khormuj city (2.5%), Afghan (1.61%), illiterateuneducated (2.4%) and had a history of tattoo (1.6%) and HBV vaccination (1.6%) (S1 Table). The highest rate of HBcAb seroprevalence was observed in residents of Borazjan city (3.9%), Turk and Afghan immigrants (8.3% and 6.5%, respectively), the age group >39 years (4.3%), illiterateuneducated pregnant women (7.1%), pregnant women in the second trimester of pregnancy (3.6%), and those women with more parities (5.0%) and a history of abortion (3.0%) (S2 Table). However, none of these variables were statistically associated with HBsAg and HBcAb seroprevalence. In contrast, HBcAb seropositivity was significantly associated with history of tattoo, so that tattooing was a risk factor for exposure to HBV infection (OR: 1.47; 95% CI: 1.06-2.04; *P* = 0.018) (S2 Table). In addition, HBcAb seropositivity was more prevalent in those samples collected in October (11.5%) compared to the other months. However, according to the multivariate logistic regression analysis, the difference in the monthly rate of HBcAb seroprevalence was statistically insignificant. All of the HBV seropositive samples had normal levels of liver enzymes and were negative for HCV and HIV. The prevalence of HBsAg and HBcAb among pregnant women grouped according to socio-demographic characteristics and qualitative variables are presented in tables 2 and 3S1 and S2 Tables, respectively.

~~**Table 2.** Prevalence of HBsAg according to socio-demographic and qualitative variables among pregnant women in South of Iran~~

	**No. of all participants (%):1425 (100%)**	**No. of HBsAg negative subjects (%):1410 (98.9%)**	**No. of HBsAg positive subjects (%):15 (1.05%)**	**P-Value**
**Age groups (years)**				**0.425**
<20	108 (7.6%)	108 (100.0%)	0 (0.0%)	
20-29	786 (55.2%)	775 (98.6%)	11 (1.4%)	

30-39	485 (34.0%)	481 (99.2%)	4 (0.8%)	
>39	46 (3.2%)	46 (100.0%)	0 (0.0%)	
**Place of residence (city)**				**0.292**
Bushehr	616 (43.2%)	612 (99.4%)	4 (0.6%)	
Borazjan	440 (30.9%)	432 (98.2%)	8 (1.8%)	
Ahram	207 (14.5%)	206 (99.5%)	1 (0.5%)	
Jam	122 (8.6%)	121 (99.2%)	1 (0.8%)	
Khormoj	40 (2.8%)	39 (97.5%)	1 (2.5%)	
**Ethnicity**				**0.788**
Fars	1283 (90.0%)	1269 (98.9%)	14 (1.1%)	
Arab	68 (4.8%)	68 (100.0%)	0 (0.0%)	
Afghan	62 (4.4%)	61 (98.4%)	1 (1.6%)	
Turk	12 (0.8%)	12 (100.0%)	0 (0.0%)	
**Stage of gestation**				**0.308**
First trimester	256 (18.0%)	255 (99.6%)	1 (0.4%)	
Second trimester	194 (13.6%)	193 (99.5%)	1 (0.5%)	
Third trimester	975 (68.4%)	962 (98.7%)	13 (1.3%)	
**Number of Pregnancies**				**0.711**
One pregnancy	440 (30.9%)	435 (98.9%)	5 (1.1%)	
Two pregnancies	785 (55.1%)	776 (98.9%)	9 (1.1%)	
Three and more than three pregnancies	200 (14.0%)	199 (99.5%)	1 (0.5%)	
**History of Abortion**				**0.948**
No	934 (65.5%)	924 (98.9%)	10 (1.1%)	
Yes	231 (16.2%)	229 (99.1%)	2 (0.9%)	
Unknown	260 (18.2%)	257 (98.8%)	3 (1.2%)	
**Education**				**0.535**
Upper diploma	366 (25.7%)	364 (99.5%)	2 (0.5%)	
Diploma	677 (47.5%)	670 (99.0%)	7 (1.0%)	
Under diploma	340 (23.9%)	335 (98.5%)	5 (1.5%)	
Uneducated	42 (2.9%)	41 (97.6%)	1 (2.4%)	
**Year**				**1.0**
2018	797 (55.9%)	789 (99.0%)	8 (1.0%)	
2019	628 (44.1%)	621 (98.9%)	7 (1.1%)	
**Month**				**0.505**
Oct	113 (7.9%)	112 (99.1%)	1 (0.9%)	
Nov	102 (7.2%)	100 (98.0%)	2 (2.0%)	
Dec	112 (7.9%)	111 (99.1%)	1 (0.9%)	
Jan	108 (7.6%)	106 (98.1%)	2 (1.9%)	
Feb	291 (20.4%)	288 (99.0%)	3 (1.0%)	
Mar	248 (17.4%)	247 (99.6%)	1 (0.4%)	
Apr	131 (9.2%)	130 (99.2%)	1 (0.8%)	
May	168 (11.8%)	168 (100.0%)	0 (0.0%)	
June	123 (8.6%)	120 (97.6%)	3 (2.4%)	
July	29 (2.0%)	28 (96.6%)	1 (3.4%)	
**Smoking**				**0.572**
No	836 (58.7%)	828 (99.0%)	8 (1.0%)	
Yes	62 (4.4%)	62 (100.0%)	0 (0.0%)	
Unknown	527 (37.0%)	520 (98.7%)	7 (1.3%)	
**History of blood transfusion**				**0.784**
No	892 (62.6%)	883 (99.0%)	9 (1.0%)	
Yes	18 (1.3%)	18 (100.0%)	0 (0.0%)	

Unknown	515 (36.1%)	509 (98.8%)	6 (1.2%)	
**History of surgery**				**0.946**
No	692 (48.6%)	685 (99.0%)	7 (1.0%)	
Yes	218 (15.3%)	216 (99.1%)	2 (0.9%)	
Unknown	515 (36.1%)	509 (98.8%)	6 (1.2%)	
**History of tattoo**				**0.728**
No	786 (55.2%)	779 (99.1%)	7 (0.9%)	
Yes	124 (8.7%)	122 (98.4%)	2 (1.6%)	
Unknown	515 (36.1%)	509 (98.8%)	6 (1.2%)	
**History of dentistry**				**0.137**
No	504 (35.4%)	496 (98.4%)	8 (1.6%)	
Yes	406 (28.5%)	405 (99.8%)	1 (0.2%)	
Unknown	515 (36.1%)	509 (98.8 %)	6 (1.2%)	
**History of HBV vaccination**				**0.433**
No	314 (22.0%)	312 (98.7%)	4 (1.3%)	
Yes	314 (22.0%)	309 (98.4%)	5 (1.6%)	
Unknown	797 (55.9%)	789 (99.2%)	6 (0.8%)	

Table 3. Prevalence of HBcAb according to socio-demographic and qualitative variables among pregnant women in South of Iran

	**No. of all participants (%):1425 (100%)**	**No. of HBcAb negative subjects (%):1384 (97.12%)**	**No. of HBcAb positive subjects (%):41 (2.88%)**	**P-Value**
**Age groups (years)**				**0.835**
<20	108 (7.6%)	106 (98.1%)	2 (1.9%)	
20-29	786 (55.2%)	764 (97.2%)	22 (2.8%)	
30-39	485 (34.0%)	470 (96.9%)	15 (3.1%)	
>39	46 (3.2%)	44 (95.7%)	2 (4.3%)	
**Place of residence (city)**				**0.497**
Bushehr	616 (43.2%)	601 (97.6%)	15 (2.4%)	
Borazjan	440 (30.9%)	423 (96.1%)	17 (3.9%)	
Ahram	207 (14.5%)	202 (97.6%)	5 (2.4%)	
Jam	122 (8.6%)	118 (96.7%)	4 (3.3%)	
Khormoj	40 (2.8%)	40 (100.0%)	0 (0.0%)	
**Ethnicity**				**0.195**
Fars	1283 (90.0%)	1248 (97.3%)	35 (2.7%)	
Arab	68 (4.8%)	67 (98.5%)	1 (1.5%)	
Afghan	62 (4.4%)	58 (93.5%)	4 (6.5%)	
Turk	12 (0.8%)	11 (91.7%)	1 (8.3%)	
**Stage of gestation**				**0.748**
First trimester	256 (18.0%)	248 (96.9%)	8 (3.1%)	
Second trimester	194 (13.6%)	187 (96.4%)	7 (3.6%)	
Third trimester	975 (68.4%)	949 (97.3%)	26 (2.7%)	
**Number of Pregnancies**				**0.091**
One pregnancy	440 (30.9%)	426 (96.8%)	14 (3.2%)	
Two pregnancies	785 (55.1%)	768 (97.8%)	17 (2.2%)	
Three and more than three pregnancies	200 (14.0%)	190 (95.0%)	10 (5.0%)	

**History of Abortion**				**0.974**
No	934 (65.5%)	907 (97.1%)	27 (2.9%)	
Yes	231 (16.2%)	224 (97.0%)	7 (3.0%)	
Unknown	260 (18.2%)	253 (97.3%)	7 (2.7%)	
**Education**				**0.226**
Upper diploma	366 (25.7%)	359 (98.1%)	7 (1.9%)	
Diploma	677 (47.5%)	655 (96.8%)	22 (3.2%)	
Under diploma	340 (23.9%)	331 (97.4%)	9 (2.6%)	
Uneducated	42 (2.9%)	39 (92.9%)	3 (7.1%)	
**Year**				**0.113**
2018	797 (55.9%)	769 (96.5%)	28 (3.5%)	
2019	628 (44.1%)	615 (97.9%)	13 (2.1%)	
**Month**				**0.001**
Oct	113 (7.9%)	100 (88.5%)	13 (11.5%)	
Nov	102 (7.2%)	100 (98.0%)	2 (2.0%)	
Dec	112 (7.9%)	107 (95.5%)	5 (4.5%)	
Jan	108 (7.6%)	107 (99.1%)	1 (0.9%)	
Feb	291 (20.4%)	288 (99.0%)	3 (1.0%)	
Mar	248 (17.4%)	244 (98.4%)	4 (1.6%)	
Apr	131 (9.2%)	128 (97.7%)	3 (2.3%)	
May	168 (11.8%)	163 (97.0%)	5 (3.0%)	
June	123 (8.6%)	120 (97.6%)	3 (2.4%)	
July	29 (2.0%)	27 (93.1%)	2 (6.9%)	
**Smoking**				**0.195**
No	836 (58.7%)	807 (96.5%)	29 (3.5%)	
Yes	62 (4.4%)	62 (100.0%)	0 (0.0%)	
Unknown	527 (37.0%)	515 (97.7%)	12 (2.3%)	
**History of blood transfusion**				**0.195**
No	892 (62.6%)	871 (97.6%)	21 (2.4%)	
Yes	18 (1.3%)	18 (100.0%)	0 (0.0%)	
Unknown	515 (36.1%)	495 (96.1%)	20 (3.9%)	
**History of surgery**				**0.21**
No	692 (48.6%)	677 (97.8%)	15 (2.2%)	
Yes	218 (15.3%)	212 (97.2%)	6 (2.8%)	
Unknown	515 (36.1%)	495 (96.1%)	20 (3.9%)	
**History of tattoo**				**0.018**
No	786 (55.2%)	772 (98.2%)	14 (1.8%)	
Yes	124 (8.7%)	117 (94.4%)	7 (5.6%)	
Unknown	515 (36.1%)	495 (96.1%)	20 (3.9%)	
**History of dentistry**				**0.148**
No	504 (35.4%)	490 (97.2%)	14 (2.8%)	
Yes	406 (28.5%)	399 (98.3%)	7 (1.7%)	
Unknown	515 (36.1%)	495 (96.1%)	20 (3.9%)	
**History of HBV vaccination**				**0.370**
No	314 (22.0%)	310 (97.5%)	8 (2.5%)	
Yes	314 (22.0%)	308 (98.1%)	6 (1.9%)	
Unknown	797 (55.9%)	766 (96.6%)	27 (3.4%)	

According to the molecular ~~evaluation~~testing, 5 women (0.35%, 95% CI: 0.15% – 0.82%) had HBV viremia. Of viremic samples, ~~HBV DNA was found in 0.35% (95% CI: 0.15% - 0.82%) of samples (5/1425), of which~~ 3 samples were positive for HBsAg, 1 sample was positive for both HBsAg and HBcAb, and 1 sample was positive for HBcAb but negative for HBsAg, which is indicative of an occult HBV infection. Notably, 3 samples were positive in the first round of nested PCR, and 2 samples were found to be positive in the second round of nested PCR (Figs~~.~~ 1 and 2). Overall, 26.7% of HBsAg seropositive pregnant women and 2.7% of HBcAb seropositive women had HBV viremia with genotype D, sub-genotype D3, and subtype ayw2 (GenBank accession Nos. OK490614-OK490616) (Fig~~.~~ 3). Seven amino acid substitutions were detected in HBsAg of strains isolated from pregnant women, including the conversion of Ile → Leu at amino acid 110 (I110L), Thr → Pro at amino acid 127 (T127P), Asp → Glu at amino acid 144 (D144E), Ser → Ala at amino acid 154 (S154A), Gly → Ala at amino acid 159 (G159A), Ala → Gly at amino acid 166 (A166G) and Ala → Val at amino acid 168 (A168V) of HBsAg (S1 Fig~~Supplementary Fig. 1~~ and Table ~~2~~4). Moreover, a G to A substitution at nucleotide position 1896 (G1896A) was detected in the pre-C region (~~Supplementary Fig. 2~~S2 Fig). T1753A, A1761C, G1764T/A, C1766G/T, and C1773T mutations were detected in the BCP region. Of these, T1753A, A1761C and G1764A mutations cause the conversion of Ile → Asn at amino acid 127 (I127N), Lys → Gln at amino acid 130 (K130Q), and Val → Leu at amino acid 131 (V131L) of the X protein, respectively (~~Supplementary Fig. 3~~S3 Fig).

Table ~~2~~4. Frequency of point replacement mutations in HBsAg of strains isolated from pregnant women in the South of Iran.

Position S mutation	Frequency of point mutation No. (%)	Patient No.
I110L	1 (33%)	HB55
T127P	2 (66%)	HB51, HB55
D144E	1 (33%)	HB35
S154A	1 (33%)	HB55
G159A	1 (33%)	HB55

Formatted: Highlight

Formatted: Font: Bold

Formatted: Font: Bold

Formatted: Font: Bold

A166G	1 (33%)	HB35
A168V	2 (66%)	HB51, HB55

Fig 1. Electrophoresis of PCR products of S region of HBV genome extracted from serum samples of pregnant women on 2% agarose gel. L, 100-bp DNA ladder; N, negative control; P, positive control; 1, 2, 3, 4, 6, 7 and 9, amplified products (≈417 bp).

Fig 2. Electrophoresis of PCR products of X and pre-core regions of HBV genome extracted from serum samples of pregnant women on 2% agarose gel. L, 100-bp DNA ladder; Neg, negative control; Pos, positive control; Sam, amplified product (≈740 bp).

Fig 3. Neighbor-joining phylogenetic tree based on ~380 bp nucleotide sequence (~270-670 bp of the complete reference genome) of S region of HBV isolates (green mark) from the serum samples of pregnant women in South of Iran (GenBank accession Nos. OK490614-OK490616). Bootstrap re-sampling strategy and reconstruction were carried out 1,000 times to confirm the reliability of the phylogenetic tree.

~~**Supplementary Fig 1.** Alignment of the amino acid sequences of HBsAg (44 aa to 171 aa) of strains isolated from pregnant women (GenBank accession Nos. OK490614-OK490616) and the reference sequences available at the GenBank~~

~~**Supplementary Fig 2.** Alignment of the amino acid sequences of pre-core region isolated from pregnant women (GenBank accession Nos. OK490617 and OK490618) and the reference sequences available at the GenBank~~

~~**Supplementary Fig 3.** Alignment of the amino acid sequences (109 aa to 154 aa) of X protein (1698-1838 bp nucleotide sequence) isolated from pregnant women (GenBank accession Nos. OK490617 and OK490618) and the reference sequences available at the GenBank~~

Discussion

HBV causes chronic infections in 2% to 3% of adults and 95% of infants and is an important factor in the development of advanced chronic liver disease and hepatocellular carcinoma in these patients [3]. Notably, perinatal or mother-to-infant transmission is responsible for the occurrence of over 50% of chronic HBV carriers [13]. Considering the high rates of perinatal transmission and chronic HBV infection in infants, diagnosis of HBV infection among pregnant

Formatted: Font: Bold
Formatted: Font: Bold
Formatted: Font: Bold
Formatted: Font: Bold
Formatted: Font: Bold
Formatted: Font: Bold
Formatted: Font: Bold
Formatted: Font: Bold
Formatted: Font: Bold, Highlight
Formatted: Font: Bold
Formatted: Font: 18 pt
Formatted: Heading 1, Left, Line spacing: single

women is of great importance. Despite this importance, there is no report on the prevalence of HBV infection among pregnant women in the northern shores of the Persian Gulf, South of Iran. Therefore, we evaluated the prevalence and molecular epidemiology of HBV infection among pregnant women in this region and found HBV prevalence of 1.05% for HBsAg, 2.88% for HBcAb and 0.35% for HBV viremia with genotype D, sub-genotype D3 and subtype ayw2.

The HBsAg seroprevalence of 1.05% observed in pregnant women is considerably higher than the HBsAg prevalence of 0.15% reported in the blood donors of this region [18]. Besides, the HBV prevalence noted in this study is slightly lower than the overall prevalence of HBV among Iranian pregnant women (1.18%) [15]. In this study, all of the HBV seropositive pregnant women were asymptomatic and unaware of their infection. These infected but asymptomatic pregnant women may remain undiagnosed over time. The risk of HBV infection in children born to infected mothers is 70% to 90%, and over 90% of these children eventually develop chronic HBV infection, with approximately 25% progressing to cirrhosis and hepatocellular carcinoma [13-15]. This high rate of chronic HBV infection indicates a worse prognosis in infants. Routine screening for HBV infection during pregnancy is therefore recommended for the identification of HBV-infected pregnant women and neonates born at risk. ~~Since m~~Maternal treatment with oral antivirals such as tenofovir, telbivudine, and lamivudine~~, HBV vaccination~~ and immunoglobulin administration at birth can ~~decrease the risk of neonatal infection and~~ prevent mother-to-child transmission of HBV. ~~and, consequently~~Consequently, this reduces the burden of chronic HBV infection in the community [3, 16]. According to the results of this study, 0.35% of pregnant women had HBV viremia. ~~Given that p~~Prenatal transmission, which is leading to postpartum immunoprophylaxis incompetence, is possible in women with HBV viremia [14].~~,~~ ~~and considering the fact that~~Since, a significant percentage of pregnant women ~~in South of Iran~~

Formatted: Highlight

Formatted: Highlight

Formatted: Highlight

Formatted: Highlight

Formatted: Highlight

were~~are~~ not vaccinated~~ and are at risk of HBV infection during pregnancy; therefore~~, HBV vaccination ~~and screening of women before pregnancy ~~is~~are also~~ recommended to reduce the incidence of HBV infection during pregnancy.

The prevalence of 1.05% for HBsAg observed in the present study is higher than those observed among pregnant women in other parts of Iran, 0.18% in Babol (North of Iran) [26], 0.3% in Mashhad (North-East of Iran) [27], 0.59% in Dehloran (West of Iran) [28], 0.7% in Lorestan (West of Iran) [29], 0.77% in Kurdistan (West of Iran) [30] and 0.87% in East Azerbaijan (North-West of Iran) [30] but lower than 1.3% in Rafsanjan (South-East of Iran) [31], 1.93% in Golestan (North of Iran) [30], 2.26% in Jiroft (South-East of Iran) [30] and 2.5% in Malekan (North-West of Iran) [32]. The HBsAg prevalence noted in this study is also higher than those observed among pregnant women of some other countries, 0.9% in Brazil [33] ~~and 1.01% in South India [34] ~~but lower than those reported among pregnant women in Ghana (3.3%) [35], Central Nigeria~~–~~ (5.5%) [36], Shenyang, China (5.~~49~~5%) [37], Ethiopia (7.9%) [38], Gambia (9.2~~0~~%) [39], North Cameroon (10.2%) [40] and Yemen (10.8%) [41]. Moreover, the prevalence of 2.88% for HBcAb reported in this study is higher than that observed among pregnant women in Malekan (1.25%) [32] but lower than that in Lorestan (3.4%) [29]. This prevalence is lower than those of other countries such as Brazil (7.4%) [33] and Shenyang, China (29.65%) [37]. These variations in the seroprevalence of HBV are probably because of differences in rates of high-risk behaviors, level of awareness, immunization status, the routes of transmission, epidemiology of HBV, and burden of the infection in different regions. However, differences in sociodemographic characteristics of the participants and the sensitivity and specificity of the diagnosis assays~~ELISA kits~~ in different studies can also explain these variations.

In this study, HBV seroprevalence among the pregnant women was not statistically associated with age, stage of gestation, number of pregnancies, place of residency, level of education, ethnicity, and history of blood transfusion, abortion, dentistry, surgery and HBV vaccination, although the majority of HBsAg seropositive pregnant women were in the age group 20-29 years, the third trimester of pregnancy, residents in Khormuj city, illiterate~~uneducated~~, Afghan and had a history of tattoo and HBV vaccination. Furthermore, the highest rate of HBcAb seroprevalence was observed in residents of Borazjan city, Turk and Afghan immigrants, the age group >39 years, illiterate~~uneducated~~ pregnant women, pregnant women in the second trimester of pregnancy, and those women with more parities and a history of abortion. The higher seroprevalence of HBV among pregnant women resident in Khormuj and Borazjan cities might be due to the higher prevalence of HBV infection in the general population of these cities. In contrast, HBcAb seropositivity was significantly associated with the time of sampling and history of tattoo, so that tattooing was a risk factor for exposure to HBV infection. In addition, HBcAb seropositivity was more prevalent in those samples collected in October compared to the other months. The reason for this monthly pattern of HBcAb seroprevalence is not clear.

A recent meta-analysis study revealed that HBsAg seroprevalence among pregnant women in Iran is associated with the history of blood transfusion, abortion, illiteracy, and intravenous drug use by spouses~~–~~ [15]. In a study from the South-East of Iran, HBsAg seroprevalence among pregnant women was associated with illicit drug use and tattooing [31]. In another study from the West of Iran, more parities and abortion were risk factors for exposure to HBV [28]. In a recent study from Yemen, a significant association was found between female circumcision and HBsAg seropositivity [41]. Another study from North Cameroon reported a significant association between HBV infection and a history of blood transfusion [40]. Previous studies from Ethiopia

demonstrated that HBsAg seroprevalence among pregnant women is associated with multiple sexual partners, history of abortion, dentistry, and surgery [38, 42]. This discrepancy between reports might be due to differences in the predominant routes of transmission and risk behavior patterns in different geographical regions.

In the present study, the highest rate of HBsAg seroprevalence was observed in pregnant women aged 20 to 29 years (1.4%). Considering the compulsory vaccination of infants since 1993 and teenagers since 2006 [18], this is probably due to the partial ineffectiveness or incompleteness of the national HBV vaccination program in this age group. However, further studies are required to determine the efficacy of hepatitis B vaccination among pregnant women in this region. our results indicate that vaccination has not been able to prevent HBV infection in this age group or implementation of the national HBV vaccination program has not been completely effective. Moreover, considering that there was no difference between HBV-vaccinated and HBV-unvaccinated pregnant women regarding the prevalence of HBsAg, it seems that a study on the effectiveness of the national vaccination program is needed. Therefore, although there are effective tools and strategies for the prevention and treatment of HBV infection, management of HBV infection during pregnancy faces challenges that need further investigation. On the other hand, all pregnant women under 20 years old were negative for HBsAg, which is probably due to the initiation of neonatal vaccination in the country in 1993, awareness of HBV infection among youths, and less exposure to HBV or less engagement in risky behaviors in young ages. In contrast, a recent study in Central Nigeria demonstrated a higher prevalence of HBsAg among pregnant women under 20 years old [36].

In this study, occult HBV infection was detected in one sample with undetectable levels of HBsAg and low HBV DNA levels. Nevertheless, P120T and G145R mutations associated with

Formatted: Highlight

Formatted: Highlight

failure of HBsAg detection were not found in this sample. P120T and G145R mutations in the S gene ~~were reported to result in~~are associated with low responses to HBV vaccination and immunoglobulin therapy as well as failures of diagnostic tests [43, 44]. ~~This sample was found to be positive in the second round of PCR.~~ In this study, one point mutation in the pre-C region and five points mutations in the BCP region were detected. Of these, the pre-C mutation (G1896A) eliminates HBeAg expression at the translational level, whereas BCP mutations (G1764A) decrease HBeAg expression at the transcriptional level [5]. HBeAg is an immune target, and lack of its expression may result in loss of immune responses and subsequently liver disease progression [7, 45]. Notably, the sample with G1896A mutation was HBeAg negative. The G1896A mutation causes the conversion of TGG→TAG (Trp → Stop codon) at codon 28 of the pre-C gene and the suppression of HBeAg expression. Whereas, the BCP mutations enhance the replication rate of the HBV genome [5]. The BCP and pre-C mutations are associated with the progression of chronic HBV infection to advanced liver disease and are frequently found in patients with chronic hepatitis, fibrosis, liver cirrhosis, and HCC [2, 7]. Therefore, early detection of these mutations is pivotal in predicting the clinical progression in patients with chronic HBV infection and deciding the antiviral medications. No nucleotide insertions, deletions, or frameshifting were observed in our samples.

The dominance of genotype D is the most important characteristic of HBV infection among our pregnant women, which reflects the genotypic pattern of HBV infection in our region. Genotype D is also the predominant genotype in Iran [46]. To date, 10 HBV genotypes (A to J) with 9 serological subtypes and over 35 sub-genotypes related to genotypes A–D, F, H, and I have been identified to be not only predictive of response to antiviral treatment and disease progression but also associated with modes of transmission and geographical regions [6, 10]. Of them, genotype

Formatted: Highlight

Formatted: Highlight

Formatted: Highlight

Formatted: Highlight

D is the main genotype in Africa, Europe, India, Indonesia and the Mediterranean countries, and is characterized by chronicity, worse clinical outcomes (cirrhosis and HCC), low response to IFN-based therapy, and a high frequency of BCP and pre-C mutations [10, 11]. The pre-C G1896A mutation is frequently found in HBV genotypes B, D, and E [45]. Moreover, the BCP A1762T and G1764A mutations are reported in association with HBV genotypes C and D [47]. On the other hand, sub-genotype D3 is prevalent among injecting drug users [48]. Like HCV infection [49], injecting drug use is most probably the main route of HBV transmission in this region. However, we were unable to confirm this claim, since the majority of women were not willing to answer the questions regarding the intravenous drug use by themselves or more importantly by their spouses.

This is the first report on the prevalence of HBV infection among pregnant women resident in the northern shores of the Persian Gulf, and the first to investigate the seroprevalence of HBsAg and HBcAb among pregnant Afghan migrant women in Iran. Moreover, the results of this study can be generalized to the pregnant population of this territory due to the consecutive recruitment of participants and the high number of the assessed pregnant women. However, due to the cross-sectional design of the study, we were not able to determine the eventual effects of hepatitis B on pregnancy outcomes and the infants born to infected mothers. HBV mutations on the progression of HBV infection among pregnant women. As another limitation, the possible effects of hepatitis B on pregnancy outcomes remained unclear among pregnant women in the South of Iran. Therefore, this report should be completed by prospective studies. Besides, due to the lack of awareness of the level of immunity against hepatitis B among the study population, this study was not able to determine the efficacy of hepatitis B vaccination among pregnant women in this region. Nevertheless, antibody to hepatitis B surface antigen (anti-HBsAg) was not assessed

Formatted: Highlight

among the study population. Therefore, there is a need to study the effectiveness of the immunization policy in the pregnant population of this region. In this study, the nested-PCR assay was used to detect HBV infection among pregnant women. This two-step PCR method has a very high sensitivity for the diagnosis of cases with low virus levels. However, in comparison with quantitative PCR (qPCR), the nested-PCR assay is not able to determine the viral loads. Moreover, the main limitation of nested-PCR is the probability of contamination that can cause false-positive results. In this study, strict quality control was applied, and positive serum samples were re-tested to ensure the absence of contamination and the accuracy of the results.

Conclusion

This study reports the HBV prevalence of 1.05% for HBsAg, 2.88% for HBcAb and 0.35% for HBV viremia with genotype D among pregnant women residents in the northern shores of the Persian Gulf, Iran. The results of this study indicate a relatively low prevalence of HBV infection among pregnant women in the South of Iran, while tattooing is a risk factor for exposure to HBV infection. Moreover, all of the HBV-positive pregnant women were asymptomatic and unaware of their infection. Therefore, routine screening for HBV markers during pregnancyConsidering perinatal transmission and the high rate of chronic HBV infection in infants, screening of women of childbearing age, appropriate treatment of HBV-infected women, and HBV vaccination are recommended to decrease mother-to-child transmission of HBV and, consequently, reduce the disease burden in the community. Moreover, we observed occult HBV infection in one sample with an undetectable level of HBsAg. Therefore, screening of pregnant women based on HBsAg detection is not reliable for the identification of occult HBV infection. The use of more sensitive molecular techniques such as nested-PCR assay is recommended to detect HBV DNA and

Formatted: Highlight

Formatted: Default Paragraph Font, Font: (Default) Times New Roman, 12 pt, Highlight

Formatted: Highlight

Formatted: Highlight

Formatted: Font: Times New Roman, 18 pt

Formatted: Heading 1, Left, Line spacing: single

Formatted: Highlight

Formatted: Highlight

increase the chance of diagnosing occult hepatitis B with low virus titers. Besides, several point replacement mutations were detected in the S, X, pre-C, and BCP regions of HBV isolated from pregnant women. Mutation analysis of HBV can be useful in predicting the disease progression in HBV-infected patients.

Acknowledgment

The authors would like to ~~acknowledge grant number 357 supported by~~thank the Deputy Research and Affairs of the Bushehr University of Medical Sciences, Bushehr, Iran.

~~**Ethical approval**~~

~~This study was approved by the Ethical Committee of Bushehr University of Medical Sciences with reference number IR.BPUMS.REC.1396.111.~~

References

1. Ghabeshi S, Baktashi R, Hosseini SM, Sharifi Z. Molecular evaluation of HBV core gene mutations in asymptomatic HBV infected blood donors in Iran. 2014.
2. Chachá SGF, Gomes-Gouvêa MS, Malta FdM, Ferreira SdC, Villanova MG, Souza FF, et al. Basal core promoter and precore mutations among hepatitis B virus circulating in Brazil and its association with severe forms of hepatic diseases. Memórias do Instituto Oswaldo Cruz. 2017;112(9):626-31.
3. Joshi SS, Coffin CS. Hepatitis B and Pregnancy: Virologic and Immunologic Characteristics. Hepatol Commun. 2020;4(2):157-71. Epub 2020/02/07. doi: 10.1002/hep4.1460. PubMed PMID: 32025602; PubMed Central PMCID: PMCPMC6996345.
4. Rajput MK. Mutations and methods of analysis of mutations in Hepatitis B virus. AIMS microbiology. 2020;6(4):401.
5. Tacke F, Gehrke C, Luedde T, Heim A, Manns MP, Trautwein C. Basal core promoter and precore mutations in the hepatitis B virus genome enhance replication efficacy of Lamivudine-resistant mutants. J Virol. 2004;78(16):8524-35. Epub 2004/07/29. doi: 10.1128/JVI.78.16.8524-8535.2004. PubMed PMID: 15280461; PubMed Central PMCID: PMCPMC479060.
6. Kramvis A. Genotypes and genetic variability of hepatitis B virus. Intervirology. 2014;57(3-4):141-50. Epub 2014/07/19. doi: 10.1159/000360947. PubMed PMID: 25034481.

Formatted: Font: Times New Roman, 18 pt

Formatted: Heading 1, Left, Line spacing: single

Formatted: Font: Times New Roman, 18 pt

Formatted: Heading 1, Left, Line spacing: single

7. Yan L, Zhang H, Ma H, Liu D, Li W, Kang Y, et al. Deep sequencing of hepatitis B virus basal core promoter and precore mutants in HBeAg-positive chronic hepatitis B patients. Sci Rep. 2015;5:17950. Epub 2015/12/10. doi: 10.1038/srep17950. PubMed PMID: 26647737; PubMed Central PMCID: PMCPMC4673698.

8. Ledesma MM, Galdame O, Bouzas B, Tadey L, Livellara B, Giuliano S, et al. Characterization of the basal core promoter and precore regions in anti-HBe-positive inactive carriers of hepatitis B virus. Int J Infect Dis. 2011;15(5):e314-20. Epub 2011/03/04. doi: 10.1016/j.ijid.2010.12.009. PubMed PMID: 21367634.

9. El Chaar M, Candotti D, Crowther RA, Allain JP. Impact of hepatitis B virus surface protein mutations on the diagnosis of occult hepatitis B virus infection. Hepatology. 2010;52(5):1600-10. Epub 2010/09/04. doi: 10.1002/hep.23886. PubMed PMID: 20815025.

10. Lin CL, Kao JH. Hepatitis B virus genotypes and variants. Cold Spring Harb Perspect Med. 2015;5(5):a021436. Epub 2015/05/03. doi: 10.1101/cshperspect.a021436. PubMed PMID: 25934462; PubMed Central PMCID: PMCPMC4448583.

11. Sunbul M. Hepatitis B virus genotypes: global distribution and clinical importance. World J Gastroenterol. 2014;20(18):5427-34. Epub 2014/05/17. doi: 10.3748/wjg.v20.i18.5427. PubMed PMID: 24833873; PubMed Central PMCID: PMCPMC4017058.

12. Chilaka VN, Konje JC. Viral Hepatitis in pregnancy. Eur J Obstet Gynecol Reprod Biol. 2021;256:287-96. Epub 2020/12/02. doi: 10.1016/j.ejogrb.2020.11.052. PubMed PMID: 33259998.

13. Piratvisuth T. Optimal management of HBV infection during pregnancy. Liver International. 2013;33:188-94.

14. Navabakhsh B, Mehrabi N, Estakhri A, Mohamadnejad M, Poustchi H. Hepatitis B virus infection during pregnancy: transmission and prevention. Middle East journal of digestive diseases. 2011;3(2):92.

15. Badfar G, Shohani M, Nasirkandy MP, Mansouri A, Abangah G, Rahmati S, et al. Epidemiology of hepatitis B in pregnant Iranian women: a systematic review and meta-analysis. Arch Virol. 2018;163(2):319-30. Epub 2017/10/25. doi: 10.1007/s00705-017-3551-6. PubMed PMID: 29063378.

16. Brown RS, Jr., McMahon BJ, Lok AS, Wong JB, Ahmed AT, Mouchli MA, et al. Antiviral therapy in chronic hepatitis B viral infection during pregnancy: A systematic review and meta-analysis. Hepatology. 2016;63(1):319-33. Epub 2015/11/14. doi: 10.1002/hep.28302. PubMed PMID: 26565396.

17. Mohammadi Z, Keshtkar A, Eghtesad S, Jeddian A, Pourfatholah AA, Maghsudlu M, et al. Epidemiological profile of hepatitis B virus infection in Iran in the past 25 years; a systematic review and meta-analysis of general population studies. Middle East journal of digestive diseases. 2016;8(1):5.

18. Farshadpour F, Taherkhani R, Tajbakhsh S, Gholizadeh Tangestani M, Hajiani G, Sharifi N, et al. Prevalence and trends of transfusion-transmissible viral infections among blood donors in south of Iran: an eleven-year retrospective study. PloS one. 2016;11(6):e0157615.

19. Moghaddasifar I, B Lankarani K, Moosazadeh M, Afshari M, Malary M. Prevalence of hepatitis B virus infection among pregnant women in Iran: a systematic review and Meta-analysis. Iranian Journal of Cancer Prevention. 2016;9(6).

20. Sitnik R, Pinho JR, Bertolini DA, Bernardini AP, Da Silva LC, Carrilho FJ. Hepatitis B virus genotypes and precore and core mutants in Brazilian patients. J Clin Microbiol.

2004;42(6):2455-60. Epub 2004/06/09. doi: 10.1128/JCM.42.6.2455-2460.2004. PubMed PMID: 15184419; PubMed Central PMCID: PMCPMC427827.

21. Chen J, Yin J, Tan X, Zhang H, Zhang H, Chen B, et al. Improved multiplex-PCR to identify hepatitis B virus genotypes A-F and subgenotypes B1, B2, C1 and C2. J Clin Virol. 2007;38(3):238-43. Epub 2007/01/17. doi: 10.1016/j.jcv.2006.12.007. PubMed PMID: 17224304.

22. Takahashi K, Aoyama K, Ohno N, Iwata K, Akahane Y, Baba K, et al. The precore/core promoter mutant (T1762A1764) of hepatitis B virus: clinical significance and an easy method for detection. J Gen Virol. 1995;76 (Pt 12):3159-64. Epub 1995/12/01. doi: 10.1099/0022-1317-76-12-3159. PubMed PMID: 8847524.

23. Boot HJ, Cremer J, Koedijk FD, van Ballegooijen WM, Op de Coul EL. Improved tracing of hepatitis B virus transmission chains by phylogenetic analysis based on C region sequences. J Med Virol. 2008;80(2):233-41. Epub 2007/12/22. doi: 10.1002/jmv.21080. PubMed PMID: 18098131.

24. Italiano CM, Speers DJ, Chidlow GR, Dowse GK, Robertson AG, Flexman JP. Hepatitis B outbreak following a mass-casualty incident, Australia. J Infect Dis. 2011;204(3):400-7. Epub 2011/07/12. doi: 10.1093/infdis/jir269. PubMed PMID: 21742838.

25. Taherkhani R, Farshadpour F, Makvandi M, Hamidifard M, Esmailizadeh M, Ahmadi B, et al. Determination of cytomegalovirus prevalence and glycoprotein B genotypes among ulcerative colitis patients in Ahvaz, Iran. Jundishapur Journal of Microbiology. 2015;8(2).

26. Bayani M, Biazar T, HASANJANI RM, Bayani F, Siadati S. The effect of hepatitis B vaccination at birth on reducing the prevalence of hepatitis B surface antigen among rural pregnant women in Babol, Iran. 2016.

27. Pourafzali SJ, Khaledi A, Miri R, Shamsian SAA, Rezaee SA, Piroozmand A, et al. Prevalence of Hepatitis B Surface Antigen and Associated Risk Factors with Hepatitis B Infection among Pregnant Women in Northeast of Iran. International Journal of Medical Laboratory. 2018;5(3):222-8.

28. Kheiri L, Makvandi S. The prevalence of Hepatitis B surface antigen (HBsAg) and its influencing factors in pregnant women referring to Healthcare Centers of Dehloran, Iran in 2011-2012. Journal of Midwifery and Reproductive Health. 2015;3(3):424-9.

29. Mohebbi SR, Sanati A, Cheraghipour K, Nejad MR, Shalmani HM, Zali MR. Hepatitis C and hepatitis B virus infection: epidemiology and risk factors in a large cohort of pregnant women in Lorestan, West of Iran. Hepatitis monthly. 2011;11(9):736.

30. Shoghli A, Nabavi SM, Alavian SM, Kolifarhood G, Goya MM, Namazi R, et al. Hepatitis B surface antigen prevalence in pregnant women: A cross-sectional survey in Iran. International journal of preventive medicine. 2014;5(Suppl 3):S213.

31. Aminzadeh Z, Shabani Shahrbabaki Z, Ghachkar L, Sayyadi Anari AR. Hepatitis B Surface Antigen Prevalence among Pregnant Women in Rafsanjan City, Iran, 2003. Iranian Journal of Virology. 2008;2(3):19-20.

32. Sahaf F, Tanomand A, Montazam H, Sany A. Seroprevalence of hepatitis C, hepatitis B and HIV and co-infection among pregnant women: A retrospective study in 2006 at Malekan city, Iran. Res J Med Sci. 2007;1(2):138-41.

33. Souza MT, Pinho TLRd, Santos MDC, Santos Ad, Monteiro VL, Fonsêca LMB, et al. Prevalence of hepatitis B among pregnant women assisted at the public maternity hospitals of São Luís, Maranhão, Brazil. Brazilian Journal of Infectious Diseases. 2012;16(6):517-20.

34. Rajendiran S, Gopalan U, Jayakumar K. Seroprevalence of hepatitis B infection among pregnant women in South India. International Journal of Reproduction, Contraception, Obstetrics and Gynecology. 2017;6(1):249-52.
35. Kwadzokpui PK, Akorsu EE, Abaka-Yawson A, Quarshie SS, Amankwah SA, Tawiah PA. Prevalence and knowledge of hepatitis B virus infection among pregnant women in the Ningo-Prampram district, Ghana. International journal of hepatology. 2020;2020.
36. Oti V, Mohammed I, Ibrahim Y, Ibrahim C, Orok I. Epidemiologic Survey of HBV, HCV and HIV Infections in a Pregnant Women Population in Central Nigeria: A Cross-Sectional Study. J Infect Dis Epidemiol. 2021;7:194.
37. Ding Y, Sheng Q, Ma L, Dou X. Chronic HBV infection among pregnant women and their infants in Shenyang, China. Virology journal. 2013;10(1):1-5.
38. Tanga AT, Teshome MA, Hiko D, Fikru C, Jilo GK. Sero-prevalence of hepatitis B virus and associated factors among pregnant women in Gambella hospital, South Western Ethiopia: facility based cross-sectional study. BMC infectious diseases. 2019;19(1):1-7.
39. Bittaye M, Idoko P, Ekele BA, Obed SA, Nyan O. Hepatitis B virus sero-prevalence amongst pregnant women in the Gambia. BMC Infect Dis. 2019;19(1):259. Epub 2019/03/17. doi: 10.1186/s12879-019-3883-9. PubMed PMID: 30876397; PubMed Central PMCID: PMCPMC6419830.
40. Noubiap JJN, Nansseu JRN, Ndoula ST, Bigna JJR, Jingi AM, Fokom-Domgue J. Prevalence, infectivity and correlates of hepatitis B virus infection among pregnant women in a rural district of the Far North Region of Cameroon. BMC public health. 2015;15(1):1-7.
41. Murad EA, Babiker SM, Gasim GI, Rayis DA, Adam I. Epidemiology of hepatitis B and hepatitis C virus infections in pregnant women in Sana’a, Yemen. BMC pregnancy and childbirth. 2013;13(1):1-5.
42. Bancha B, Kinfe AA, Chanko KP, Workie SB, Tadese T. Prevalence of hepatitis B viruses and associated factors among pregnant women attending antenatal clinics in public hospitals of Wolaita Zone, South Ethiopia. PloS one. 2020;15(5):e0232653.
43. Roznovsky L, Harrison TJ, Fang ZL, Ling R, Lochman I, Orsagova I, et al. Unusual hepatitis B surface antigen variation in a child immunised against hepatitis B. J Med Virol. 2000;61(1):11-4. Epub 2000/04/04. doi: 10.1002/(sici)1096-9071(200005)61:1<11::aid-jmv2>3.0.co;2-9. PubMed PMID: 10745226.
44. Avellon A, Echevarria JM. Frequency of hepatitis B virus ‘a’determinant variants in unselected Spanish chronic carriers. Journal of medical virology. 2006;78(1):24-36.
45. Juniastuti, Utsumi T, Aksono EB, Yano Y, Soetjipto, Hayashi Y, et al. Predominance of precore mutations and clinical significance of basal core promoter mutations in chronic hepatitis B virus infection in Indonesia. Biomed Rep. 2013;1(4):522-8. Epub 2014/03/22. doi: 10.3892/br.2013.106. PubMed PMID: 24648979; PubMed Central PMCID: PMCPMC3917060.
46. Haghshenas MR, Arabi M, Mousavi T. Hepatitis B genotypes in Iran. Mater Sociomed. 2014;26(2):129-33. Epub 2014/06/20. doi: 10.5455/msm.2014.26.129-133. PubMed PMID: 24944540; PubMed Central PMCID: PMCPMC4035145.
47. Lin CL, Kao JH. Natural history of acute and chronic hepatitis B: The role of HBV genotypes and mutants. Best Pract Res Clin Gastroenterol. 2017;31(3):249-55. Epub 2017/08/05. doi: 10.1016/j.bpg.2017.04.010. PubMed PMID: 28774406.
48. Ozaras R, Inanc Balkan I, Yemisen M, Tabak F. Epidemiology of HBV subgenotypes D. Clin Res Hepatol Gastroenterol. 2015;39(1):28-37. Epub 2014/07/20. doi: 10.1016/j.clinre.2014.06.005. PubMed PMID: 25037178.

49. Taherkhani R, Farshadpour F. Lurking epidemic of hepatitis C virus infection in Iran: A call to action. World J Hepatol. 2017;9(24):1040-2. Epub 2017/09/22. doi: 10.4254/wjh.v9.i24.1040. PubMed PMID: 28932350; PubMed Central PMCID: PMCPMC5583536.

Supporting information

S1 Fig. Alignment of the amino acid sequences of HBsAg (44-170 aa) of strains isolated from pregnant women (GenBank accession Nos. OK490614-OK490616) and the reference sequences available at the GenBank.

S2 Fig. Alignment of the amino acid sequences of pre-core region isolated from pregnant women (GenBank accession Nos. OK490617 and OK490618) and the reference sequences available at the GenBank.

S3 Fig. Alignment of the amino acid sequences (109-154 aa) of X protein (1698-1838 bp nucleotide sequence) isolated from pregnant women (GenBank accession Nos. OK490617 and OK490618) and the reference sequences available at the GenBank.

S1 Table. Prevalence of HBsAg according to socio-demographic and qualitative variables among pregnant women in the South of Iran.

S2 Table. Prevalence of HBcAb according to socio-demographic and qualitative variables among pregnant women in the South of Iran.

Formatted: Font: Times New Roman, 18 pt

Formatted: Heading 1

Formatted: Font: 18 pt

Formatted: Highlight

Formatted: Highlight

Formatted: Highlight

Formatted: Font: Bold

Formatted: Font: Bold

Formatted: Font: Bold

Formatted: Font: Bold

Formatted: Font: Bold

ANSWERING REVIEWERS

December 15, 2021

Dear Editor-in-Chief of
PLOS ONE

We would greatly appreciate for your consideration of the publication of our manuscript entitled "Prevalence, genotype distribution and mutations of hepatitis B virus infection and associated risk factors among pregnant women resident in the northern shores of Persian Gulf, Iran" in the *PLOS ONE*.

Ms. No. PONE-D-21-32739

Title: Prevalence, genotype distribution and mutations of hepatitis B virus infection and associated risk factors among pregnant women resident in the northern shores of Persian Gulf, Iran

Name of Journal: *PLOS ONE*

Revision has been made according to the suggestions of the reviewers and the editor.

We greatly appreciated the reviewers' comments. The point-to-point responses to comments were shown as follows:

Journal Requirements:

When submitting your revision, we need you to address these additional requirements.
1. Please ensure that your manuscript meets PLOS ONE's style requirements, including those for file naming. The PLOS ONE style templates can be found at
https://journals.plos.org/plosone/s/file?id=wjVg/PLOSOne_formatting_sample_main_body.pdf
and
https://journals.plos.org/plosone/s/file?id=ba62/PLOSOne_formatting_sample_title_authors_affiliations.pdf
Response: The PLOS ONE's style requirements were applied.

2. Thank you for stating the following in the Acknowledgments Section of your manuscript:
"The authors would like to acknowledge grant number supported by the Deputy Research and 393 Affairs of the Bushehr University of Medical Sciences, Bushehr, Iran."
We note that you have provided additional information within the Acknowledgements Section that is not currently declared in your Funding Statement. Please note that funding information

should not appear in the Acknowledgments section or other areas of your manuscript. We will only publish funding information present in the Funding Statement section of the online submission form.

Please remove any funding-related text from the manuscript and let us know how you would like to update your Funding Statement. Currently, your Funding Statement reads as follows:

"This study was funded by Bushehr University of Medical Sciences with grant number 357.

The funders had no role in study design, data collection and analysis, decision to publish, or preparation of the manuscript."

Please include your amended statements within your cover letter; we will change the online submission form on your behalf.

Response: The funding-related texts were removed from the Methods and the Acknowledgment sections of the manuscript. The funding information present in the Funding Statement section of the online submission form is correct.

3. PLOS ONE now requires that authors provide the original uncropped and unadjusted images underlying all blot or gel results reported in a submission's figures or Supporting Information files. This policy and the journal's other requirements for blot/gel reporting and figure preparation are described in detail at https://journals.plos.org/plosone/s/figures#loc-blot-and-gel-reporting-requirements and https://journals.plos.org/plosone/s/figures#loc-preparing-figures-from-image-files. When you submit your revised manuscript, please ensure that your figures adhere fully to these guidelines and provide the original underlying images for all blot or gel data reported in your submission. See the following link for instructions on providing the original image data: https://journals.plos.org/plosone/s/figures#loc-original-images-for-blots-and-gels.

In your cover letter, please note whether your blot/gel image data are in Supporting Information or posted at a public data repository, provide the repository URL if relevant, and provide specific details as to which raw blot/gel images, if any, are not available. Email us at plosone@plos.org if you have any questions.

Response: The original uncropped and unadjusted images underlying gel results were provided. In the original uncropped figure 1, seven positive samples are shown. Since, two positive samples (positive samples number 4 and 5) were loaded again in the wells number 12 and 13. The reason was we did not want to have empty wells, we tried to load all of the wells.

4. Your ethics statement should only appear in the Methods section of your manuscript. If your ethics statement is written in any section besides the Methods, please delete it from any other section.

Response: The ethics statement was removed from end of the manuscript.

5. Please include captions for your Supporting Information files at the end of your manuscript, and update any in-text citations to match accordingly. Please see our Supporting Information guidelines for more information: http://journals.plos.org/plosone/s/supporting-information.

Response: Captions for the Supporting Information files were included at the end of the manuscript.

Additional Editor Comments:

The manuscript in the present form demands a **"major revision"** before it can be published. Therefore, we invite you to submit a revised version of the manuscript that addresses the points raised during the review process.

Response: We greatly appreciate you taking the time to consider our manuscript while providing us constructive comments. Moreover, thank you very much for giving us the opportunity to answer the reviewers' comments. We are really grateful for this opportunity.

1. Please ensure that your manuscript meets PLOS ONE's style requirements, including those for headings. The PLOS ONE style templates can be found at https://journals.plos.org/plosone/s/file?id=wjVg/PLOSOne_formatting_sample_main_body.pdf

Response: The PLOS ONE's style requirements were applied.

2. Quality of written English is not suitable for publication unless extensively edited eg. The word "uneducated" should be replaced by "Illiterate".

Response: The manuscript was edited for proper English language, grammar, punctuation, and spelling by our team at Bushehr University of Medical Sciences.

3. The limitations of the study are not sufficiently discussed.

Response: The limitations of the study were discussed in more details and highlighted.

4. Abstract needs to be re-written to reflect the study more correctly eg. Amplification of X gene is not included.

Response: The abstract was re-written.

Minor comments:

Change laboratory diagnosis sub-heading to laboratory methods.

Response: The change was done.

The author should consider using subheadings in the "laboratory diagnosis" section for easier reading.

Suggestion: HBV serological testing, PCR amplification and sequencing, phylogenetic and mutational analysis or sequence analysis.

Response: The suggestions were applied.

Include the full name of the ELISA kit used.

Response: The full name of the ELISA kits was included and highlighted with green color.

Table 1- Include degrees (℃) in the annealing temperature column.

Response: The degrees (℃) in the annealing temperature column was included.

The author indicate that the sequences has been submitted to GenBank, but no accession numbers provided.

Response: The accession numbers were provided and highlighted with yellow color in the result section and the legends of Fig3 and S1-S3 Figs.

The sequences have been submitted to GenBank and the accession numbers were provided via email, but they have not been released yet.

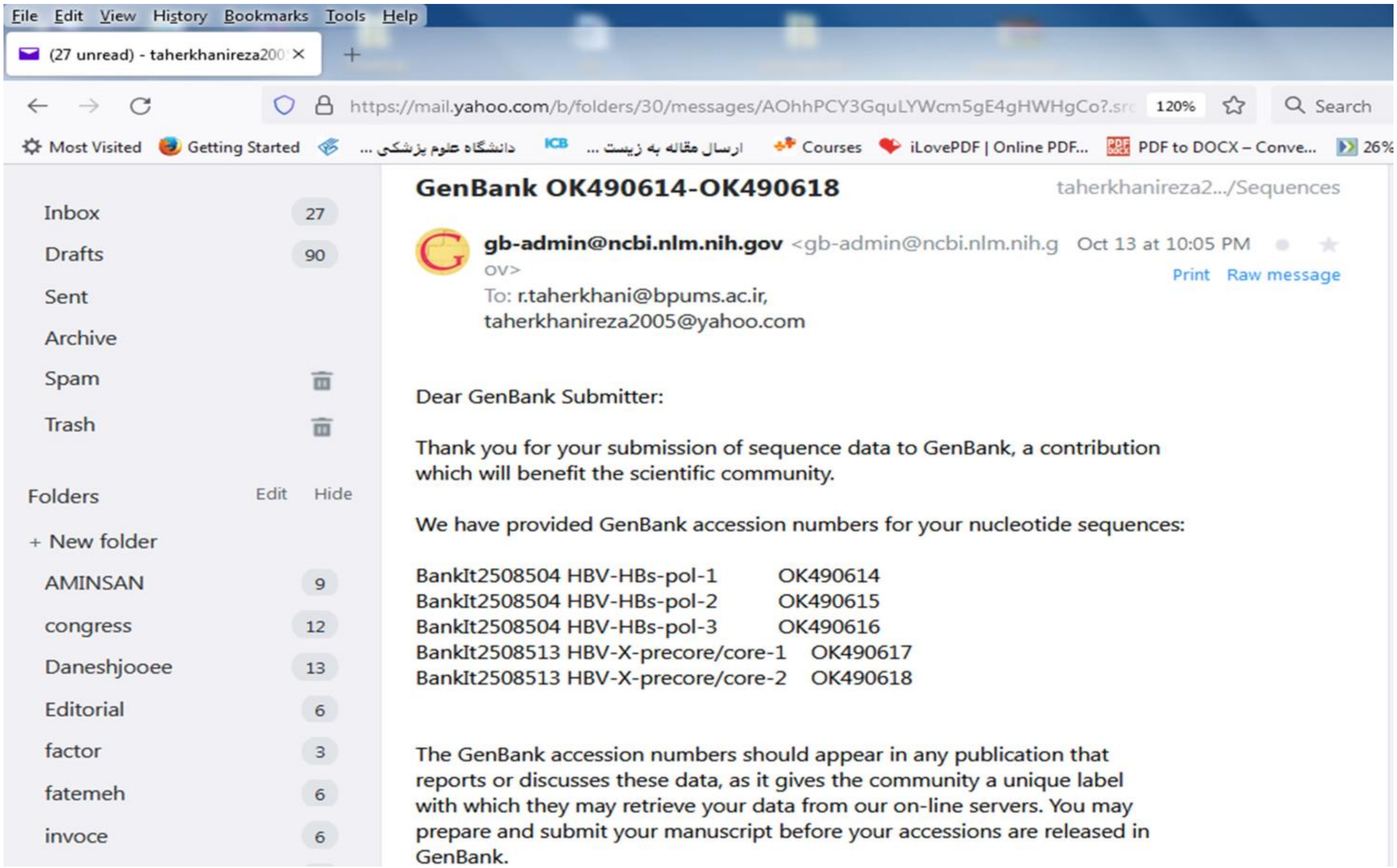

The study relied on nucleotide sequence analyses. However, there is no mention of sequencing protocol/approach/platform used in generating the nucleotide sequences in the methods section, only referenced a company that looks like it provides service.

Response: The Sanger dideoxy sequencing technology was included in the method section.

Table 2 and 3 should be moved to supplementary.

Response: The table 2 and 3 were moved to supplementary.

One of the city is stated as Khormoj in Table 2 and throughout it is stated as Khormuj.

Response: The spelling was corrected in the table 2.

Phylogenetic and mutational analysis (Figure 3 and Table 4) focused on 3 samples yet they detected HBV DNA in 5 samples. It is not clear whether the other 2 samples could not be sequenced or why they were left out of the analysis.
Response: Unfortunately, the other 2 samples had low-quality sequencing results, so that it was impossible to include them in the phylogenetic and mutational analysis.

Figure 3: Only indicate bootstrap values >70% which is considered moderate confidence.
Response: The phylogenetic tree was constructed by the neighbor-joining method with the Bootstrap test of phylogeny (substitutional model: maximum composite likelihood) in Molecular Evolutionary Genetics Analysis (MEGA) program version 7. Bootstrap resampling strategy and reconstruction were carried out 1,000 times to confirm the reliability of the phylogenetic tree.

Reviewer's Responses to Questions
Comments to the Author

1. Is the manuscript technically sound, and do the data support the conclusions?

The manuscript must describe a technically sound piece of scientific research with data that supports the conclusions. Experiments must have been conducted rigorously, with appropriate controls, replication, and sample sizes. The conclusions must be drawn appropriately based on the data presented.
Reviewer #1: No
Reviewer #2: Yes
Reviewer #3: Yes
Reviewer #4: Yes

---

2. Has the statistical analysis been performed appropriately and rigorously?
Reviewer #1: No
Reviewer #2: Yes
Reviewer #3: Yes
Reviewer #4: Yes

---

3. Have the authors made all data underlying the findings in their manuscript fully available?

The PLOS Data policy requires authors to make all data underlying the findings described in their manuscript fully available without restriction, with rare exception (please refer to the Data Availability Statement in the manuscript PDF file). The data should be provided as part of the

manuscript or its supporting information, or deposited to a public repository. For example, in addition to summary statistics, the data points behind means, medians and variance measures should be available. If there are restrictions on publicly sharing data—e.g. participant privacy or use of data from a third party—those must be specified.
Reviewer #1: Yes
Reviewer #2: Yes
Reviewer #3: Yes
Reviewer #4: Yes

---

4. Is the manuscript presented in an intelligible fashion and written in standard English?

PLOS ONE does not copyedit accepted manuscripts, so the language in submitted articles must be clear, correct, and unambiguous. Any typographical or grammatical errors should be corrected at revision, so please note any specific errors here.
Reviewer #1: No
Reviewer #2: Yes
Reviewer #3: Yes
Reviewer #4: Yes

---

5. Review Comments to the Author
Please use the space provided to explain your answers to the questions above. You may also include additional comments for the author, including concerns about dual publication, research ethics, or publication ethics. (Please upload your review as an attachment if it exceeds 20,000 characters)

Reviewer #1: The author used unacceptable language (e.g. uneducated women) which also feels derogatory. The manuscript used unnecessary statistics, while its main research was HBV infection (HBsAg or HBV DNA) or exposure as determined by HBcAb. Only 15 pregnant women were HBsAg positive but the manuscript made a lot of unnecessary and unacceptable statistical analysis (smoking, Education to mention few).
Response: Thank you very much for your review.

-The manuscript was edited for proper English language, grammar, punctuation, and spelling.

-These variables were included in the study in order to find the possible risk factors of HBV infection among pregnant women.
Regarding smoking: In addition to cigarette, some women in the South of Iran use hookah for smoking and they share the hookah in group. We thought that there might be a chance of HBV transmission through saliva by using hookah.

Regarding education: The education level has an indirect effect on the prevalence of HBV. The educated women have more information about the transmission routes of HBV.

Regarding the month of sampling: We assumed that there might be a seasonal or monthly pattern regarding the seroprevalence of HBV infection among pregnant women in this region.
According to those studies on the seasonality of hepatitis, we wanted to investigate the seasonal pattern in this study too.

Reviewer #2: This was a well-designed study that showed the prevalence and molecular characterization of HBV in pregnant woman. The literature review clearly stated the problem and the aim of the study. The methodology used was standard for this type of study.
Response: Thank you very much for your review and the positive comments.

In the results section, there is a comment on the occult status in Line 193, it is not shown what the viral load of this sample is in order to make such a statement. I suggest the author includes viral load of this sample.
Response: Occult HBV infection is described by the presence of HBV DNA in the absence of detectable HBsAg in serum or plasma. This sample, despite being positive for HBV DNA, was negative for HBsAg, which is indicative of an occult HBV infection.

-We did not measure HBV viral loads since the nested PCR assay used in this study is a qualitative method and is not able to determine the viral loads. This is a limitation of nested-PCR over quantitative PCR (qPCR). The limitations of nested PCR assay were included in the manuscript and highlighted with yellow color in the limitations section of the study.

-Those samples with very low virus levels become positive in the second round of nested PCR. This sample with occult HBV infection was identified in the second round of amplification, which is indicative of low HBV DNA levels.

- In one of our previous research projects (unpublished data), the viral load of one of the HBV DNA positive serum samples was measured by real time PCR, and the serum sample was serial diluted. The diluted sample with 25 HBV DNA copies/ml (5 HBV DNA copies/200 μL) became positive in the second round of nested PCR, while it was negative in the first round of PCR.

In the discussion, in Line 255, the author states that the HBV prevalence of 1.05% was lower than 1.18%, these are similar especially if rounded off.
Response: We cannot round off the numbers here. Because regarding the prevalence rate, even a small decimal is important. We used “slightly” in the sentence.

In line 258, Risk of mother to child transmission should be discussed more here, they did not show what the risk of undiagnosed HBV in pregnancy would be, especially in those infected in the third trimester.
Response: The mentioned issue was included and highlighted with yellow color.

Line 268, This recommendation is not clear. Do they mean that they will test each and every women in the South of Iran area? this is not cost effective, rather screen all women who come in for their first visit at the ANC and vaccinate the baby at birth. Or treat the infection if it becomes chronic.
Response: We agree with the dear reviewer. This sentence was revised and highlighted with green color.

Line 319, Cannot make this conclusion as this was not part of the questions asked in the questionnaire as shown in the information on Table 2 and 3. You only have vaccination data for 14 participants out of over 1000. This is being very presumptuous and this conclusion needs to be made with caution.
Response: We agree with the dear reviewer. Therefore, the sentence was revised and highlighted with green color.

-A significant percentage of the pregnant women in this study were not vaccinated according to self-declaration during the interview. Only 22.0% of the participants were vaccinated, 22.0% were not vaccinated and 55.9% of the participants did not respond to the questions regarding history of HBV vaccination during interview (Unknown group). These participants did not know anything about HBV vaccination. Considering the compulsory vaccination of infants since 1993 and teenagers since 2006 in Iran, individuals above 30 years were not the target group of national vaccination program. Since a high number of the "Unknown group" were above 30 years, we think that a significant percentage of the pregnant women in this study were not vaccinated. These data are shown in the tables 2 and 3.

-According to the aim of the study and the available funding, the presence of hepatitis B surface antigen (HBsAg), hepatitis B core antibody (HBcAb) and HBV viremia was investigated in serum samples of the participants. Unfortunately, we did not assess antibody to hepatitis B surface antigen (anti-HBsAg) among the study population due to lack of budget. This issue was mentioned as the limitation of the study, and it is suggested to study the effectiveness of the immunization policy in the pregnant population of this region.

Line 326, this statement contradicts what the authors had said in Line 318-320 on the ineffectiveness of the HBV vaccination program.
Response: The previous statement has been revised.

-The previous statement was about the seroprevalence of HBV in the age group 20-29 years old. This statement is about the pregnant women under 20 years old.

Line 331, What was the viral load of this sample? it was also not mentioned in the results section.

Response: We did not measure HBV viral loads since the nested PCR assay used in this study is a qualitative method and is not able to determine the viral loads. This a limitation of nested-PCR over qPCR. The limitations of nested PCR assay were included in the manuscript.

Those samples with very low virus levels become positive in the second round of nested PCR. This sample with occult HBV infection was identified in the second round of amplification, which is indicative of low HBV DNA levels.

-In one of our previous research projects (unpublished data), the viral load of one of the HBV DNA positive serum samples was measured by real time PCR, and the serum sample was serial diluted.

The diluted serum sample with 25 HBV DNA copies/ml (5 HBV DNA copies/200 μL) became positive in the second round of nested PCR, while it was negative in the first round of PCR.

Line 339-340, show the importance of this lack of HBeAg during pregnancy, this has not been discussed fully here.

Response: No, this sentence does not show the importance of the lack of HBeAg during pregnancy. This shows the relationship between the expression of HBeAg and immune responses to HBV infection.

It shows that the absence of HBeAg may result in evasion of immune clearance as HBeAg is a target antigen for immune recognition.

The sample with G1896A mutation was HBeAg negative. The G1896A mutation causes the conversion of TGG→TAG (Trp → Stop codon) at codon 28 of the pre-C gene and the suppression of HBeAg expression. This HBeAg negative sample is at the risk of liver disease progression.

In fact, it wants to show that the mutation analysis of HBV infection can be useful in predicting the disease progression in HBV-infected patients.

The Conclusion on Line 376 differs from the conclusion on the abstract. The one in the abstract lines up with what the authors set out to do, while the one in Line 376 is a repetition of what the results stated.

Response: The conclusion was revised and highlighted.

Reviewer #3: Title: suggestion, change “resident” to “residing”

Response: Thank you very much for your review and the constructive comments.

- The suggestion was applied and highlighted with yellow color.

Abstract: Page 2, line 32 please mention the name of the ELISA kit used, company and the sentence that follows should read tested for the presence of HBV DNA delete “detection”.
Response: The full name of the ELISA kits was included.

Page 2, line 34 the software or platforms used to analyze the mutations are not mentioned.
Response: The software was included.

Page 2, line 43, the sentence talking about tattooing being a risk factor should come in the conclusion. Or the sentence can either be stated as significantly associated or as risk factor not two of them.
Response: The abstract was revised.

Page 2, line 36, did the HBcAb positive women have HBsAg or were they only positive for HBcAb? This is because in your results you state that 2.7% of HBcAb positive were also positive for HBV DNA.
Response: Of 41 HBcAb seropositive women, 4 women were positive for HBsAg (HBsAg positive and HBcAb positive), and 37 women were negative for HBsAg (HBsAg negative and HBcAb positive).

Overall, 26.7% of HBsAg seropositive pregnant women and 2.7% of HBcAb seropositive women had HBV viremia with genotype D, sub-genotype D3, and subtype ayw2:

2.7% (1 case of 37 HBsAg negative and HBcAb positive cases)
26.7% (4 cases of 15 HBsAg positive cases)

Page ,2 line 46 Please indicate how many had which genotype.
Response: All of the HBV DNA positive samples had HBV viremia with genotype D, sub-genotype D3 and subtype ayw2.

Page 2, line 49, in the methods the authors mention the pre-core regions however in the results they mention pre-core and basal core regions.
Response: The target genes were included.

Page 2, line 48, it is important to note that the mutations were detected in the same sample deemed as an occult, it is not clear in the statement.

Response: The mutations were not specific to one sample. The mutations were detected in the S, X, BCP and pre-C regions of the HBV genome isolated from pregnant women and were not specific to the occult HBV sample.
According to mutations analyses, seven amino acid substitutions in the HBsAg, one point mutation in the pre-C region and five points mutations in the basal core promoter (BCP) region were detected. Besides, the BCP mutations caused amino acid substitutions in the X protein.

Page 2, line 50 to 53, the authors are not concluding on their findings but are making a general remark. First it should be clear if HBV is endemic or not in this region and if perinatal transmission is a problem or not. In their results the authors found a very low prevalence, they should say something about it in their conclusion.
Response: The conclusion was revised.

Page 4, line 95, please add the information about the target genes missing in the abstract.
Response: The target genes were included.

Page 7, line 167, means 1.6% of the vaccinated women had chronic or acute infection.
Response: Yes, it means 1.6% of the vaccinated women were HBsAg seropositive, or the seroprevalence of HBsAg among vaccinated women was 1.6%.

Page 8, line 177, the authors did not mention, any liver enzyme tests performed however they are mentioning them in their results.
Response: Levels of the liver enzymes were obtained from the records of pregnant women at the public health centers. The pregnant women attend these public health centers for periodical checkups.

Page 8, line 177-179, the tables should also be referred to in the text.
Response: The tables were referred to in the text.

Page 11, line 190, a suggestion to delete “evaluation” to “testing”
Response: The suggestion was applied.

Page 11, line 190 the authors should stick to one decimal after the comma throughout the document for consistency.
Response: We cannot round off the numbers here. Because regarding the prevalence rate, even a small decimal is important.

Page 14, line 258 to 263 the sentence is too long.
Response: The long sentence was rephrased.

Why did the authors not check the women vaccination status? Why did the authors not test for Anti-HBs but the authors are making a statement in the discussion that majority of women are not vaccinated in this region? Do the authors mean to tell us that there is no HBV routine testing in pregnant women in this region?

Response: A significant percentage of the pregnant women in this study were not vaccinated according to self-declaration during the interview. Only 22.0% of the participants were vaccinated, 22.0% were not vaccinated and 55.9% of the participants did not respond to the questions regarding history of HBV vaccination during interview (Unknown group). These participants did not know anything about HBV vaccination. Considering the compulsory vaccination of infants since 1993 and teenagers since 2006 in Iran, individuals above 30 years were not the target group of national vaccination program. Since a high number of the "Unknown group" were above 30 years, we think that a significant percentage of the pregnant women in this study were not vaccinated. These data are shown in the tables 2 and 3.

-According to the aim of the study and the available funding, the presence of hepatitis B surface antigen (HBsAg), hepatitis B core antibody (HBcAb) and HBV viremia was investigated in serum samples of the participants. Unfortunately, we did not assess antibody to hepatitis B surface antigen (anti-HBsAg) among the study population due to lack of budget. However, we agree with the dear reviewer that the lack of awareness of the level of immunity among the study population can influence interpretation of the study findings. This issue was mentioned as the limitation of the study, and it is suggested to study the effectiveness of the immunization policy in the pregnant population of this region.

-Yes, there is no guideline or national policy on HBV screening during pregnancy. In fact, this study was performed to convince the health care providers to include HBV screening in the routine screening of pregnant women in Iran. Currently, pregnant women are only screened for rubella and CMV in Iran.

Page 16, line 319-320 the authors cannot claim that the HBV vaccination programs are not effective while they did not report if these people had received the vaccine or not and check why if they did not received the vaccine?

Response: We agree with the dear reviewer. Therefore, the sentence was revised and highlighted with green color.

- This issue was mentioned as the limitation of the study, and it is suggested to study the effectiveness of the immunization policy in the pregnant population of this region.

Page 17, line 323, how did the authors arrive at a conclusion that the HBV management during pregnancy is facing challenges?

Response: Because although there are HBV vaccine and oral antivirals and screening tools for the prevention, treatment and diagnosis of HBV infection, the status of immunity and prevalence of HBV infection among pregnant population in Iran is unknown. Since, there is no guideline or national policy on HBV screening during pregnancy. We need such kind of studies among pregnant women to convince the health care providers to include HBV screening in the management program of pregnant women in Iran.

Page 17, line 332 please rephrase the sentence.
Response: The sentence was rephrased and highlighted with yellow color.

This sample was found to be positive in the second round of PCR; this statement is irrelevant in the discussion.
Response: The sentence was removed.

Page 17, line 340, the authors state that one sample was negative for HBeAg however this was not mentioned in the methods that was tested in any sample.
Response: Unfortunately, the presence of HBeAg was not investigated in the serum samples of all participants due to the lack of budget (the available funding could not cover the expenses to test all of the samples). Only the seropositive samples were tested for the presence of HBeAg. Therefore, detection of HBeAg was not mentioned in the methods section.

Page 19, line 374, the statement is not clear.
Response: Due to the cross-sectional design of the study, we could not determine the possible effects of hepatitis B on pregnancy outcomes. Therefore, prospective or longitudinal studies are required to determine the possible effects of hepatitis B on pregnancy outcomes among pregnant women in the South of Iran.
Prospective studies are required to complete our study regarding the possible effects of hepatitis B on pregnancy outcomes.

General comments
The authors should try to keep their sentences short.
Response: The long sentences were rephrased.

The pictures are not clear; they are not of good quality.
Response: The pictures with high quality were provided.

The authors in their conclusion make no mention of the risk factors associated with HBV infection in their study.
Response: The conclusion was revised.

The authors can summarize the results section in the abstract.
Response: The abstract was revised.

Information on vaccination in the study area is not mentioned in the introduction, the vaccination coverage, when did the vaccination starts, which age group is targeted, the HBV testing program for pregnant women in this area is also missing in the introduction. These informs the reader about the situation in the study area and supports why study was conducted.
Response: The information on vaccination is mentioned in the introduction and highlighted with yellow color.
-There is no national policy on HBV screening during pregnancy in Iran. In fact, this study was performed to convince the health care providers to include HBV screening in the routine screening of pregnant women in Iran. Currently, pregnant women are only screened for rubella and CMV in Iran.

There is no made mention of how the sample size was calculated only how the study sites were identified.
Response: This is a cross-sectional study that was performed from January 2018 to June 2019. During the study period, all pregnant women who agreed to participate and gave written informed consent to use their samples for HBV detection and their data for analysis were included consecutively in this study.

-This has been mentioned in the methods section: "From January 2018 to June 2019, all the pregnant women attending these public health centers for routine visits were included consecutively in this study. The pregnant women gave written informed consent to participate in the study and use their serum samples for HBV detection."

Reviewer #4: While the findings of this study will fill a critical knowledge gap and contribute to informing interventions aimed at preventing HBV mother-to-child transmission in Iran, there is need for further clarity in the reporting of the methodology followed and results obtained in order to improve the quality of the manuscript. In addition, the limitations of the study are not explicitly addressed in the manuscript.
Response: Thank you very much for your review and the positive comments. The mentioned issues were included and highlighted.

Reviewer's Comments
Given the increased lifetime risk for potentially fatal chronic liver disease following perinatally acquired hepatitis B virus (HBV) infection, this study sought to characterize the burden of HBV infection among pregnant women living in the South of Iran. While the findings of this study will fill a critical knowledge gap and contribute to informing interventions aimed at preventing

HBV mother-to-child transmission in Iran, there is need for further clarity in the reporting of the methodology followed and results obtained in order to improve the quality of the manuscript.
Response: Thank you very much for your review and the positive comments.

Major Revisions:
The introduction section does not address universal hepatitis B vaccination globally and in Iran, and how this has influenced the burden of disease over time. In addition, the authors do not address the availability, accessibility and utilization of maternal HBV screening and management programmes in Iran. These should be addressed to provide further clarity to the context of the study.
Response: The mentioned issue was included and highlighted with yellow color in the introduction section.
-Unfortunately, there is no guideline or national policy on HBV screening during pregnancy. In fact, this study was performed to convince the health care providers to include HBV screening in the routine screening of pregnant women in Iran. Currently, pregnant women are only screened for rubella and CMV in Iran.

In the methods section of the manuscript, the authors adequately address how socio-demographic, and clinical data were collected from study participants. However, there is no information on how blood samples were collected for the study.
Response: The leftover serum samples of pregnant women who attend the public health centers for routine checkups were used for HBV detection.

The authors allude to the use of a questionnaire in collecting relevant participant data. For validation purposes, kindly indicate of this questionnaire was piloted. Was a previously published questionnaire used during this study (in which case it should be appropriately cited and referenced) or if a study specific one was developed, can a template be provided as part of supplementary material.
Response: The socio-demographic characteristics and pregnancy information were obtained by interviewing each pregnant woman using a questionnaire (a study specific one was developed). The questionnaire was translated to English and provided as supplementary.

The authors have not addressed why antibodies to the hepatitis B surface antigen (anti-HBs) was not tested among the study population. It will be interesting to understand from the introduction section how long Iran has had a universal hepatitis B vaccination programme and whether any of the younger participants should have been vaccinated in infancy. A lack of awareness of the level of immunity (either due to vaccination or recovery from a past infection) among the study population may influence interpretation of the study findings and should therefore be adequately addressed.

Response: According to the aim of the study and the available funding, the presence of hepatitis B surface antigen (HBsAg), hepatitis B core antibody (HBcAb) and HBV viremia was investigated in serum samples of the participants. Unfortunately, we did not assess antibody to hepatitis B surface antigen (anti-HBsAg) among the study population due to lack of budget. However, we agree with the dear reviewer that the lack of awareness of the level of immunity among the study population can influence interpretation of the study findings. This issue was mentioned as the limitation of the study, and it is suggested to study the effectiveness of the immunization policy in the pregnant population of this region.

The limitations of the study are not explicitly addressed in the manuscript.
Response: The limitations of the study were discussed in more details and highlighted.

Minor Revisions:
Line 61, page 3; "HBV-related liver diseases are responsible for approximately 8,00,000 deaths…" Consider revising the number format (800 000) for better clarity.
Response: The number format was revised.

Lines 116 – 117, page 5; "…using commercially available ELISA kits (DIA.PRO, Milan, Italy)." For better clarity and reliability, the authors should briefly provide further information (e.g., type of platform used, automated or manual, etc) about the ELISA used. In addition, did the authors follow the manufacturer's instructions or a published protocol (cite appropriately).
Response: The mentioned issues were included and highlighted with green color.

Line 118, page 5; "…distribution and mutations of HBV infection by two nested PCR assays, targeting S, X and pre-C…" Was this an in-house PCR assay or did the authors follow a previously published protocol – in which case this should be appropriately cited and referenced. Also, important to note is the fact that the authors have not addressed the limitations of the nested PCR assay used (vs qPCR) in the manuscript.
Response: We used a previously published protocol, and the referenced articles were cited in the table 1.
The strengths and limitations of nested PCR assay were included in the manuscript and highlighted.

Line 148, page 6; "…Logistic regression analysis was used to evaluate the risk factors…" Kindly clarify if univariate and multivariate logistic regression analyses were performed.
Response: Univariate and multivariate logistic regression analyses were performed using HBsAg and HBcAb seroprevalence as dependent variables and socio-demographic characteristics and qualitative variables (age, stage of gestation, number of pregnancies, smoking, place of residency, level of education, ethnicity, time of sampling, and history of blood transfusion, tattoo, abortion, dentistry, surgery and HBV vaccination), as independent variables.

Line 167, page 7; "…uneducated (2.4%) and had a history of tattoo (1.6%) and HBV vaccination (1.6%)." Kindly clarify in the manuscript whether participants' vaccination history was self-reported only or confirmed using clinical records. The limitations of relying on self-reported clinical history / patient recall, should be adequately addressed in the manuscript where appropriate.

Response: The participants' vaccination history was obtained according to self-declaration during the interview. This was reported in the method section and highlighted with green color.

-Unfortunately, we did not assess antibody to hepatitis B surface antigen (anti-HBsAg) among the study population due to lack of budget. This issue was mentioned as the limitation of the study and highlighted with green color, and it is suggested to study the effectiveness of the immunization policy in the pregnant population of this region.

Lines 176 – 177, page 8; "All of the HBV seropositive samples had normal levels of liver enzymes and were negative for HCV and HIV." In the methods section, the authors do not make mention of testing for liver enzymes, HIV or HCV co-infection. The authors should address how they came about these findings reported here, i.e., tested in this study, self-reported during questionnaire / interview, confirmed from reliable clinical records of participants.

Response: Levels of the liver enzymes were obtained from the records of pregnant women at the public health centers. The pregnant women attend these public health centers for periodical checkups. This was mentioned in the method section and highlighted with green color.

- The HBV seropositive pregnant women were tested for HIV and HCV coinfections by ELISA. This issue was included in the method section and highlighted with yellow color.

Lines 257 – 258, page 14; "These infected but asymptomatic pregnant women may remain undiagnosed over time." As a matter of translating research to practice, can the authors comment on the direct benefits of the study findings to the participants in this case, i.e., were hepatitis B results reported to clinicians managing participants in order to inform timely interventions?

Response: The results of this study were informed to the public health centers and the Deputy Research and Affairs of the Bushehr University of Medical Sciences. Surely, the results have been communicated to the infected pregnant women by the authorities in the public health centers. However, we are not sure about receiving treatment or care post-pregnancy by HBV positive women. Since, the follow-up of infected pregnant women was outside the scope of the study. This issue has been mentioned as a limitation of this cross-sectional study in the manuscript.

Lines 260 – 263, page 14; "Since maternal treatment with oral antivirals…HBV vaccination and immunoglobulin administration at birth…consequently, reduce the burden of chronic HBV

infection in the community [3, 16].” The authors should expand on what the national policies and guidelines in relation to these key interventions are, with an indication of the current coverage / utilization rates of these services in the country.

Response: National policy and intervention services for HBV infected pregnant women are the same as reported in the manuscript:

1) Maternal treatment with oral antivirals such as tenofovir, telbivudine and lamivudine
2) HBV vaccination and immunoglobulin administration at birth

Lines 287 – 288, page 15; “…and the sensitivity and specificity of ELISA kits in different studies can also explain these variations.” The authors should explicitly address the strengths and limitations of assays (nested PCR vs qPCR, not testing for anti-HBs or HBeAg, sensitivity and specificity of ELISA) used in this study compared to those in the aforementioned studies and how this may influence interpretation of the study findings.

Response: The strengths and limitations of nested PCR assay were included in the manuscript and highlighted (limitation section of the study).

- Sensitivity and specificity of the ELISA kits were included in the method section.

Lines 301 – 303, page 16; “In addition, HBcAb seropositivity was more prevalent in those samples collected in October...” It is unclear why the authors assessed the frequency of detection of HBV infection in relation to the months in which samples were collected. Were the authors expecting a seasonal pattern in relation to the prevalence of HBV infection? Have such seasonal patterns been demonstrated elsewhere and if so, what would the relevance of this be?

Response: We did not find a seasonal pattern regarding the seroprevalence of HBV infection among pregnant women in this region. However, according to those studies on the seasonality of hepatitis, we wanted to investigate the monthly or seasonal pattern in this study too.

Lines 309 – 310, page 16; “…a significant association was found between circumcision and HBsAg seropositivity.” With regards to the study cited, could the authors clarify if this implies male circumcision among spouses / sexual partners of pregnant women found to be HBsAg seropositive?

Response: It is female circumcision, which is usually performed at home.

Lines 330 – 331, page 17; “…undetectable levels of HBsAg and low HBV DNA levels.” Did the authors measure HBV viral loads?

Response: No, we did not measure HBV viral loads since the nested PCR assay used in this study is a qualitative method and is not able to determine the viral loads. This a limitation of nested-PCR over qPCR. The limitations of nested PCR assay were included in the manuscript.

-Those samples with very low virus levels become positive in the second round of nested PCR. This sample with occult HBV infection was identified in the second round of amplification, which is indicative of low HBV DNA levels.

-In one of our previous research projects (unpublished data), the viral load of one of the HBV DNA positive serum samples was measured by real time PCR, and the serum sample was serial diluted. The diluted serum sample with 25 HBV DNA copies/ml (5 HBV DNA copies/200 μL) became positive in the second round of nested PCR, while it was negative in the first round of PCR.

Line 340, page 17; "Notably, the sample with G1896A mutation was HBeAg negative." In the methods section the authors did not indicate serological testing for additional markers aside for HBsAg and anti-HBc (HBcAB). Could the authors address how they came about this finding in the methods section and why it was not reported as part of the results?

Response: Unfortunately, the presence of HBeAg was not investigated in the serum samples of all participants due to the lack of budget (the available funding could not cover the expenses to test all of the samples). Only the seropositive samples were tested for the presence of HBeAg. Therefore, detection of HBeAg was not mentioned in the methods section.

Lines 349 – 350, page 18; "The dominance of genotype D is the most important characteristic…in our region." In addition to the stated predominant HBV genotype in the region, the authors should indicate the HBV genotype distribution in Iran as this has not been provided as part of the introduction section.

Response: The HBV genotype distribution in Iran was provided and highlighted with green color.

Sincerely yours,

Fatemeh Farshadpour, Ph.D,
Bushehr University of Medical Sciences 7514633341
Bushehr, Iran.
00989171712653
E-mail: f.farshadpour@bpums.ac.ir
f.farshadpour@yahoo.com
