# Supplementary material for: Prevalence, genotype distribution and mutations of hepatitis B virus and the associated risk factors among pregnant women residing in the northern shores of Persian Gulf, Iran
Source: PLoS One. 2022 Mar 10;17(3):e0265063. doi: 10.1371/journal.pone.0265063 (PMC8912131; doi:10.1371/journal.pone.0265063)
Supplement: Attachment — Submitted filename: Report_Prevalence of HBV infection among pregnant women_Iran.docx [file pone.0265063.s008.docx]

**Report**

**Prevalence, genotype distribution and mutations of hepatitis B virus infection and associated risk factors among pregnant women resident in the northern shores of Persian Gulf, Iran**

**Reviewer’s Comments**

Given the increased lifetime risk for potentially fatal chronic liver disease following perinatally acquired hepatitis B virus (HBV) infection, this study sought to characterize the burden of HBV infection among pregnant women living in the South of Iran. While the findings of this study will fill a critical knowledge gap and contribute to informing interventions aimed at preventing HBV mother-to-child transmission in Iran, there is need for further clarity in the reporting of the methodology followed and results obtained in order to improve the quality of the manuscript.

Major Revisions:

- The introduction section does not address universal hepatitis B vaccination globally and in Iran, and how this has influenced the burden of disease over time. In addition, the authors do not address the availability, accessibility and utilization of maternal HBV screening and management programmes in Iran. These should be addressed to provide further clarity to the context of the study.
- In the methods section of the manuscript, the authors adequately address how socio-demographic, and clinical data were collected from study participants. However, there is no information on how blood samples were collected for the study.
- The authors allude to the use of a questionnaire in collecting relevant participant data. For validation purposes, kindly indicate of this questionnaire was piloted. Was a previously published questionnaire used during this study (in which case it should be appropriately cited and referenced) or if a study specific one was developed, can a template be provided as part of supplementary material.
- The authors have not addressed why antibodies to the hepatitis B surface antigen (anti-HBs) was not tested for among the study population. It will be interesting to understand from the introduction section how long Iran has had a universal hepatitis B vaccination programme and whether any of the younger participants should have been vaccinated in infancy. A lack of awareness of the level of immunity (either due to vaccination or recovery from a past infection) among the study population may influence interpretation of the study findings and should therefore be adequately addressed.
- The limitations of the study are not explicitly addressed in the manuscript.

Minor Revisions:

- Line 61, page 3; “HBV-related liver diseases are responsible for approximately 8,00,000 deaths…” Consider revising the number format (800 000) for better clarity.
- Lines 116 – 117, page 5; “…using commercially available ELISA kits (DIA.PRO, Milan, Italy).” For better clarity and reliability, the authors should briefly provide further information (e.g., type of platform used, automated or manual, etc) about the ELISA used. In addition, did the authors follow the manufacturer’s instructions or a published protocol (cite appropriately).
- Line 118, page 5; “…distribution and mutations of HBV infection by two nested PCR assays, targeting S, X and pre-C…” Was this an in-house PCR assay or did the authors follow a previously published protocol – in which case this should be appropriately cited and referenced. Also, important to note is the fact that the authors have not addressed the limitations of the nested PCR assay used (vs qPCR) in the manuscript.
- Line 148, page 6; “…Logistic regression analysis was used to evaluate the risk factors…” Kindly clarify if univariate and multivariate logistic regression analyses were performed.
- Line 167, page 7; “…uneducated (2.4%) and had a history of tattoo (1.6%) and HBV vaccination (1.6%).” Kindly clarify in the manuscript whether participants’ vaccination history was self-reported only or confirmed using clinical records. The limitations of relying on self-reported clinical history / patient recall, should be adequately addressed in the manuscript where appropriate.
- Lines 176 – 177, page 8; “All of the HBV seropositive samples had normal levels of liver enzymes and were negative for HCV and HIV.” In the methods section, the authors do not make mention of testing for liver enzymes, HIV or HCV co-infection. The authors should address how they came about these findings reported here, i.e., tested in this study, self-reported during questionnaire / interview, confirmed from reliable clinical records of participants.
- Lines 257 – 258, page 14; “These infected but asymptomatic pregnant women may remain undiagnosed over time.” As a matter of translating research to practice, can the authors comment on the direct benefits of the study findings to the participants in this case, i.e., were hepatitis B results reported to clinicians managing participants in order to inform timely interventions?
- Lines 260 – 263, page 14; “Since maternal treatment with oral antivirals…HBV vaccination and immunoglobulin administration at birth…consequently, reduce the burden of chronic HBV infection in the community [3, 16].” The authors should expand on what the national policies and guidelines in relation to these key interventions are, with an indication of the current coverage / utilization rates of these services in the country.
- Lines 287 – 288, page 15; “…and the sensitivity and specificity of ELISA kits in different studies can also explain these variations.” The authors should explicitly address the strengths and limitations of assays (nested PCR vs qPCR, not testing for anti-HBs or HBeAg, sensitivity and specificity of ELISA) used in this study compared to those in the aforementioned studies and how this may influence interpretation of the study findings.
- Lines 301 – 303, page 16; “In addition, HBcAb seropositivity was more prevalent in those samples collected in October...” It is unclear why the authors assessed the frequency of detection of HBV infection in relation to the months in which samples were collected. Were the authors expecting a seasonal pattern in relation to the prevalence of HBV infection? Have such seasonal patterns been demonstrated elsewhere and if so, what would the relevance of this be?
- Lines 309 – 310, page 16; “…a significant association was found between circumcision and HBsAg seropositivity.” With regards to the study cited, could the authors clarify if this implies male circumcision among spouses / sexual partners of pregnant women found to be HBsAg seropositive?
- Lines 330 – 331, page 17; “…undetectable levels of HBsAg and low HBV DNA levels.” Did the authors measure HBV viral loads?
- Line 340, page 17; “Notably, the sample with G1896A mutation was HBeAg negative.” In the methods section the authors did not indicate serological testing for additional markers aside for HBsAg and anti-HBc (HBcAB). Could the authors address how they came about this finding in the methods section and why it was not reported as part of the results?
- Lines 349 – 350, page 18; “The dominance of genotype D is the most important characteristic…in our region.” In addition to the stated predominant HBV genotype in the region, the authors should indicate the HBV genotype distribution in Iran as this has not been provided as part of the introduction section.
