# Supplementary material for: Prevalence, genotype distribution and mutations of hepatitis B virus and the associated risk factors among pregnant women residing in the northern shores of Persian Gulf, Iran
Source: PLoS One. 2022 Mar 10;17(3):e0265063. doi: 10.1371/journal.pone.0265063 (PMC8912131; doi:10.1371/journal.pone.0265063)
Supplement: Attachment — Submitted filename: Response to Reviewers.doc [file pone.0265063.s009.doc]

**ANSWERING REVIEWERS**

December 15, 2021

**Dear Editor-in-Chief of**

***PLOS ONE***

We would greatly appreciate for your consideration of the publication of our manuscript entitled "Prevalence, genotype distribution and mutations of hepatitis B virus infection and associated risk factors among pregnant women resident in the northern shores of Persian Gulf, Iran" in the *PLOS ONE*.

**Ms. No. PONE-D-21-32739**

**Title:** Prevalence, genotype distribution and mutations of hepatitis B virus infection and associated risk factors among pregnant women resident in the northern shores of Persian Gulf, Iran

**Name of Journal:** *PLOS ONE*

**Revision has been made according to the suggestions of the reviewers and the editor.**

We greatly appreciated the reviewers’ comments. The point-to-point responses to comments were shown as follows:

**Journal Requirements:**

When submitting your revision, we need you to address these additional requirements.

1. Please ensure that your manuscript meets PLOS ONE's style requirements, including those for file naming. The PLOS ONE style templates can be found at

https://journals.plos.org/plosone/s/file?id=wjVg/PLOSOne_formatting_sample_main_body.pdf

and

<https://journals.plos.org/plosone/s/file?id=ba62/PLOSOne_formatting_sample_title_authors_affiliations.pdf>

**Response:** The PLOS ONE's style requirements were applied.

2. Thank you for stating the following in the Acknowledgments Section of your manuscript:

“The authors would like to acknowledge grant number supported by the Deputy Research and 393 Affairs of the Bushehr University of Medical Sciences, Bushehr, Iran.”

We note that you have provided additional information within the Acknowledgements Section that is not currently declared in your Funding Statement. Please note that funding information should not appear in the Acknowledgments section or other areas of your manuscript. We will only publish funding information present in the Funding Statement section of the online submission form.

Please remove any funding-related text from the manuscript and let us know how you would like to update your Funding Statement. Currently, your Funding Statement reads as follows:

“This study was funded by Bushehr University of Medical Sciences with grant number 357.

The funders had no role in study design, data collection and analysis, decision to publish, or preparation of the manuscript.”

Please include your amended statements within your cover letter; we will change the online submission form on your behalf.

**Response:** The funding-related texts were removed from the Methods and the Acknowledgment sections of the manuscript. The funding information present in the Funding Statement section of the online submission form is correct.

3. PLOS ONE now requires that authors provide the original uncropped and unadjusted images underlying all blot or gel results reported in a submission’s figures or Supporting Information files. This policy and the journal’s other requirements for blot/gel reporting and figure preparation are described in detail at https://journals.plos.org/plosone/s/figures#loc-blot-and-gel-reporting-requirements and https://journals.plos.org/plosone/s/figures#loc-preparing-figures-from-image-files. When you submit your revised manuscript, please ensure that your figures adhere fully to these guidelines and provide the original underlying images for all blot or gel data reported in your submission. See the following link for instructions on providing the original image data: https://journals.plos.org/plosone/s/figures#loc-original-images-for-blots-and-gels.

In your cover letter, please note whether your blot/gel image data are in Supporting Information or posted at a public data repository, provide the repository URL if relevant, and provide specific details as to which raw blot/gel images, if any, are not available. Email us at plosone@plos.org if you have any questions.

**Response:** The original uncropped and unadjusted images underlying gel results were provided. In the original uncropped figure 1, seven positive samples are shown. Since, two positive samples (positive samples number 4 and 5) were loaded again in the wells number 12 and 13. The reason was we did not want to have empty wells, we tried to load all of the wells.

4. Your ethics statement should only appear in the Methods section of your manuscript. If your ethics statement is written in any section besides the Methods, please delete it from any other section.

**Response:** The ethics statement was removed from end of the manuscript.

5. Please include captions for your Supporting Information files at the end of your manuscript, and update any in-text citations to match accordingly. Please see our Supporting Information guidelines for more information: http://journals.plos.org/plosone/s/supporting-information.

**Response:** Captions for the Supporting Information files were included at the end of the manuscript.

**Additional Editor Comments:**
The manuscript in the present form demands a **“major revision”** before it can be published. Therefore, we invite you to submit a revised version of the manuscript that addresses the points raised during the review process.

**Response:** We greatly appreciate you taking the time to consider our manuscript while providing us constructive comments. Moreover, thank you very much for giving us the opportunity to answer the reviewers’ comments. We are really grateful for this opportunity.

1. Please ensure that your manuscript meets PLOS ONE's style requirements, including those for headings. The PLOS ONE style templates can be found at
<https://journals.plos.org/plosone/s/file?id=wjVg/PLOSOne_formatting_sample_main_body.pdf>

**Response:** The PLOS ONE's style requirements were applied.

2. Quality of written English is not suitable for publication unless extensively edited eg. The word “uneducated” should be replaced by “Illiterate”.

**Response:** The manuscript was edited for proper English language, grammar, punctuation, and spelling by our team at Bushehr University of Medical Sciences.

3. The limitations of the study are not sufficiently discussed.

**Response:** The limitations of the study were discussed in more details and highlighted.

4. Abstract needs to be re-written to reflect the study more correctly eg. Amplification of X gene is not included.

**Response:** The abstract was re-written.

**Minor comments:**
Change laboratory diagnosis sub-heading to laboratory methods.

**Response:** The change was done.

The author should consider using subheadings in the "laboratory diagnosis” section for easier reading.

Suggestion: HBV serological testing, PCR amplification and sequencing, phylogenetic and mutational analysis or sequence analysis.

**Response:** The suggestions were applied.

Include the full name of the ELISA kit used.

**Response:** The full name of the ELISA kits was included and highlighted with green color.

Table 1- Include degrees (℃) in the annealing temperature column.

**Response:** The degrees (℃) in the annealing temperature column was included.

The author indicate that the sequences has been submitted to GenBank, but no accession numbers provided.

**Response:** The accession numbers were provided and highlighted with yellow color in the result section and the legends of Fig3 and S1-S3 Figs.

The sequences have been submitted to GenBank and the accession numbers were provided via email, but they have not been released yet.

The study relied on nucleotide sequence analyses. However, there is no mention of sequencing protocol/approach/platform used in generating the nucleotide sequences in the methods section, only referenced a company that looks like it provides service.

**Response:** The Sanger dideoxy sequencing technology was included in the method section.

Table 2 and 3 should be moved to supplementary.

**Response:** The table 2 and 3 were moved to supplementary.

One of the city is stated as Khormoj in Table 2 and throughout it is stated as Khormuj.

**Response:** The spelling was corrected in the table 2.

Phylogenetic and mutational analysis (Figure 3 and Table 4) focused on 3 samples yet they detected HBV DNA in 5 samples. It is not clear whether the other 2 samples could not be sequenced or why they were left out of the analysis.

**Response:** Unfortunately, the other 2 samples had low-quality sequencing results, so that it was impossible to include them in the phylogenetic and mutational analysis.

Figure 3: Only indicate bootstrap values >70% which is considered moderate confidence.

**Response:** The phylogenetic tree was constructed by the neighbor-joining method with the Bootstrap test of phylogeny (substitutional model: maximum composite likelihood) in Molecular Evolutionary Genetics Analysis (MEGA) program version 7. Bootstrap resampling strategy and reconstruction were carried out 1,000 times to confirm the reliability of the phylogenetic tree.

**Reviewer's Responses to Questions**

**Comments to the Author**

1. Is the manuscript technically sound, and do the data support the conclusions?

The manuscript must describe a technically sound piece of scientific research with data that supports the conclusions. Experiments must have been conducted rigorously, with appropriate controls, replication, and sample sizes. The conclusions must be drawn appropriately based on the data presented.

Reviewer #1: No

Reviewer #2: Yes

Reviewer #3: Yes

Reviewer #4: Yes

2. Has the statistical analysis been performed appropriately and rigorously?

Reviewer #1: No

Reviewer #2: Yes

Reviewer #3: Yes

Reviewer #4: Yes

3. Have the authors made all data underlying the findings in their manuscript fully available?

The [PLOS Data policy](http://www.plosone.org/static/policies.action#sharing) requires authors to make all data underlying the findings described in their manuscript fully available without restriction, with rare exception (please refer to the Data Availability Statement in the manuscript PDF file). The data should be provided as part of the manuscript or its supporting information, or deposited to a public repository. For example, in addition to summary statistics, the data points behind means, medians and variance measures should be available. If there are restrictions on publicly sharing data—e.g. participant privacy or use of data from a third party—those must be specified.

Reviewer #1: Yes

Reviewer #2: Yes

Reviewer #3: Yes

Reviewer #4: Yes

4. Is the manuscript presented in an intelligible fashion and written in standard English?

PLOS ONE does not copyedit accepted manuscripts, so the language in submitted articles must be clear, correct, and unambiguous. Any typographical or grammatical errors should be corrected at revision, so please note any specific errors here.

Reviewer #1: No

Reviewer #2: Yes

Reviewer #3: Yes

Reviewer #4: Yes

**5. Review Comments to the Author**
Please use the space provided to explain your answers to the questions above. You may also include additional comments for the author, including concerns about dual publication, research ethics, or publication ethics. (Please upload your review as an attachment if it exceeds 20,000 characters)

**Reviewer #1:** The author used unacceptable language (e.g. uneducated women) which also feels derogatory. The manuscript used unnecessary statistics, while its main research was HBV infection (HBsAg or HBV DNA) or exposure as determined by HBcAb. Only 15 pregnant women were HBsAg positive but the manuscript made a lot of unnecessary and unacceptable statistical analysis (smoking, Education to mention few).

**Response:** Thank you very much for your review.

-The manuscript was edited for proper English language, grammar, punctuation, and spelling.

-These variables were included in the study in order to find the possible risk factors of HBV infection among pregnant women.

Regarding smoking: In addition to cigarette, some women in the South of Iran use hookah for smoking and they share the hookah in group. We thought that there might be a chance of HBV transmission through saliva by using hookah.

Regarding education: The education level has an indirect effect on the prevalence of HBV. The educated women have more information about the transmission routes of HBV.

Regarding the month of sampling: We assumed that there might be a seasonal or monthly pattern regarding the seroprevalence of HBV infection among pregnant women in this region.

According to those studies on the seasonality of hepatitis, we wanted to investigate the seasonal pattern in this study too.

**Reviewer #2:** This was a well-designed study that showed the prevalence and molecular characterization of HBV in pregnant woman. The literature review clearly stated the problem and the aim of the study. The methodology used was standard for this type of study.

**Response:** Thank you very much for your review and the positive comments.

In the results section, there is a comment on the occult status in Line 193, it is not shown what the viral load of this sample is in order to make such a statement. I suggest the author includes viral load of this sample.

**Response:** Occult HBV infection is described by the presence of HBV DNA in the absence of detectable HBsAg in serum or plasma. This sample, despite being positive for HBV DNA, was negative for HBsAg, which is indicative of an occult HBV infection.

-We did not measure HBV viral loads since the nested PCR assay used in this study is a qualitative method and is not able to determine the viral loads. This is a limitation of nested-PCR over quantitative PCR (qPCR). The limitations of nested PCR assay were included in the manuscript and highlighted with yellow color in the limitations section of the study.

-Those samples with very low virus levels become positive in the second round of nested PCR. This sample with occult HBV infection was identified in the second round of amplification, which is indicative of low HBV DNA levels.

- In one of our previous research projects (unpublished data), the viral load of one of the HBV DNA positive serum samples was measured by real time PCR, and the serum sample was serial diluted. The diluted sample with 25 HBV DNA copies/ml (5 HBV DNA copies/200 μL) became positive in the second round of nested PCR, while it was negative in the first round of PCR.

In the discussion, in Line 255, the author states that the HBV prevalence of 1.05% was lower than 1.18%, these are similar especially if rounded off.

**Response:** We cannot round off the numbers here. Because regarding the prevalence rate, even a small decimal is important. We used “slightly” in the sentence.

In line 258, Risk of mother to child transmission should be discussed more here, they did not show what the risk of undiagnosed HBV in pregnancy would be, especially in those infected in the third trimester.

**Response:** The mentioned issue was included and highlighted with yellow color.

Line 268, This recommendation is not clear. Do they mean that they will test each and every women in the South of Iran area? this is not cost effective, rather screen all women who come in for their first visit at the ANC and vaccinate the baby at birth. Or treat the infection if it becomes chronic.

**Response:** We agree with the dear reviewer. This sentence was revised and highlighted with green color.

Line 319, Cannot make this conclusion as this was not part of the questions asked in the questionnaire as shown in the information on Table 2 and 3. You only have vaccination data for 14 participants out of over 1000. This is being very presumptuous and this conclusion needs to be made with caution.

**Response:** We agree with the dear reviewer. Therefore, the sentence was revised and highlighted with green color.

-A significant percentage of the pregnant women in this study were not vaccinated according to self-declaration during the interview. Only 22.0% of the participants were vaccinated, 22.0% were not vaccinated and 55.9% of the participants did not respond to the questions regarding history of HBV vaccination during interview (Unknown group). These participants did not know anything about HBV vaccination. Considering the compulsory vaccination of infants since 1993 and teenagers since 2006 in Iran, individuals above 30 years were not the target group of national vaccination program. Since a high number of the “Unknown group” were above 30 years, we think that a significant percentage of the pregnant women in this study were not vaccinated. These data are shown in the tables 2 and 3.

-According to the aim of the study and the available funding, the presence of hepatitis B surface antigen (HBsAg), hepatitis B core antibody (HBcAb) and HBV viremia was investigated in serum samples of the participants. Unfortunately, we did not assess antibody to hepatitis B surface antigen (anti-HBsAg) among the study population due to lack of budget. This issue was mentioned as the limitation of the study, and it is suggested to study the effectiveness of the immunization policy in the pregnant population of this region.

Line 326, this statement contradicts what the authors had said in Line 318-320 on the ineffectiveness of the HBV vaccination program.

**Response:** The previous statement has been revised.

-The previous statement was about the seroprevalence of HBV in the age group 20-29 years old. This statement is about the pregnant women under 20 years old.

Line 331, What was the viral load of this sample? it was also not mentioned in the results section.

**Response:** We did not measure HBV viral loads since the nested PCR assay used in this study is a qualitative method and is not able to determine the viral loads. This a limitation of nested-PCR over qPCR. The limitations of nested PCR assay were included in the manuscript.

Those samples with very low virus levels become positive in the second round of nested PCR. This sample with occult HBV infection was identified in the second round of amplification, which is indicative of low HBV DNA levels.

-In one of our previous research projects (unpublished data), the viral load of one of the HBV DNA positive serum samples was measured by real time PCR, and the serum sample was serial diluted.

The diluted serum sample with 25 HBV DNA copies/ml (5 HBV DNA copies/200 μL) became positive in the second round of nested PCR, while it was negative in the first round of PCR.

Line 339-340, show the importance of this lack of HBeAg during pregnancy, this has not been discussed fully here.

**Response:** No, this sentence does not show the importance of the lack of HBeAg during pregnancy. This shows the relationship between the expression of HBeAg and immune responses to HBV infection.

It shows that the absence of HBeAg may result in evasion of immune clearance as HBeAg is a target antigen for immune recognition.

The sample with G1896A mutation was HBeAg negative. The G1896A mutation causes the conversion of TGG🡪TAG (Trp 🡪 Stop codon) at codon 28 of the pre-C gene and the suppression of HBeAg expression. This HBeAg negative sample is at the risk of liver disease progression.

In fact, it wants to show that the mutation analysis of HBV infection can be useful in predicting the disease progression in HBV-infected patients.

The Conclusion on Line 376 differs from the conclusion on the abstract. The one in the abstract lines up with what the authors set out to do, while the one in Line 376 is a repetition of what the results stated.

**Response:** The conclusion was revised and highlighted.

**Reviewer #3:** Title: suggestion, change “resident” to “residing”

**Response:** Thank you very much for your review and the constructive comments.

**-** The suggestion was applied and highlighted with yellow color.

Abstract: Page 2, line 32 please mention the name of the ELISA kit used, company and the sentence that follows should read tested for the presence of HBV DNA delete “detection”.

**Response:** The full name of the ELISA kits was included.

Page 2, line 34 the software or platforms used to analyze the mutations are not mentioned.

**Response:** The software was included.

Page 2, line 43, the sentence talking about tattooing being a risk factor should come in the conclusion. Or the sentence can either be stated as significantly associated or as risk factor not two of them.

**Response:** The abstract was revised.

Page 2, line 36, did the HBcAb positive women have HBsAg or were they only positive for HBcAb? This is because in your results you state that 2.7% of HBcAb positive were also positive for HBV DNA.

**Response:** Of 41 HBcAb seropositive women, 4 women were positive for HBsAg (HBsAg positive and HBcAb positive), and 37 women were negative for HBsAg (HBsAg negative and HBcAb positive).

Overall, 26.7% of HBsAg seropositive pregnant women and 2.7% of HBcAb seropositive women had HBV viremia with genotype D, sub-genotype D3, and subtype ayw2:

2.7% (1 case of 37 HBsAg negative and HBcAb positive cases)

26.7% (4 cases of 15 HBsAg positive cases)

Page ,2 line 46 Please indicate how many had which genotype.

**Response:** All of the HBV DNA positive samples had HBV viremia with genotype D, sub-genotype D3 and subtype ayw2.

Page 2, line 49, in the methods the authors mention the pre-core regions however in the results they mention pre-core and basal core regions.

**Response:** The target genes were included.

Page 2, line 48, it is important to note that the mutations were detected in the same sample deemed as an occult, it is not clear in the statement.

**Response:** The mutations were not specific to one sample. The mutations were detected in the S, X, BCP and pre-C regions of the HBV genome isolated from pregnant women and were not specific to the occult HBV sample.

According to mutations analyses, seven amino acid substitutions in the HBsAg, one point mutation in the pre-C region and five points mutations in the basal core promoter (BCP) region were detected. Besides, the BCP mutations caused amino acid substitutions in the X protein.

Page 2, line 50 to 53, the authors are not concluding on their findings but are making a general remark. First it should be clear if HBV is endemic or not in this region and if perinatal transmission is a problem or not. In their results the authors found a very low prevalence, they should say something about it in their conclusion.

**Response:** The conclusion was revised.

Page 4, line 95, please add the information about the target genes missing in the abstract.

**Response:** The target genes were included.

Page 7, line 167, means 1.6% of the vaccinated women had chronic or acute infection.

**Response:** Yes, it means 1.6% of the vaccinated women were HBsAg seropositive, or the seroprevalence of HBsAg among vaccinated women was 1.6%.

Page 8, line 177, the authors did not mention, any liver enzyme tests performed however they are mentioning them in their results.

**Response:** Levels of the liver enzymes were obtained from the records of pregnant women at the public health centers. The pregnant women attend these public health centers for periodical checkups.

Page 8, line 177-179, the tables should also be referred to in the text.

**Response:** The tables were referred to in the text.

Page 11, line 190, a suggestion to delete “evaluation” to “testing”

**Response:** The suggestion was applied.

Page 11, line 190 the authors should stick to one decimal after the comma throughout the document for consistency.

**Response:** We cannot round off the numbers here. Because regarding the prevalence rate, even a small decimal is important.

Page 14, line 258 to 263 the sentence is too long.

**Response:** The long sentence was rephrased.

Why did the authors not check the women vaccination status? Why did the authors not test for Anti-HBs but the authors are making a statement in the discussion that majority of women are not vaccinated in this region? Do the authors mean to tell us that there is no HBV routine testing in pregnant women in this region?

**Response:** A significant percentage of the pregnant women in this study were not vaccinated according to self-declaration during the interview. Only 22.0% of the participants were vaccinated, 22.0% were not vaccinated and 55.9% of the participants did not respond to the questions regarding history of HBV vaccination during interview (Unknown group). These participants did not know anything about HBV vaccination. Considering the compulsory vaccination of infants since 1993 and teenagers since 2006 in Iran, individuals above 30 years were not the target group of national vaccination program. Since a high number of the “Unknown group” were above 30 years, we think that a significant percentage of the pregnant women in this study were not vaccinated. These data are shown in the tables 2 and 3.

-According to the aim of the study and the available funding, the presence of hepatitis B surface antigen (HBsAg), hepatitis B core antibody (HBcAb) and HBV viremia was investigated in serum samples of the participants. Unfortunately, we did not assess antibody to hepatitis B surface antigen (anti-HBsAg) among the study population due to lack of budget. However, we agree with the dear reviewer that the lack of awareness of the level of immunity among the study population can influence interpretation of the study findings. This issue was mentioned as the limitation of the study, and it is suggested to study the effectiveness of the immunization policy in the pregnant population of this region.

-Yes, there is no guideline or national policy on HBV screening during pregnancy. In fact, this study was performed to convince the health care providers to include HBV screening in the routine screening of pregnant women in Iran. Currently, pregnant women are only screened for rubella and CMV in Iran.

Page 16, line 319-320 the authors cannot claim that the HBV vaccination programs are not effective while they did not report if these people had received the vaccine or not and check why if they did not received the vaccine?

**Response:** We agree with the dear reviewer. Therefore, the sentence was revised and highlighted with green color.

- This issue was mentioned as the limitation of the study, and it is suggested to study the effectiveness of the immunization policy in the pregnant population of this region.

Page 17, line 323, how did the authors arrive at a conclusion that the HBV management during pregnancy is facing challenges?

**Response:** Because although there are HBV vaccine and oral antivirals and screening tools for the prevention, treatment and diagnosis of HBV infection, the status of immunity and prevalence of HBV infection among pregnant population in Iran is unknown. Since, there is no guideline or national policy on HBV screening during pregnancy. We need such kind of studies among pregnant women to convince the health care providers to include HBV screening in the management program of pregnant women in Iran.

Page 17, line 332 please rephrase the sentence.

**Response:** The sentence was rephrased and highlighted with yellow color.

This sample was found to be positive in the second round of PCR; this statement is irrelevant in the discussion.

**Response:** The sentence was removed.

Page 17, line 340, the authors state that one sample was negative for HBeAg however this was not mentioned in the methods that was tested in any sample.

**Response:** Unfortunately, the presence of HBeAg was not investigated in the serum samples of all participants due to the lack of budget (the available funding could not cover the expenses to test all of the samples). Only the seropositive samples were tested for the presence of HBeAg. Therefore, detection of HBeAg was not mentioned in the methods section.

Page 19, line 374, the statement is not clear.

**Response:** Due to the cross-sectional design of the study, we could not determine the possible effects of hepatitis B on pregnancy outcomes. Therefore, prospective or longitudinal studies are required to determine the possible effects of hepatitis B on pregnancy outcomes among pregnant women in the South of Iran.

Prospective studies are required to complete our study regarding the possible effects of hepatitis B on pregnancy outcomes.

General comments
The authors should try to keep their sentences short.

**Response:** The long sentences were rephrased.

The pictures are not clear; they are not of good quality.

**Response:** The pictures with high quality were provided.

The authors in their conclusion make no mention of the risk factors associated with HBV infection in their study.

**Response:** The conclusion was revised.

The authors can summarize the results section in the abstract.

**Response:** The abstract was revised.

Information on vaccination in the study area is not mentioned in the introduction, the vaccination coverage, when did the vaccination starts, which age group is targeted, the HBV testing program for pregnant women in this area is also missing in the introduction. These informs the reader about the situation in the study area and supports why study was conducted.

**Response:** The information on vaccination is mentioned in the introduction and highlighted with yellow color.

-There is no national policy on HBV screening during pregnancy in Iran. In fact, this study was performed to convince the health care providers to include HBV screening in the routine screening of pregnant women in Iran. Currently, pregnant women are only screened for rubella and CMV in Iran.

There is no made mention of how the sample size was calculated only how the study sites were identified.

**Response:** This is a cross-sectional study that was performed from January 2018 to June 2019.

During the study period, all pregnant women who agreed to participate and gave written informed consent to use their samples for HBV detection and their data for analysis were included consecutively in this study.

-This has been mentioned in the methods section: “From January 2018 to June 2019, all the pregnant women attending these public health centers for routine visits were included consecutively in this study. The pregnant women gave written informed consent to participate in the study and use their serum samples for HBV detection.”

**Reviewer #4:** While the findings of this study will fill a critical knowledge gap and contribute to informing interventions aimed at preventing HBV mother-to-child transmission in Iran, there is need for further clarity in the reporting of the methodology followed and results obtained in order to improve the quality of the manuscript. In addition, the limitations of the study are not explicitly addressed in the manuscript.

**Response:** Thank you very much for your review and the positive comments. The mentioned issues were included and highlighted.

**Reviewer’s Comments**

Given the increased lifetime risk for potentially fatal chronic liver disease following perinatally acquired hepatitis B virus (HBV) infection, this study sought to characterize the burden of HBV infection among pregnant women living in the South of Iran. While the findings of this study will fill a critical knowledge gap and contribute to informing interventions aimed at preventing HBV mother-to-child transmission in Iran, there is need for further clarity in the reporting of the methodology followed and results obtained in order to improve the quality of the manuscript.

**Response:** Thank you very much for your review and the positive comments.

**Major Revisions:**

The introduction section does not address universal hepatitis B vaccination globally and in Iran, and how this has influenced the burden of disease over time. In addition, the authors do not address the availability, accessibility and utilization of maternal HBV screening and management programmes in Iran. These should be addressed to provide further clarity to the context of the study.

**Response:** The mentioned issue was included and highlighted with yellow color in the introduction section.

-Unfortunately, there is no guideline or national policy on HBV screening during pregnancy. In fact, this study was performed to convince the health care providers to include HBV screening in the routine screening of pregnant women in Iran. Currently, pregnant women are only screened for rubella and CMV in Iran.

In the methods section of the manuscript, the authors adequately address how socio-demographic, and clinical data were collected from study participants. However, there is no information on how blood samples were collected for the study.

**Response:** The leftover serum samples of pregnant women who attend the public health centers for routine checkups were used for HBV detection.

The authors allude to the use of a questionnaire in collecting relevant participant data. For validation purposes, kindly indicate of this questionnaire was piloted. Was a previously published questionnaire used during this study (in which case it should be appropriately cited and referenced) or if a study specific one was developed, can a template be provided as part of supplementary material.

**Response:** The socio-demographic characteristics and pregnancy information were obtained by interviewing each pregnant woman using a questionnaire (a study specific one was developed). The questionnaire was translated to English and provided as supplementary.

The authors have not addressed why antibodies to the hepatitis B surface antigen (anti-HBs) was not tested among the study population. It will be interesting to understand from the introduction section how long Iran has had a universal hepatitis B vaccination programme and whether any of the younger participants should have been vaccinated in infancy. A lack of awareness of the level of immunity (either due to vaccination or recovery from a past infection) among the study population may influence interpretation of the study findings and should therefore be adequately addressed.

**Response:** According to the aim of the study and the available funding, the presence of hepatitis B surface antigen (HBsAg), hepatitis B core antibody (HBcAb) and HBV viremia was investigated in serum samples of the participants. Unfortunately, we did not assess antibody to hepatitis B surface antigen (anti-HBsAg) among the study population due to lack of budget. However, we agree with the dear reviewer that the lack of awareness of the level of immunity among the study population can influence interpretation of the study findings. This issue was mentioned as the limitation of the study, and it is suggested to study the effectiveness of the immunization policy in the pregnant population of this region.

The limitations of the study are not explicitly addressed in the manuscript.

**Response:** The limitations of the study were discussed in more details and highlighted.

**Minor Revisions:**

Line 61, page 3; “HBV-related liver diseases are responsible for approximately 8,00,000 deaths…” Consider revising the number format (800 000) for better clarity.

**Response:** The number format was revised.

Lines 116 – 117, page 5; “…using commercially available ELISA kits (DIA.PRO, Milan, Italy).” For better clarity and reliability, the authors should briefly provide further information (e.g., type of platform used, automated or manual, etc) about the ELISA used. In addition, did the authors follow the manufacturer’s instructions or a published protocol (cite appropriately).

**Response:** The mentioned issues were included and highlighted with green color.

Line 118, page 5; “…distribution and mutations of HBV infection by two nested PCR assays, targeting S, X and pre-C…” Was this an in-house PCR assay or did the authors follow a previously published protocol – in which case this should be appropriately cited and referenced. Also, important to note is the fact that the authors have not addressed the limitations of the nested PCR assay used (vs qPCR) in the manuscript.

**Response:** We used a previously published protocol, and the referenced articles were cited in the table 1.

The strengths and limitations of nested PCR assay were included in the manuscript and highlighted.

Line 148, page 6; “…Logistic regression analysis was used to evaluate the risk factors…” Kindly clarify if univariate and multivariate logistic regression analyses were performed.

**Response:** Univariate and multivariate logistic regression analyses were performed using HBsAg and HBcAb seroprevalence as dependent variables and socio-demographic characteristics and qualitative variables (age, stage of gestation, number of pregnancies, smoking, place of residency, level of education, ethnicity, time of sampling, and history of blood transfusion, tattoo, abortion, dentistry, surgery and HBV vaccination), as independent variables.

Line 167, page 7; “…uneducated (2.4%) and had a history of tattoo (1.6%) and HBV vaccination (1.6%).” Kindly clarify in the manuscript whether participants’ vaccination history was self-reported only or confirmed using clinical records. The limitations of relying on self-reported clinical history / patient recall, should be adequately addressed in the manuscript where appropriate.

**Response:** The participants’ vaccination history was obtained according to self-declaration during the interview. This was reported in the method section and highlighted with green color.

-Unfortunately, we did not assess antibody to hepatitis B surface antigen (anti-HBsAg) among the study population due to lack of budget. This issue was mentioned as the limitation of the study and highlighted with green color, and it is suggested to study the effectiveness of the immunization policy in the pregnant population of this region.

Lines 176 – 177, page 8; “All of the HBV seropositive samples had normal levels of liver enzymes and were negative for HCV and HIV.” In the methods section, the authors do not make mention of testing for liver enzymes, HIV or HCV co-infection. The authors should address how they came about these findings reported here, i.e., tested in this study, self-reported during questionnaire / interview, confirmed from reliable clinical records of participants.

**Response:** Levels of the liver enzymes were obtained from the records of pregnant women at the public health centers. The pregnant women attend these public health centers for periodical checkups. This was mentioned in the method section and highlighted with green color.

- The HBV seropositive pregnant women were tested for HIV and HCV coinfections by ELISA. This issue was included in the method section and highlighted with yellow color.

Lines 257 – 258, page 14; “These infected but asymptomatic pregnant women may remain undiagnosed over time.” As a matter of translating research to practice, can the authors comment on the direct benefits of the study findings to the participants in this case, i.e., were hepatitis B results reported to clinicians managing participants in order to inform timely interventions?

**Response:** The results of this study were informed to the public health centers and the Deputy Research and Affairs of the Bushehr University of Medical Sciences. Surely, the results have been communicated to the infected pregnant women by the authorities in the public health centers. However, we are not sure about receiving treatment or care post-pregnancy by HBV positive women. Since, the follow-up of infected pregnant women was outside the scope of the study. This issue has been mentioned as a limitation of this cross-sectional study in the manuscript.

Lines 260 – 263, page 14; “Since maternal treatment with oral antivirals…HBV vaccination and immunoglobulin administration at birth…consequently, reduce the burden of chronic HBV infection in the community [3, 16].” The authors should expand on what the national policies and guidelines in relation to these key interventions are, with an indication of the current coverage / utilization rates of these services in the country.

**Response:** National policy and intervention services for HBV infected pregnant women are the same as reported in the manuscript:

1) Maternal treatment with oral antivirals such as tenofovir, telbivudine and lamivudine
2) HBV vaccination and immunoglobulin administration at birth

Lines 287 – 288, page 15; “…and the sensitivity and specificity of ELISA kits in different studies can also explain these variations.” The authors should explicitly address the strengths and limitations of assays (nested PCR vs qPCR, not testing for anti-HBs or HBeAg, sensitivity and specificity of ELISA) used in this study compared to those in the aforementioned studies and how this may influence interpretation of the study findings.

**Response:** The strengths and limitations of nested PCR assay were included in the manuscript and highlighted (limitation section of the study).

- Sensitivity and specificity of the ELISA kits were included in the method section.

Lines 301 – 303, page 16; “In addition, HBcAb seropositivity was more prevalent in those samples collected in October...” It is unclear why the authors assessed the frequency of detection of HBV infection in relation to the months in which samples were collected. Were the authors expecting a seasonal pattern in relation to the prevalence of HBV infection? Have such seasonal patterns been demonstrated elsewhere and if so, what would the relevance of this be?

**Response:** We did not find a seasonal pattern regarding the seroprevalence of HBV infection among pregnant women in this region. However, according to those studies on the seasonality of hepatitis, we wanted to investigate the monthly or seasonal pattern in this study too.

Lines 309 – 310, page 16; “…a significant association was found between circumcision and HBsAg seropositivity.” With regards to the study cited, could the authors clarify if this implies male circumcision among spouses / sexual partners of pregnant women found to be HBsAg seropositive?

**Response:** It is female circumcision, which is usually performed at home.

Lines 330 – 331, page 17; “…undetectable levels of HBsAg and low HBV DNA levels.” Did the authors measure HBV viral loads?

**Response:** No, we did not measure HBV viral loads since the nested PCR assay used in this study is a qualitative method and is not able to determine the viral loads. This a limitation of nested-PCR over qPCR. The limitations of nested PCR assay were included in the manuscript.

-Those samples with very low virus levels become positive in the second round of nested PCR. This sample with occult HBV infection was identified in the second round of amplification, which is indicative of low HBV DNA levels.

-In one of our previous research projects (unpublished data), the viral load of one of the HBV DNA positive serum samples was measured by real time PCR, and the serum sample was serial diluted. The diluted serum sample with 25 HBV DNA copies/ml (5 HBV DNA copies/200 μL) became positive in the second round of nested PCR, while it was negative in the first round of PCR.

Line 340, page 17; “Notably, the sample with G1896A mutation was HBeAg negative.” In the methods section the authors did not indicate serological testing for additional markers aside for HBsAg and anti-HBc (HBcAB). Could the authors address how they came about this finding in the methods section and why it was not reported as part of the results?

**Response:** Unfortunately, the presence of HBeAg was not investigated in the serum samples of all participants due to the lack of budget (the available funding could not cover the expenses to test all of the samples). Only the seropositive samples were tested for the presence of HBeAg. Therefore, detection of HBeAg was not mentioned in the methods section.

Lines 349 – 350, page 18; “The dominance of genotype D is the most important characteristic…in our region.” In addition to the stated predominant HBV genotype in the region, the authors should indicate the HBV genotype distribution in Iran as this has not been provided as part of the introduction section.

**Response:** The HBV genotype distribution in Iran was provided and highlighted with green color.

Sincerely yours,

Fatemeh Farshadpour, Ph.D,

Bushehr University of Medical Sciences 7514633341

Bushehr, Iran.

00989171712653

E-mail: f.farshadpour@bpums.ac.ir

f.farshadpour@yahoo.com
