# Supplementary material for: Prevalence, genotype distribution and mutations of hepatitis B virus and the associated risk factors among pregnant women residing in the northern shores of Persian Gulf, Iran
Source: PLoS One. 2022 Mar 10;17(3):e0265063. doi: 10.1371/journal.pone.0265063 (PMC8912131; doi:10.1371/journal.pone.0265063)
Supplement: Attachment — Submitted filename: Response to Reviewers 2.doc [file pone.0265063.s011.doc]

**ANSWERING REVIEWERS**

February 7, 2022

**Dear Editor-in-Chief of**

***PLOS ONE***

We would greatly appreciate for your consideration of the publication of our manuscript entitled "Prevalence, genotype distribution and mutations of hepatitis B virus infection and associated risk factors among pregnant women resident in the northern shores of Persian Gulf, Iran" in the *PLOS ONE*.

**Ms. No. PONE-D-21-32739R1**

**Title:** Prevalence, genotype distribution and mutations of hepatitis B virus infection and associated risk factors among pregnant women resident in the northern shores of Persian Gulf, Iran

**Name of Journal:** *PLOS ONE*

**Revision has been made according to the suggestions of the reviewers.**

We greatly appreciated the reviewers’ comments. The point-to-point responses to comments were shown as follows:

**Journal Requirements:**

Please review your reference list to ensure that it is complete and correct. If you have cited papers that have been retracted, please include the rationale for doing so in the manuscript text, or remove these references and replace them with relevant current references. Any changes to the reference list should be mentioned in the rebuttal letter that accompanies your revised manuscript. If you need to cite a retracted article, indicate the article’s retracted status in the References list and also include a citation and full reference for the retraction notice.

**Response:** The reference list was checked.

**Review Comments to the Author**
Reviewer #3: Dr RL Lebelo review
Thank you to the Authors for addressing the comments and suggestions that I have made to the manuscript. Thank you for improving on your work overall presentation. I am happy with the paper however I have few comments and further comets on some previous review.

**Response:** Thank you very much for your review.

Title: Prevalence, genotype distribution and mutations of hepatitis B virus infection and associated risk factors among pregnant women residing in the northern shores of Persian Gulf, Iran.
On the title the mutations cannot be of infection but of the virus thus the title should be reworded.

**Response:** “infection” was removed.

I suggest that after ‘and’ before associated the authors add “the”, the same should be done in page 2 line 28.

**Response:** The suggestion was applied.

Page 2, line 48, it is important to note that the mutations were detected in the same sample deemed as an occult, it is not clear in the statement.
Response: The mutations were not specific to one sample. The mutations were detected in the S,
X, BCP and pre-C regions of the HBV genome isolated from pregnant women and were not specific to the occult HBV sample.
According to mutations analyses, seven amino acid substitutions in the HBsAg, one point mutation in the pre-C region and five points mutations in the basal core promoter (BCP) region were detected. Besides, the BCP mutations caused amino acid substitutions in the X protein.
Reviewer: the authors still do not show which mutations were found in the sample deemed occult. The statement is not clear meaning all samples showed the same mutations on all genes targeted.

**Response:** No, all samples did not show the same mutations on all genes targeted. The specific mutations of each sample have been shown in Table 2 and S1-S3 figs. The detailed information regarding the mutations in each sample were added to the result section of the manuscript.

“The conversion of Ala 🡪 Val at amino acid 168 (A168V) and Thr 🡪 Pro at amino acid 127 (T127P) were detected in HBsAg of the occult HBV strain.” This sentence was added to the abstract section.

Page 2, line 36, did the HBcAb positive women have HBsAg or were they only positive for
HBcAb? This is because in your results you state that 2.7% of HBcAb positive were also positive for HBV DNA.
Response: Of 41 HBcAb seropositive women, 4 women were positive for HBsAg (HBsAg positive and HBcAb positive), and 37 women were negative for HBsAg (HBsAg negative and HBcAb positive).
Overall, 26.7% of HBsAg seropositive pregnant women and 2.7% of HBcAb seropositive women had HBV viremia with genotype D, sub-genotype D3, and subtype ayw2:
2.7% (1 case of 37 HBsAg negative and HBcAb positive cases)
26.7% (4 cases of 15 HBsAg positive cases)
Reviewer: Page 13 line 227, can the authors add the percentages on these sentences because they are confusing where they are placed. Then the authors can mention the genotypes without the percentages.

**Response:** The paragraph was revised.

In page 6 line 128-129 the authors make mention of the act that seropositive women were tested for HIV and HCV, was this part of this manuscript? Or another study? This is because the results section in the abstract makes no mention of these results.

**Response:** This is a part of this study. Due to limitation of word count in the abstract section, this part had not been added to the abstract section. This part was added to the abstract section.

Page 6 line 132 the authors mention two nested PCR assay however the author only make mention of one PCR assay targeting the S gene.

**Response:** Two nested PCR assays were described in the manuscript. The first nested PCR assay has been mentioned in the “PCR amplification and sequencing” section. The second nested PCR assay has been mentioned in the “Phylogenetic and mutational analysis” section. This is due to the request of one of the reviewers. Figures 2 and 3 show the electrophoresis of PCR products of these two PCR assays.

Page 7 line 148-153 falls under the title “PCR amplification and sequencing” from line 153 to 156 should be added to the text under phylogenetic and mutational analysis and rephrased.

**Response:** The suggestion was applied. This was due to the request of one of the reviewers.

Page 8, line 177, the authors did not mention, any liver enzyme tests performed however they are mentioning them in their results.
Response: Levels of the liver enzymes were obtained from the records of pregnant women at the public health centers. The pregnant women attend these public health centers for periodical checkups.
Reviewer: can this statement be added in the methods.

**Response:** This sentence was added to the method section.

Page 8 line 179 after (68.4%) the sentence is unclear and should be rephrased.

**Response:** It means 68.4%, 55.2%, 97.1% and 90.0% of women were in the third trimester of pregnancy, the age group 20–29 years, educated and Fars, respectively. On the other word, the majority of pregnant women were in the third trimester of pregnancy, the age group 20–29 years, educated, and Fars.

Page 17, line 340, the authors state that one sample was negative for HBeAg however this was not mentioned in the methods that was tested in any sample.
Response: Unfortunately, the presence of HBeAg was not investigated in the serum samples of all participants due to the lack of budget (the available funding could not cover the expenses to test all of the samples). Only the seropositive samples were tested for the presence of HBeAg.
Therefore, detection of HBeAg was not mentioned in the methods section.
Reviewer: it does not matter how many samples were tested but the reason behind the selection of the samples tested should be mentioned otherwise the authors should remove the statement about HBeAg from the results.

**Response:** The detection of HBeAg was added to the method section.

Page 16 line 368, the authors can according to literature discuss the outcomes of the children infected with the genotypes detected and the implications of the mutations found if they were to be transmitted to the children. I therefor feel that the authors are discussing the results and not discussing the results looking at the population and the implications of the infections transmitted to the children.

**Response:** Since we could not follow the infants born to infected mothers due to the cross-sectional design of the study, we were not able to determine the eventual effects of hepatitis B on pregnancy outcomes in this study. This part has been mentioned as a limitation of this study. Nevertheless, the overall effects of the detected genotype and mutations in this study have been discussed in the discussion section. These parts were highlighted with yellow color.

“The BCP and pre-C mutations are associated with the progression of chronic HBV infection to advanced liver disease and are frequently found in patients with chronic hepatitis, fibrosis, liver cirrhosis, and HCC.”

“Genotype D is characterized by chronicity, worse clinical outcomes (cirrhosis and HCC), low response to IFN-based therapy, and a high frequency of BCP and pre-C mutations”

Page 17 line 390, the “of HBV-infected women” should come after HBV vaccination so that the recommended can include vaccination of the infected women not just vaccination.

**Response:** The suggestion was applied.

The last comment is based on vaccinated in Iran yes there is vaccination in infants does the country do a birth dose of vaccine? This should be included in the discussion as a way to reduce the prevalence.

**Response:** Since 1993, infants receive HBV vaccine at birth in Iran. This has been mentioned at the introduction section. This sentence was highlighted with yellow color.

“The initiation of HBV vaccination of infants since 1993 and teenagers since 2006 in the country has had a significant role in reducing the prevalence of HBV infection in the community.”

Reviewer #4: The authors have adequately considered the comments made in the preliminary review and revising the manuscript. I propose that the manuscript be reviewed for minor grammatical and typographical errors to enhance clarity and improve readability.

**Response:** Thank you very much for your review. The manuscript was edited for proper English language, grammar, punctuation, and spelling by our team at Bushehr University of Medical Sciences. The changes have been shown by “Track changes”.

Sincerely yours,

Fatemeh Farshadpour, Ph.D,

Bushehr University of Medical Sciences 7514633341

Bushehr, Iran.

00989171712653

E-mail: f.farshadpour@bpums.ac.ir

f.farshadpour@yahoo.com
